# Mechanochemical Synthesis, Characterization and Reactivity of a Room Temperature Stable Calcium Electride

**DOI:** 10.1021/jacs.4c09408

**Published:** 2024-10-08

**Authors:** Alex W. J. Bowles, James A. Quirk, Yu Liu, George H. Morritt, Marina Freitag, George F. S. Whitehead, Adam W. Woodward, Adam Brookfield, Conrad A. P. Goodwin, David Collison, Floriana Tuna, Claire L. McMullin, James A. Dawson, Erli Lu, Fabrizio Ortu

**Affiliations:** † School of Chemistry, University of Leicester, University Road, Leicester, LE1 7RH, U.K.; ‡ Chemistry − School of Natural and Environmental Sciences, Newcastle University, Newcastle upon Tyne, NE1 7RU, U.K.; § School of Mathematics, Statistics, and Physics, Newcastle University, Newcastle upon Tyne, NE1 7RU, U.K.; ∥ Department of Chemistry, The University of Manchester, Manchester, M13 9PL, U.K.; ⊥ Department of Chemistry and Photon Science Institute, The University of Manchester, Manchester, M13 9PL, U.K.; # Department of Chemistry, University of Bath, Claverton Down, Bath, BA2 7AY, U.K.; ¶ School of Chemistry, University of Birmingham, Edgbaston, Birmingham, B15 2TT, U.K.

## Abstract

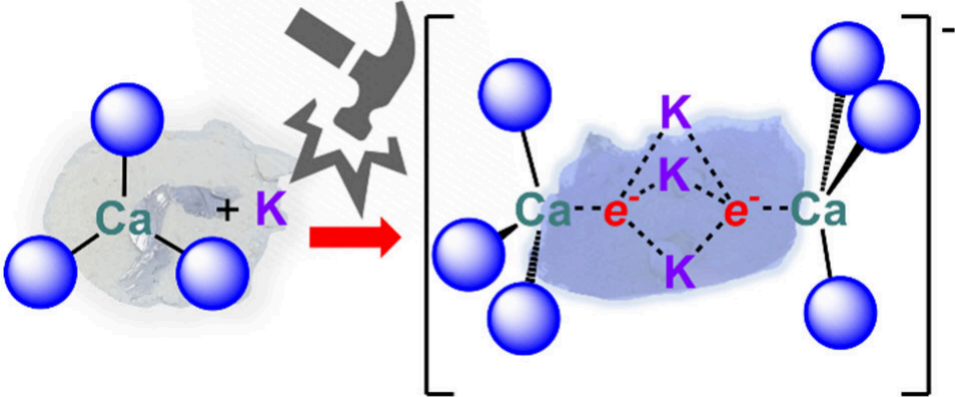

A new calcium-based Room temperature Stable Electride (RoSE), K[{Ca[N(Mes)(SiMe_3_)]_3_(*e*^*–*^)}_2_K_3_] (**2**), is successfully synthesized from the reaction of a calcium tris-amide, [Ca{N(Mes)(SiMe_3_)}_3_K] (**1**) (Mes = 2,4,6-trimethylphenyl), with potassium under mechanochemical treatment. The dimeric structure of K[{Ca[N(Mes)(SiMe_3_)]_3_(*e*^*–*^)}_2_K_3_] is calculated using *ab initio* random structure searching (AIRSS) methods. This shows the existence of highly localized anionic electrons (*e*^*–*^) and suggests poor electrical conductance, as confirmed via electroconductivity measurements. The two anionic electrons in **2** are strongly antiferromagnetically coupled, thus in agreement with the largely diamagnetic response from magnetometry. Reaction of **2** with pyridine affords 4,4′-bipyridine, while reaction with benzene gives C–H activation and formation of a calcium hydride complex, [K(η^6^-C_6_H_6_)_4_][{Ca[N(Mes)(SiMe_3_)](H)}_2_K_3_] (**3**). Computational DFT analysis reveals the crucial role played by the ligand framework in the stabilization of this new Ca-hydride complex.

## Introduction

**Figure 1:**
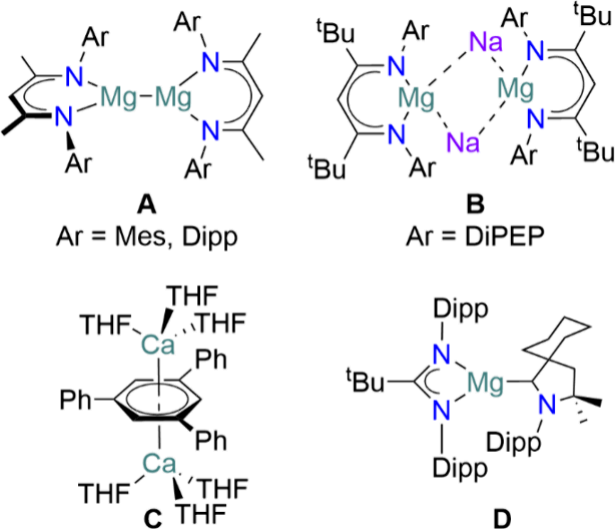
Landmark low oxidation state AE compounds **A**–**D**. Dipp = 2,6-diisopropylphenyl; Mes = 2,4,6-trimethylphenyl; DiPEP = 2,6-diisopentylphenyl.

The chemistry of the alkaline earth (AE) metals is classically dominated by the +2 oxidation state owing to the closed-shell configuration of AE(II) cations. In 2007, Jones and co-workers isolated the first formal AE(I) species in the form of Mg(I) dimers supported by large β-diketiminate (^R^BDI, {CH[C(Me)N-Ar]_2_}^−^, Ar = aryl; **A**, Figure 1) or guanidinate ligands.^1^ Since this discovery, low oxidation state magnesium chemistry has flourished and has recently been expanded to the zero oxidation state by Harder and co-workers (**B**, Figure 1).^2^ Mg(I) complexes have established themselves as soluble reducing agents, which can be employed effectively against a variety of substrates and small molecules.^3−5^ Despite these successes, attempts to extend the same methodologies to the heavier Group 2 congeners (Ca–Ba) have not been successful due to the synthetic challenges associated with these species (*e.g*. Schlenk equilibrium, large ionic radii, electropositive character, solubility).^6,7^ To date, the serendipitous Ca(I) inverse-sandwich complex reported by Westerhausen and co-workers (**C**, Figure 1) represents the only example of a low oxidation state complex of the heavier AE metals, though the formal assignment of the oxidation state of the calcium cations in **C** is disputed.^8,9^ Furthermore, there are no examples of clearly defined, monomeric, low oxidation state AE complexes, though there are several examples of AE(I) and AE(0) “synthons”. Recent examples of AE(I) synthons are AE(II) bimetallic complexes bridged by {C_6_H_6_}^2–^ or {N_2_}^2–^ anions,^10−14^ while typical AE(0) synthons are anthracene complexes, *e.g*. [Mg(C_14_H_10_)(THF)_3_]^15^ and [Ba{C_14_H_8_(SiMe_3_)_2_}(Et_2_O)].^16^

Previously, some of us reported a blueprint model toward the stabilization of monometallic complexes of the heavier AE metals (Ca–Ba) in the +1 oxidation state, involving the reduction of AE(II) tris(amide) precursors that feature the central metal in a highly equatorial, planar coordination environment.^17^ This hypothesis is supported by an extensive DFT study, which found *d*-orbital character in the LUMO of the AE(II) precursors and in the SOMO of the theoretically reduced AE(I) dianions. Despite the promise of this model, no such AE(I) species have yet been isolated via conventional solution reduction protocols.

In recent years, mechanochemical approaches have been employed to perform established transformations such as the preparation of Grignard reagents in air and solvent-free Birch reductions, in both cases offering a more environmentally friendly and synthetically easier approach than classical methods.^18−20^ Moreover, by removing solvents mechanochemical synthesis offers a route to access highly reactive species incompatible with solvents. In Group 2 chemistry, Harder and co-workers reported a magnesium-based radical species [Mg{^t^BuC(NDipp)_2_}(CAAC)^•^] (**D**, Figure 1; CAAC = cyclic (alkyl)(amino)carbene) obtained from the mechanochemical reduction of [Mg{^t^BuC(NDipp)_2_}(CAAC)(I)]. Crucially, reductions carried out with standard solution methods did not lead to the isolation of **D**.^21,22^ In more recent work Harder and co-workers have also attempted the mechanochemical reduction of [{Ca(^DiPEP^BDI)(μ-I)(THF)}_2_] (DiPEP = 2,6-(3-pentyl)-phenyl) with K/KI, leading to the formation of a deep purple powder that the authors assigned as the radical species “^•^Ca(^DiPEP^BDI)(THF)”, though this could not be fully characterized.^10^ This compound reacts readily with benzene to form [{Ca(^DiPEP^BDI)(THF)}_2_(Ph-Ph)], together with [{Ca(^DiPEP^BDI)(μ-H)(THF)}_2_] and [{Ca(^DiPEP^BDI)(μ-Ph)}_2_].^10^ Some of us reported the reduction of Li[N(SiMe_3_)_2_] with metallic K under mechanochemical conditions, which led to the isolation of a Room temperature Stable Electride (RoSE), K^+^[Li(N(SiMe_3_)_2_)]*e*^*–*^.^11^ Such species react with almost all solvents, and are therefore impossible to obtain via traditional “wet” (*i.e*. solution) synthetic protocols. Electrides are materials that feature stoichiometric, “anionic electrons” that are trapped in well-defined cavities, and typically exhibit high magnetic susceptibilities and conductivities.^23,24^ These anionic electrons do not belong to atoms or covalent bonds, and they act as anions that neutralize the charge of cations, despite not actually being incorporated into an ion.^25^ These materials can also display great synthetic utility, and, for example, have been intensively researched in recent years as supporting materials for transition metal catalysts used in ammonia synthesis.^26^ Numerous examples of crystalline electrides have been reported since the isolation of Cs^+^[18-crown-6]_2_e^–^ by Dye and co-workers in 1983. These compounds are usually unstable under ambient conditions and initially could only be isolated at low temperatures.^27−31^ The first RoSE, [Ca_24_Al_28_O_68_]^4+^[4e^–^], was reported in 2003, and was synthesized by reducing 12CaO·7Al_2_O_3_ under very harsh reaction conditions.^32,33^ Dye and co-workers were successful in the preparation of a RoSE in 2005 through implementation of aza-macrocyclic sequestering agents in the ionic lattice.^34^ Most notably, the heterobimetallic Group 1 metal RoSE K^+^[Li(N(SiMe_3_)_2_)]*e*^*–*^ was obtained via mechanochemical synthesis, and unlocked unique reactivity such as the possibility of selectively reducing either Li^+^ or K^+^ by using different Lewis base donors.^35^

Given the literature precedent, we decided to attempt the reduction of our AE(II) precursors via ball-milling, thus eliminating possible degradation pathways associated with the use of solvents. Herein, we report the mechanochemical reduction of a Ca(II) tris(amide) complex [Ca{N(Mes)(SiMe_3_)}_3_K] and the serendipitous synthesis and characterization of a very rare example of a Ca-containing RoSE. The reactivity of this new RoSE was tested toward simple arenes, displaying divergent reactivity with respect to that of previously reported electrides.

## Results and Discussion

### Synthesis and Characterization

**Scheme 1:**
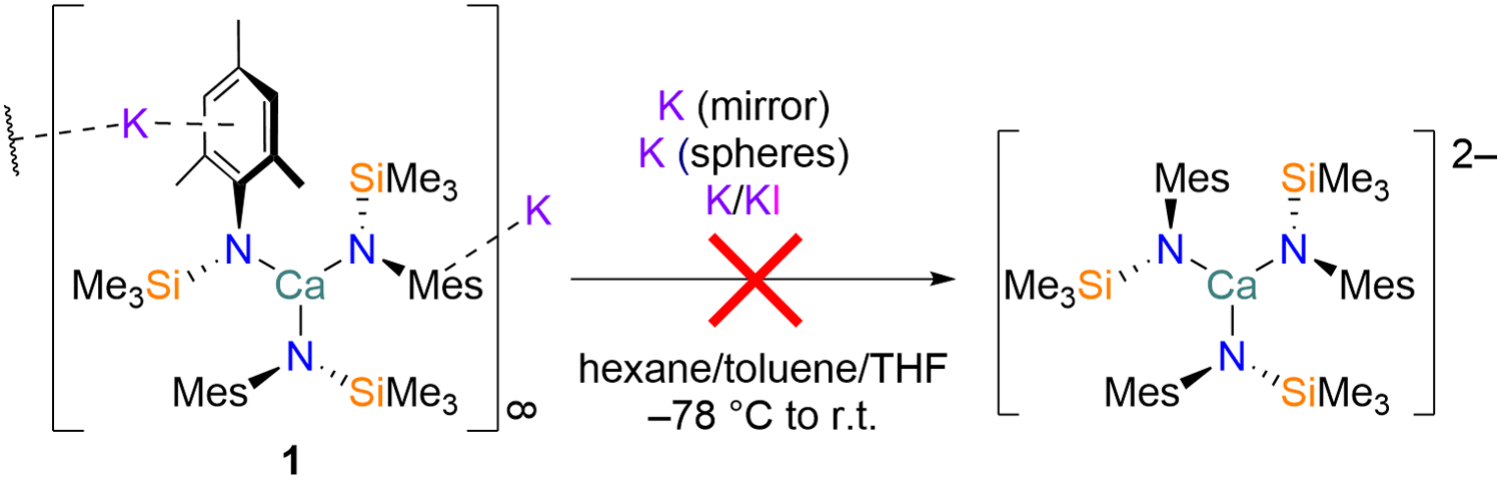
Attempted Reduction of **1** Using Conventional Solution Methods^17^ Note: dashed lines in the molecular representation **1** denote the propagation of a coordination polymer through K···aryl η^6^-interactions.

**Scheme 2:**
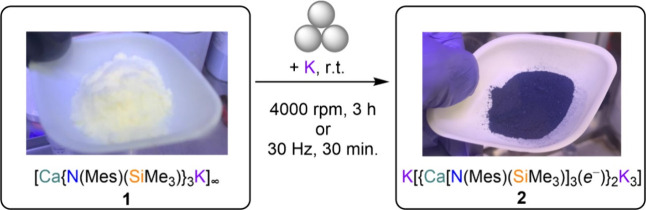
Synthesis of **2** from Mechanochemical Reduction of **1**

The neutral tris(amide) complex [Ca{N(Mes)(SiMe_3_)}_3_K] (**1**; Mes = 2,4,6-trimethylphenyl) was prepared according to previously reported methods.^17^ In previous work, we attempted to convert **1** into a putative monovalent Ca(I) complex, [Ca{N(Mes)(SiMe_3_)}_3_]^2–^, using standard solution methods. However, these attempts did not produce the desired species and only starting materials or decomposition products were isolated from these reactions (Scheme 1).^17^ Degradation pathways promoted by the use of solvents are a common occurrence in low-oxidation state electropositive metals;^22,36−38^ therefore, we decided to explore the reductive chemistry of **1** under solvent-free mechanochemical conditions. A bright blue/purple powder was obtained when **1** was milled with one equivalent of potassium metal, which displayed essentially no solubility in aliphatic hydrocarbons and we formally assigned the product as K[{Ca[N(Mes)(SiMe_3_)]_3_(*e*^*–*^)}_2_K_3_] (**2**, Scheme 2; *vide infra* for structural assignment). **2** was obtained either using a high-energy or a low-energy mixer mill with a longer reaction time (see Experimental Details).^39^

**Figure 2:**
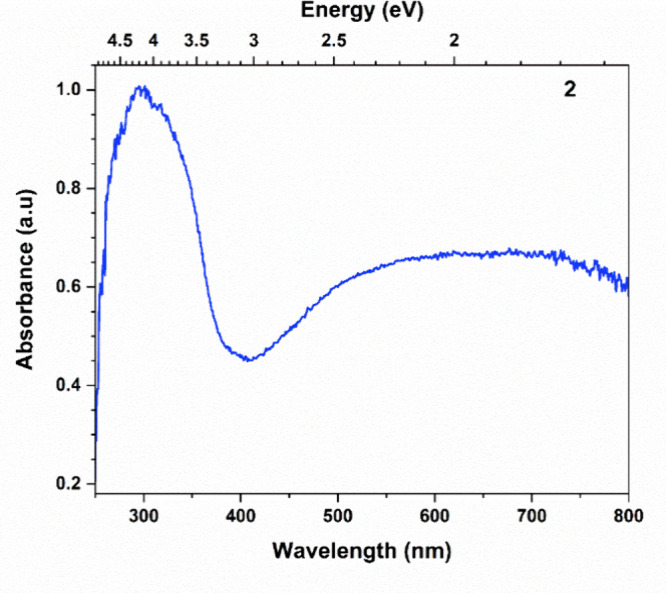
UV–vis absorption spectrum of **2** determined via total reflectance against a reference blank (see SI), recorded between 250 and 800 nm.

Attempts to characterize **2** in solution via NMR spectroscopy were thwarted by the insolubility of the material and high reactivity, leading to either decomposition products or activation of the solvent (*vide infra*). Similarly, attempts to obtain crystalline material suitable for single crystal X-ray diffraction studies were unsuccessful, and so were attempts to crystallize the material via sublimation which led to decomposition of **2** and formation of metallic potassium. Attempts to characterize the products obtained from ball-milling operations via powder X-ray diffraction (PXRD) revealed a largely amorphous substance, together with phases arising from traces of elemental potassium and starting material **1** (Figures S12–13); these phases are more intense when ball milling of starting materials is stopped before completion (Figure S14). Using excess potassium in the reaction led to complete consumption of **1** and formation of an amorphous material with traces of elemental potassium (**2′**, Figure S12 – *vide infra* and Supporting Information (SI) for magnetic characterization). To gain further insight into the electronic structure of **2**, we performed a solid-state UV–vis total reflectance measurement (Figure 2). The resulting absorption spectrum exhibits a strong peak between 340 and 470 nm (λ_max_ = 407 nm, *E* = 3.0 eV) and a broad feature between 450 and 800 nm (*E* = 3.0–1.5 eV). Matsuishi et al. previously reported the optical reflectance spectra of [Ca_24_Al_28_O_68_]^4+^[4e^–^], showing two broad reflection peaks at 0.4 and 2.4 eV respectively, which have been assigned to inter- and intracage transitions of encaged electrons.^40^

**Figure 3:**
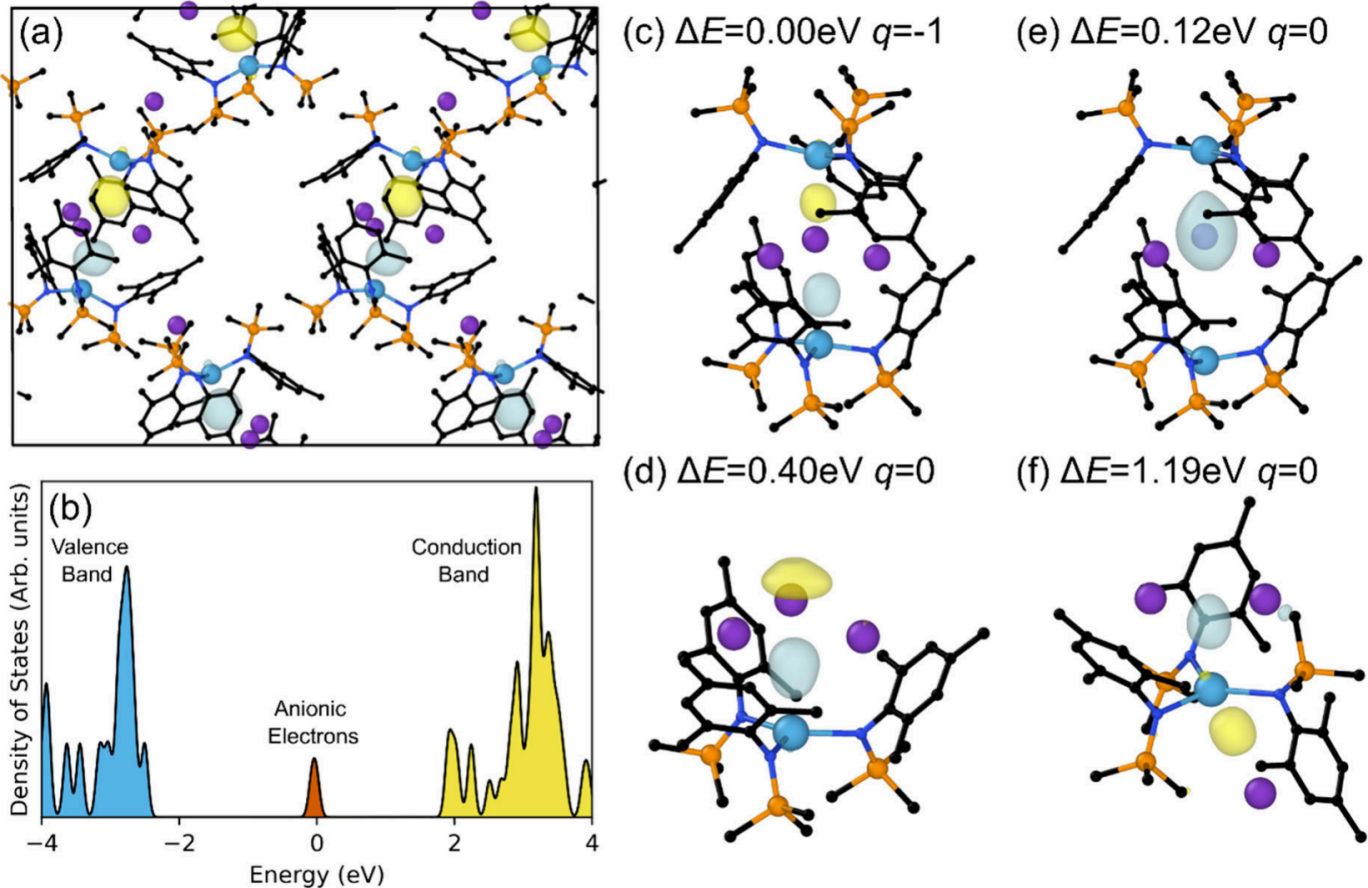
(a) Lowest-energy model for **2**; legend: carbon (black), nitrogen (blue), silicon (orange), potassium (purple), calcium (aquamarine) – light-blue and yellow isosurfaces are the up and down spin anionic e^–^, respectively. (b) Density of states of the model, where blue states are the valence band, yellow states are the conduction band, and orange states correspond to the anionic electrons. (c) A dimer containing two anionic e^–^ – note: this is the local structure of the lowest energy model shown in (a). (d) A broken dimer still containing two anionic e^–^. (e) A dimer containing only a single anionic e^–^. (f) A distorted broken dimer containing two anionic e^–^. Δ*E* is the relative difference in energy for a given structure compared with the “perfect” dimer in (c) and q is the net charge of the complex.

In order to determine low-energy candidate structures, *ab initio* random structure searching (AIRSS) was used.^41^ The geometries of several hundred random structures were optimized without spin, which was found to be sufficient in previous models of electrides.^19^ A selection of low-energy structures was then optimized again with spin included. The lowest-energy candidate structure for **2** found has a formula of K[{Ca[N(Mes)(SiMe_3_)]_3_(*e*^*–*^)}_2_K_3_]. The Ca-electride complex in this structure is very similar to the Ca-hydride complex in **3** (*vide infra*), but with an anionic *e*^*–*^ in the place of H^–^ (Figures 3a,c), and with K^+^ ions intercalated between the complexes. Electrides and hydrides are closely related and it is known that *e*^*–*^ can be substituted with H^–^.^42−44^

In the lowest-energy structure, the two anionic *e*^*–*^ of each dimer are in a singlet state (*S* = 0), indicating that they are antiferromagnetically (AF) coupled. The triplet state (*S* = 1), consistent with ferromagnetic coupling, is higher in energy by 0.38 eV. Given the large distances (around 10 Å between the centers of adjacent dimers), magnetic coupling between anionic *e*^*–*^ belonging to different dimers is expected to be small. The density of states of the lowest-energy structure shows a gap of around 4.2 eV (295 nm) between the valence band (shaded blue in Figure 3b) and the conduction band (shaded yello in Figure 3b). The anionic *e*^*–*^ are strongly localized within the dimers; the associated states appear as a sharp peak in the density of states (shaded orange in Figure 3b) so photoexcitation of anionic *e*^*–*^ into the conduction band of around 1.8 eV (688 nm), would fall somewhere in the broad absorbance peak around 500 to 700 nm (Figure 2). The relatively wide 1.8 eV energy gap between anionic *e*^*–*^ and the conduction band is not consistent with a good conductor. This suggests **2** would be an insulator, which agrees with electroconductivity measurements, which show no conductivity. This is in contrast to what was reported for K^+^[Li(N(SiMe_3_)_2_)]*e*^*–*^, which exhibits a conductivity of 4 S m^–1^.^19^ Interestingly, when the same electroconductivity measurements are performed on **2′** (*i*.*e*., same material prepared using excess potassium), the conductivity is measured at 0.19 S m^–1^ (see SI).

**Figure 4:**
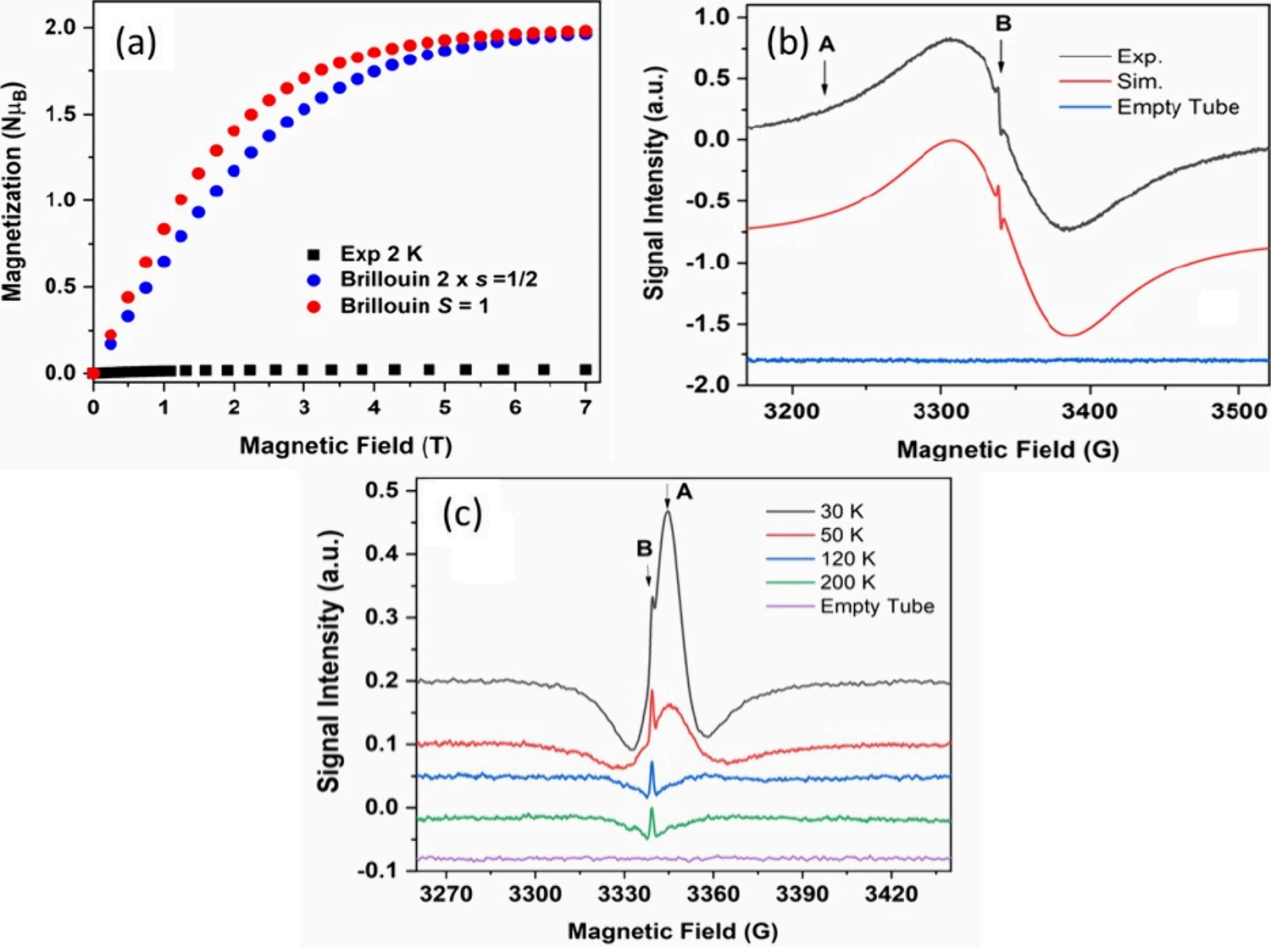
(a) Isothermal magnetization at 2 K for **2** (black) along with the calculated Brillouin curves for two noninteracting electron spins (blue) and ferromagnetically coupled electron spins (red); (b) First harmonic X-band EPR spectrum at 200 K for **2**: experimental (black) and simulation (red); (c) Second harmonic EPR spectra for **2** at 30, 50, 120, and 200 K.

The dimeric structure containing two anionic electrons in a singlet (*S* = 0) ground state was found to be lowest in energy out of many different geometries considered (Figure 3), and because of a significant energy separation (ca. 0.38 eV) from the excited *S* = 1 state, a diamagnetic behavior should be expected for this compound below 300 K. To probe this hypothesis, we performed magnetic susceptibility measurements as a function of temperature (2–300 K) (Figure S28) as well as isothermal magnetization measurements as a function of magnetic field (0–7 T) at 2 K, and both confirm a predominant singlet (*S* = 0) ground spin state (Figure 4 (a)). However, variable temperature EPR spectroscopic studies on **2** have revealed that a small fraction of **2** (ca. 0.03% per unit molar mass of **2**) is paramagnetically active throughout the temperature range 5–280 K (Figures 4 (b), Figure 4 (c), and Figures S29–S33). Close inspection of the first harmonic (FH) (Figures 4 (b), S29 and S30) and second harmonic (SH) (Figures 4 (c) and S31–S33) EPR spectra have revealed the presence of unpaired electrons in two different environments, marked as **A** and **B**. Unpaired electrons associated with sites **B** give a narrow EPR signal (∼1.7 G line width) near the free electron *g* value of 2.0023, whose intensity changes very little with the temperature (Figures 4 (c), S34 and S35). Such a signal has been previously associated with electrides, given the trapped electron in anionic sites interact only weakly with their surroundings.^45^ In contrast, the unpaired electrons in environment **A** give a much broader and slightly unsymmetrical EPR signal at *ca*. *g* = 1.998, whose intensity and line width varies strongly with the temperature (i.e., peak-to-peak FH line width increases from 7.4 G at 5 K to 103 G at room temperature, while the signal intensity becomes smaller as the temperature is raised) (Figure S29). Simulation of the EPR spectrum at 200 K using EasySpin,^46^ provided a rhombic *g*-matrix of *g*_*x,y,z*_ = [1.9958, 1.9962, 2.0026] (Figure 4 (b)). This anisotropy of the *g*-matrix, together with the negative *g*-shift compared to the free electron *g*-value, and the pronounced line broadening could suggest that the electrons trapped in sites **A** have higher conductivity and ability to interact with the K cations from their surroundings. Analysis of the second harmonic (SH) spectra of **2** found a **B**:**A** molar ratio of 0.059 at 50 K (Figure S33), indicating that the vast majority (ca. 94%, but corresponding to only 0.03% of K[{Ca[N(Mes)(SiMe_3_)]_3_(*e*^*–*^)}_2_K_3_] - **2**) of trapped electrons are in sites **A**. To establish if any interaction occurs between electrons, we fit the temperature dependence of the EPR signal intensity (obtained by double integration of the measured FH signal) for **2** using the Bleaney–Bowers equation (see the Supporting Information, Figures S34 and S35), and found an antiferromagnetic coupling constant of ca. –300 K (negative sign denoting AF coupling), which is much smaller than the calculated singlet–triplet separation of −4409 K (for dimer **2**).

To probe this further, we performed additional EPR studies on **2′** (*i.e*. same material obtained with excess potassium) which show the same environments **A** and **B** (Figures S36–S38), with the signal for the latter increased in intensity compared to **2**, and exhibiting hyperfine splitting arising from the interaction with two neighboring K cations. This indicates that the measured EPR signals are potentially associated with other structural defects. To investigate the origin of these trapped spins, we performed additional DFT/AIRSS calculations, considering other defective complexes. A broken dimer configuration (Figure 3d) can be formed with a modest energy cost of 0.40 eV, but it is most favorable for this broken dimer to retain both anionic *e*^*–*^ in a *S* = 0 state with the *S* = 1 being 0.53 eV higher in energy. It is, however, possible to form an “electron vacancy” in the perfect dimer (Figure 3e) with the remaining anionic *e*^*–*^ being shared across both sides of the dimer, with a small preference for one of the two Ca centers. This configuration is only 0.12 eV higher in energy than the perfect dimer and is in an *S* = 1/2 state. We also find another structure corresponding to a highly distorted broken dimer (Figure 3f) which has a higher–but still accessible–formation energy of 1.19 eV. A *S* = 0 is still favored in this configuration, but the *S* = 1 state is only higher in energy by less than 1 meV. The variety of different defective geometries with small formation energies sugeests that ground state electronic structure is an admixture of different formal configurations / wavefunctions nature of this system, which could also account for the amorphous nature of the resulting sample as shown *via* XRD studies (*vide supra*).

### Reactivity studies

**Scheme 3:**
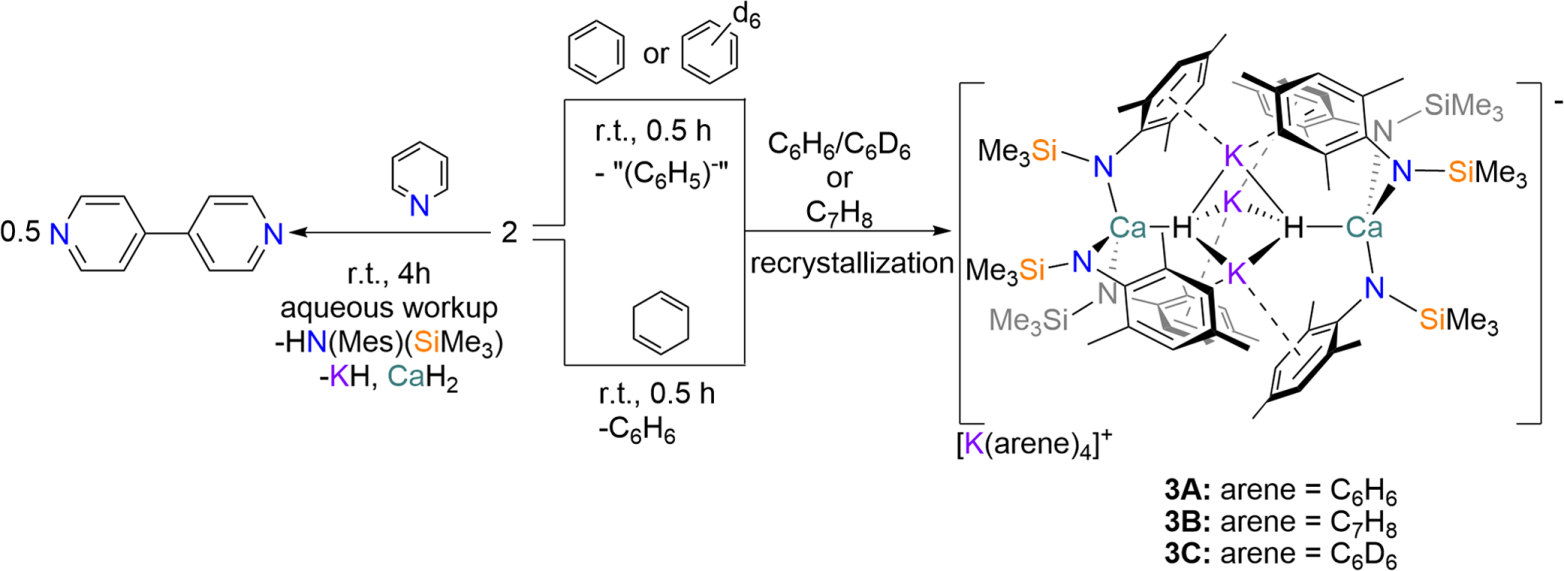
Reactivity of **2** with Pyridine, Benzene and 1,4-Cyclohexadiene, and Formation of Ca Hydride Complexes **3A**, **3B** and **3C**

**Figure 5:**
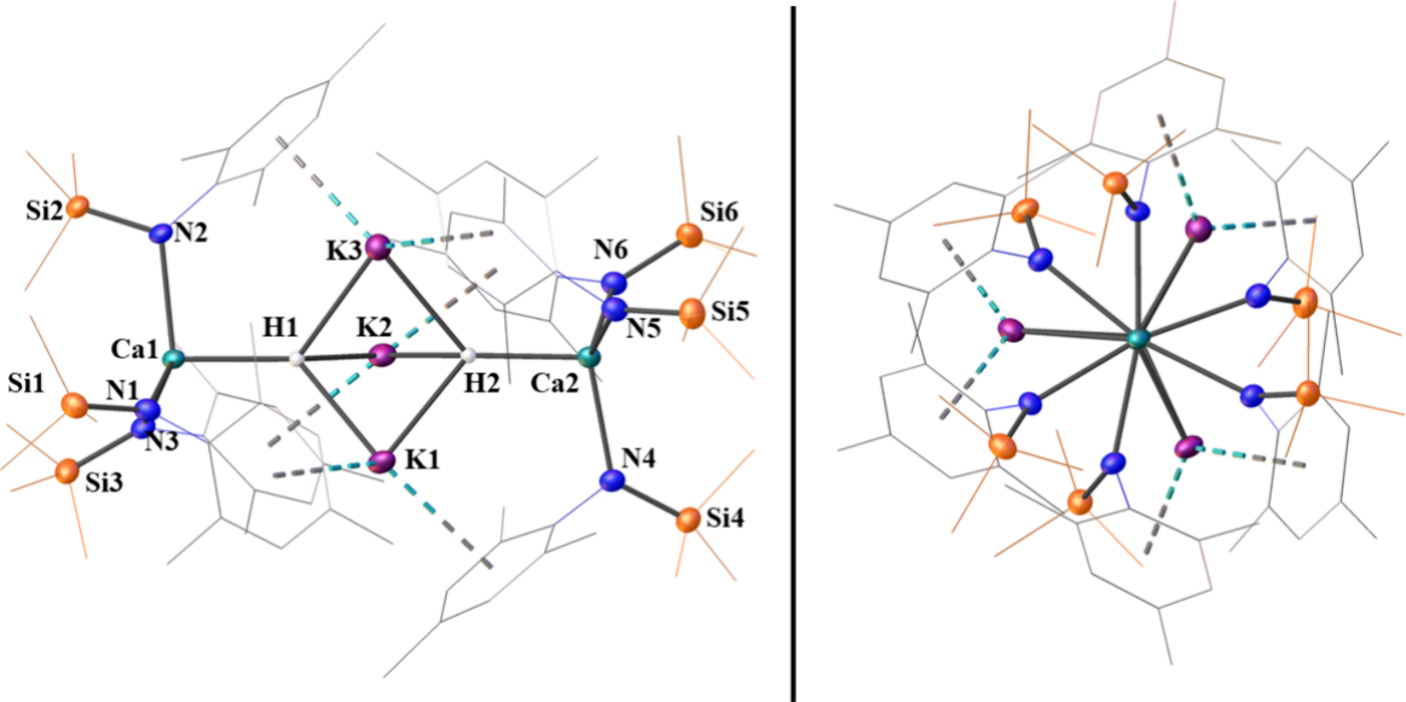
Crystal structure of **3A**; side-on (left), perpendicular to K_3_ plane (right). Sequestered [K(C_6_H_6_)_4_]^+^ cation and all hydrogen atoms except hydride ligands have been omitted for clarity. Ellipsoids of all other atoms are set at 50% probability level. Legend: nitrogen (blue), silicon (orange), hydrogen (white), potassium (purple), calcium (aquamarine). Mesityl and methyl groups are displayed as wireframes.

Reactivity of **2** was probed first by reaction with pyridine (Scheme 3). This resulted in the instantaneous formation of a bright orange solution followed by subsequent darkening of the mixture to give a black solution and a dark precipitate. The mixture was quenched with propan-2-ol and followed by standard aqueous workup; NMR analysis of the resulting organic phase confirmed the formation of 4,4′-bipyridine together with a ligand degradation product (Figure S7). The formation of 4,4′-bipyridine indicates the reductive activation of pyridine and the consequent radical coupling and concomitant C–H activation. Similarly to what was observed for K^+^[Li{N(SiMe_3_)_2_}]e^–^, the byproduct of the reaction is likely to be either CaH_2_ or KH.^19^ When a sample of **2** was treated with benzene, instantaneous formation of a gray mixture was observed, followed by the gradual formation of a clear orange solution. Slow evaporation of the solvent led to the formation of large yellow crystals, which were determined to contain a multimetallic, anionic calcium hydride complex of formula [K(η^6^-C_6_H_6_)_4_][{Ca[N(Mes)(SiMe_3_)](H)}_2_K_3_] (**3A**, Scheme 3 and Figure 5); **3A** is formed together with an aryl-containing species, which we were not able to definitively identify. **3A** was also obtained cleanly from the reaction of **2** with 1,4-cyclohexadiene at room temperature. Recrystallization from toluene, affords the same anionic hydride unit combined with the [K(C_7_H_8_)_4_]^+^ cation (**3B**, Figure S10).

**Scheme 4:**
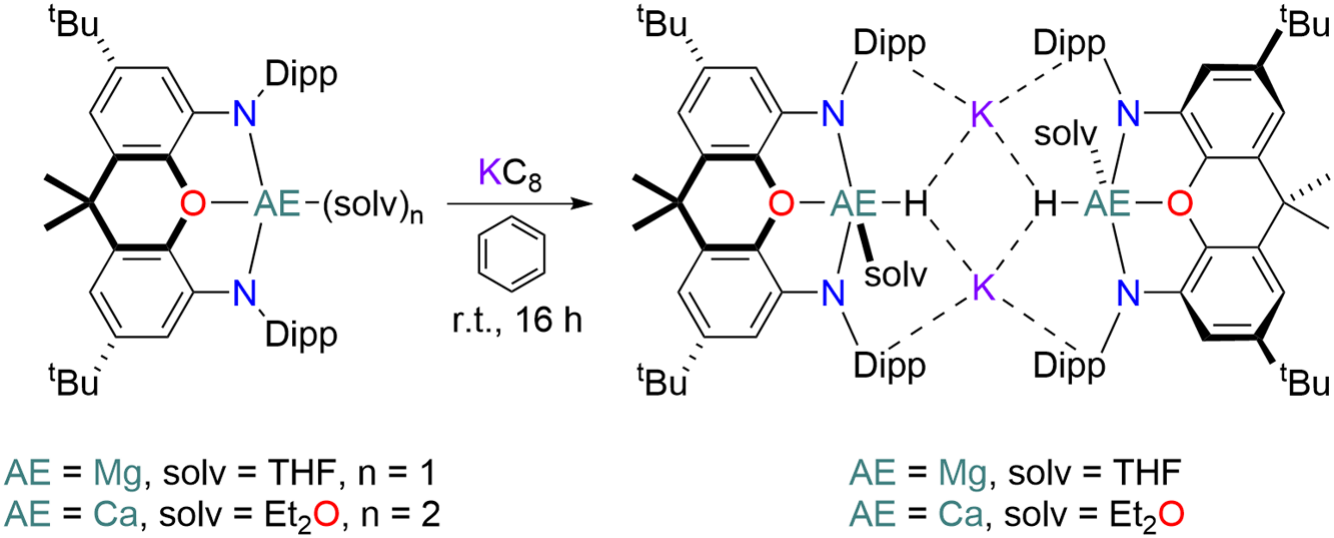
Synthesis of [{AE(NON)(H)(solv)}_2_K_2_] by Hicks and Co-workers^46^

Characterization of **3A** by multinuclear (^1^H, ^13^C{^1^H}, ^29^Si{^1^H}) NMR spectroscopy produces simple spectra indicating a highly symmetrical product from the equivalence of the amide ligands, and that the dimeric structure is retained in solution. The hydride resonance is observed as a singlet at δ_H_ = +2.66 ppm and is significantly further upfield when compared to the hydride resonance of the similar Ca–H species reported by Hicks and co-workers (δ_H_ = +3.63 ppm) and even further shifted from bridging hydride species [{Ca(^Dipp^BDI)(μ-H)}_2_] (Dipp = 2,6-diisopropylphenyl) reported by Wilson et al. (δ_H_ = +4.27 ppm).^47,48^ When we attempted to obtain **3** by reacting **2** with C_6_D_6_, the intensity of the hydride resonance is diminished in the ^1^H NMR spectrum (Figure S4) and a new signal appears in the ^2^H NMR spectrum resonating at +2.73 ppm (Figure S3), thus in agreement with the formation of a deuteride, *i.e*. [K(η^6^-C_6_D_6_)_4_][{Ca[N(Mes)(SiMe_3_)](D)}_2_K_3_] (**3C**). Nonetheless, we cannot unequivocally exclude the possibility of other hydride sources providing a minor contribution to the formation of **3**, such as intra- or intermolecular activation of the ligand, though no other species are detected in the ^2^H NMR spectrum.^49^ In the solid state, compound **3** is stable for several months and is stable in benzene for over 2 weeks at room temperature, but rapidly decomposes upon addition of THF. The formation of **3** is reminiscent of the reduction of [AE(NON)(solv)_n_] (AE = Mg, solv = THF, n = 1; AE = Ca, solv = Et_2_O, n = 2; NON = 4,5-bis(2,6-diisopropylanilido)-2,7-di-*tert*-butyl-9,9-dimethylxanthene) in arenes, which affords [{AE(NON)(H)(solv)}_2_K_2_] (AE = Mg, solv = THF; AE = Ca, solv = Et_2_O) (Scheme 4).^47^ Parallels could also be drawn with the activation of benzene by the aluminyl anions in[{Al(^Dipp^BDI)}_2_K_2_]^50^ and [{Al(NON)}_2_K_2_].^51^ Attempts to prepare compound **3** by direct reaction of **1** with KH were unsuccessful. Noteworthily, we previously reported that reaction of **1** with K in benzene does not produce any reduction products nor hydride complex **3**,^17^ thus highlighting the unique reactivity profile of **2**. Additionally, no molecular hydride species were observed from the reaction of K^+^[Li{N(SiMe_3_)_2_}]e^–^ with benzene, leading instead to the formation of biphenyl together with LiH as byproduct.^19^ Several examples of benzene and arene reductions have been reported involving heavy AEs^10^ and divalent lanthanides.^52,53^ Harder and co-workers proposed a mechanism for AE-mediated (AE = Ca, Sr) benzene reduction involving initial coordination of benzene and subsequent formation of a hydride-containing intermediate, which then disproportionates prior to reductive C–C coupling and formation of biphenyl (though it is noteworthy their computational analyses indicate an alternative pathway going *via* H_2_ elimination as the most plausible mechanism).^10^ We have attempted to model the formation of **3** computationally, however, we could not identify a pathway that accounts for the coordination of benzene to Ca (see ESI for further details). When using the anionic unit derived from **1** as a starting point, [Ca{N(Mes)(SiMe_3_)}_3_]^−^, attempts to model benzene association to Ca saw an increase in free energy to +4.9 kcal mol^–1^, with the benzene far from the Ca center (**I′**; Ca···H_bnz_ = 5.96 Å – see SI). No Wheland intermediate was successfully isolated, and therefore we conclude that the Ca center does not facilitate the C–H activation of the benzene (or pyridine). This suggests that the reactivity of **2** is quite distinct from putative low oxidation state AE(I) compounds previously reported, and more akin to “electride-like” reactivity where the metal centers and ligands play a templating role (*vide infra*).

Interestingly, complex **3** is not the only product formed from the reaction of **2** and benzene. The ^1^H NMR spectrum of the crude reaction solution indicates the presence of **3** and another species determined to be a C–H activation product of the calcium tris(amide) starting material, [Ca{N(Mes)(SiMe_3_)}_2_{C_6_H_2_(NSi(Me)_2_CH_2_)(Me)_2_}] (**4**, see ESI). Control experiments revealed that the formation of **4** is not related to the activation of benzene by **2**; its formation is associated with the degradation of residual starting material **1** present in the reaction, and computational analysis shows that this is triggered by the deprotonation of one of the *ortho*-methyl substituents on the aryl ring of the N(Mes)(SiMe_3_) ligand (see ESI for computational analysis). The presence of traces of starting material **1** was confirmed via PXRD characterization (see ESI). Additionally, PXRD analyses revealed that less of starting material **1** is present in the Ca RoSE **2** when prepared using excess potassium metal (*i.e*. product **2′** is formed instead), and gratifyingly we observed a significant reduction in the formation of **4** when **2′** is reacted with benzene.

### Characterization of **3** and Computational Studies

The solid-state structure of **3A** features two hydride ligands bound to calcium centers, with the complex being stabilized via potassium-aryl interactions from the mesityl substituents of the amide ligands (Figure 5). **3A** shares parallels with a similar Ca–H species [{Ca(NON)(H)(Et_2_O)}_2_K_2_] reported by Hicks and co-workers which also features hydride ligands and is supported by potassium-aryl interactions.^47^ The discussion of hydride bond lengths and angles in **3A** is possible only to a limited extent due to the nature of XRD studies, nonetheless the hydrides were clearly identified from the residual electron density map and their positions refined freely. The two Ca–H bonds are 2.26(3) Å (Ca1–H1) and 2.31(3) Å (Ca2–H2) in length with each hydride ligand interacting with a further three potassium cations [H···K 2.72(2)- 2.85(2) Å]. The calcium centers adopt a heavily distorted tetrahedral geometry with an average N–Ca–N angle of 116.6(7)° and an average H–Ca–N angle of 100.8(2)°. The calcium atoms lie out of the {NNN} plane by 0.433(2) Å (Ca1) and 0.460(2) Å (Ca2), therefore, the geometry around the Ca atoms can be viewed as puckered trigonal planar capped by a hydride ligand (τ^_4_^ = 0.57).^54^ Each potassium atom in the anionic unit is encapsulated by two mesityl groups which have their normal to the ring oriented inward toward the center of the structure. Data quality from XRD studies of **3B** was very poor and metric parameters could not be compared to those of **3A**. Nonetheless, connectivity of **3B** is clear-cut and confirmed the formation of an analogous species.

**Figure 6:**
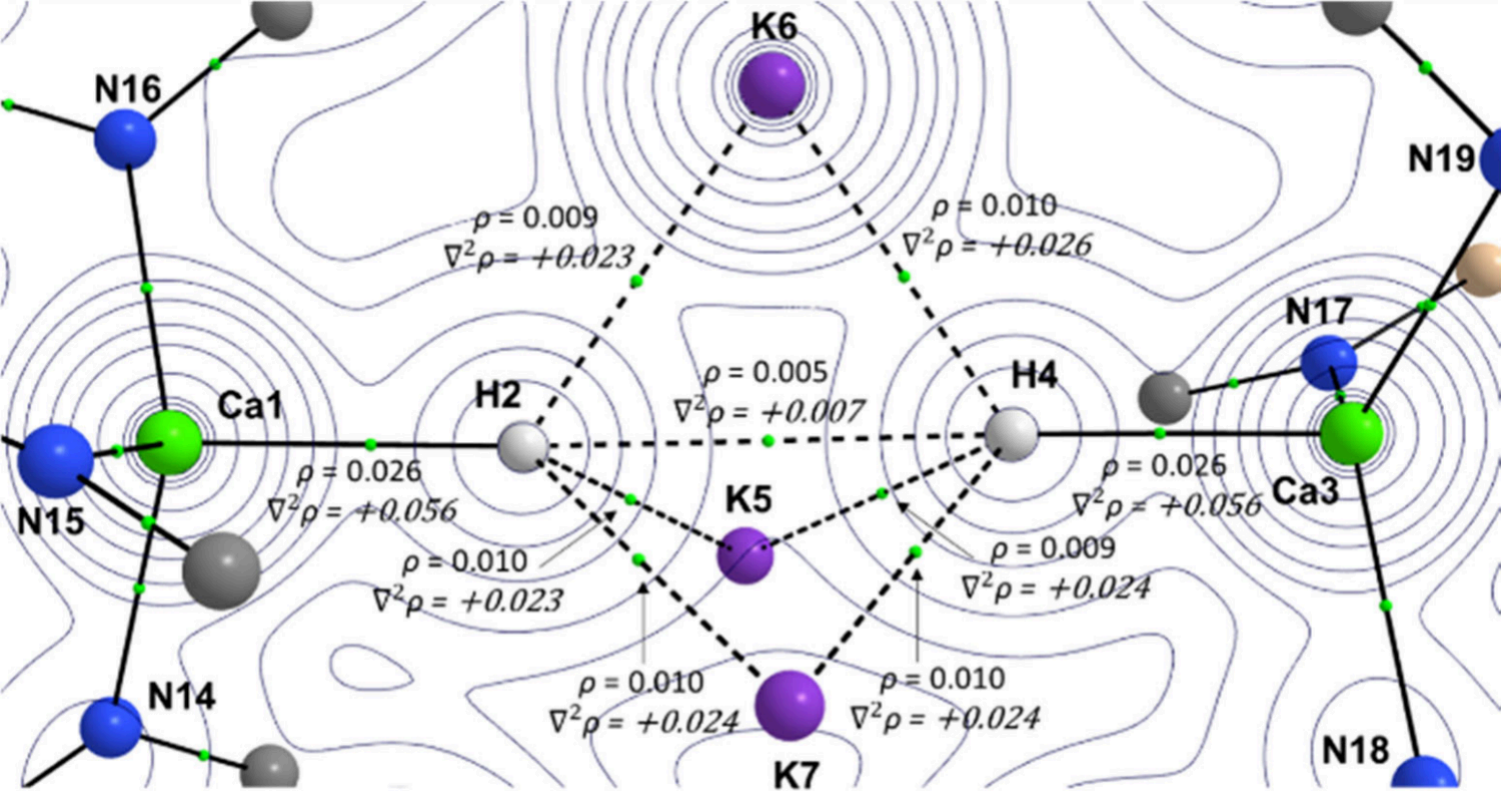
QTAIM molecular graph of the BP86/BS1-optimized geometry of the anion of **3A**, focused on the core (Ca, H and K) atoms. The electron density (ρ) and Laplacian values (∇^2^ρ, italic) are shown for bond critical points (BCPs; small green spheres along bond vectors). The electron density contours are computed in the {Ca–H} planes.

Quantum Theory of Atoms in Molecules (QTAIM) and Natural Bond Orbital (NBO) electronic structure analyses were carried out on the anionic component of **3A**, which was optimized using Density Functional Theory (DFT); BP86/BS2//BP86/BS1 (see ESI for full methodology details). Wiberg Bond Indices (WBIs) highlight that there is no discernible electron sharing between the two Ca centers, although a marginal amount is observed for each hydride with their respective Ca center (WBI(Ca–H) = 0.1369, 0.1389). These values alongside the QTAIM data (Figure 6), where all bond critical points between Ca–H, H–K and H–H atoms have positive Laplacian values, strongly support ionic bonding within the core of the [CaH{K_3_}HCa]^−^ unit. Bader atomic charges (see Figure S21) of q_Ca_ = +1.5, q_H_ = −0.7 and q_K_ = +0.8, are in line with a trimeric {K_3_}^3+^ core sandwiched by two anionic Ca(II)-hydride complexes. No appreciable NNAs (Non-Nuclear Attractors) were observed in the QTAIM data.

We hypothesize that the bulky amide ligands help to stabilize the discrete hydride complex **3**, and prevent it from disproportionation to form CaH_2_. Distinct conformations of the {N(Mes)(SiMe_3_)}^−^ ligands were analyzed of the anionic unit derived from **1**, [Ca{N(Mes)(SiMe_3_)}_3_]^−^, to assess the role of the ligands, or more specifically the orientation of the ligand’s mesityl groups in relation to each other.^17^ What is clear from the conformers computed and looking at the rotation barrier for a ligand at Ca, is that the preferred ligand orientation has all three mesityl groups from each [Ca{N(Mes)(SiMe_3_)}_3_]^−^ subunit, on the same face of the Ca complex, even without the templating afforded by the three potassium cations. The {K_3_}^3+^ triangle strongly interacts with the π systems of the mesityl groups, with each K^+^ interacting with one mesityl group from each of the Ca complexes, which holds the two Ca-hydride complexes close together in **3** (free energy stabilities are given in Table S4 alongside the dihedral values for the mesityl group; see SI for dihedral details, τ = N_b_–Ca–N–C_ipso_).

## Conclusion

In this work we have shown that the mechanochemical reduction of a tris-amide calcium complex affords a new Ca-RoSE, K[{Ca[N(Mes)(SiMe_3_)]_3_(*e*^*–*^)}_2_K_3_] (**2**), which could be obtained with both low- and high-energy milling equipment. This new Ca-RoSE does not display any electroconductivity, which is consistent with the calculated structure in which the anionic *e*^*–*^ are strongly localized and situated 1.8 eV below the conduction band. The lowest energy structure of **2** exhibits a *S* = 0 ground state with the two *e*^*–*^ being strongly antiferromagnetically coupled, thus suggesting a diamagnetic behavior. This nonmagnetic ground state is confirmed by SQUID and EPR data. Nonetheless, full EPR characterization of **2** reveals the presence of a very small amount (0.03 mol %) of paramagnetic components, which we attribute to trapped electrons that interact only weakly with their surroundings, and to dimers containing only one anionic *e*^–^. **2** reacts readily with pyridine to give C–C reductive coupling products (4,4′-bipyridine), while reactivity with benzene produces a calcium hydride complex (**3**), obtained via direct C–H activation. While analogous reactivity with pyridine has been already achieved with other electrides, direct hydride formation has not been previously observed. Crucially, the formation of this rare Ca-hydride complex could not be replicated with standard “wet” synthetic methods, thus highlighting the unique reactivity of **2**. Our computational analyses further revealed structural details which are essential to the new Ca-RoSE, and we propose that our ligand design and resulting conformational flexibility play a key role in the divergent reactivity observed with respect to other electrides^19^ and AE(I) synthons.^10^ This work paves the way to a new generation of reagents that can be accessed through very simple solid-state mechanochemical synthesis, which can be employed in C–C forming reactions and to deliver facile C–H activation chemistry.

## Experimental Section

### General Methods

THF and toluene were passed through columns containing molecular sieves, then stored over a potassium mirror (toluene), or over 4 Å molecular sieves (THF) and thoroughly degassed prior to use. Hexane and diethyl ether were purchased anhydrous from Tokyo Chemical Industry, dried over activated molecular sieves for 7 days, then stored over a potassium mirror. For NMR spectroscopy, C_6_D_6_ and C_4_D_8_O were dried by refluxing over potassium, and then vacuum transferred and degassed by three freeze–pump–thaw cycles before use. NMR spectra were recorded on either a Bruker Avance III HD 400 or Bruker Avance III 500 spectrometer operating at 400.07/500.13 (^1^H), 61.4/76.8 (^2^H), 100.60/125.78 (^13^C{^1^H}) or 79.48/99.36 (^29^Si{^1^H}) MHz. To achieve a greater signal-to-noise ratio, the ^29^Si{^1^H} NMR spectra were acquired with a DEPT24 pulse sequence. NMR spectra were recorded at 298 K unless otherwise stated and were referenced to residual solvent signals in the case of ^1^H and ^13^C{^1^H} experiments, or externally referenced to SiMe_4_ (^29^Si{^1^H}). FTIR spectra were recorded on a Bruker Alpha II spectrometer with Platinum-ATR module. Magnetic measurements were performed using a Quantum Design MPMS3 superconducting quantum interference device (SQUID) magnetometer operating at 1.8–400 K under applied fields up to 7 T; experimental details are in S7.1. The electron paramagnetic resonance (EPR) spectra were recorded in continuous-wave (C.W.) mode, using a Bruker EMX Plus Spectrometer operating at X-band (9.36 GHz) and variable temperatures (5 to 293 K); experimental details are in S7.2. Mass spectrometry were carried out by Dr Sharad Mistry at the University of Leicester, using a Waters Acquity XEVO Q ToF spectrometer (electrospray). Elemental microanalyses were carried out by the London Metropolitan University Elemental Analysis service. Anhydrous CaI_2_ was purchased from Alfa Aesar and baked at 200 °C for 4 h prior to use. HN(Mes)(SiMe_3_),^55^ K[N(Mes)(SiMe_3_)]^56^ and **1**(17) were prepared according to literature procedures. All glassware used to store or to investigate the reactivity of **2** and **2′** was first treated with trimethylsilyl chloride to remove trace hydroxide residues, rinsed with deionized water and acetone, and dried at 150 °C for 16 h. Solid state reactions were performed using either: (i) an IKA ULTRA-TURRAX Tube Drive Disperser; 20 mL polypropylene milling jar, 4 × 3 mm stainless steel ball-bearings (316 stainless steel, 0.5 g), or (ii) a Retch MM400 mixer mill; 25 mL stainless steel milling jar, 1 × 10 mm stainless steel ball-bearing (316 stainless steel, 13.5 g).

#### Synthesis of K^+^[{Ca{N(Mes)(SiMe_3_)}_3_K]*e*^–^ (**2**)

##### Method 1

**1** (0.350 g, 0.5 mmol, 1 equiv), freshly cut potassium metal (19.6 mg, 0.5 mmol, 1 equiv) and 4 stainless steel ball-bearings (3 mm diameter) were added to a 25 mL polypropylene milling jar inside an argon-filled glovebox before being milled at 4000 rpm for 3 h. Upon completion, the resultant blue powder (0.360 g, quantitative yield) was decanted and stored in silylated glassware.

##### Method 2

**1** (2.457 g, 3.524 mmol, 1 equiv), freshly cut potassium metal (0.138 g, 3.524 mmol 1 equiv) and 1 stainless steel ball-bearing (10 mm diameter) were added to a 25 mL stainless steel milling jar inside an argon-filled glovebox before being milled at 30 Hz for 30 min. Upon completion, the resultant blue powder (0.360 g, quantitative yield) was decanted and stored in silylated glassware.

##### **2′**

**1** (0.500 g, 0.717 mmol, 1 equiv), freshly cut potassium metal (58.6 mg, 1.5 mmol, 2.1 equiv) and 1 stainless steel ball-bearing (10 mm diameter) were added to a 25 mL stainless steel milling jar inside an argon-filled glovebox before being milled at 30 Hz for 30 min. Upon completion, the resultant blue powder (0.550 g, quantitative yield) was decanted and stored in silylated glassware.

#### Synthesis of [K(η^6^-C_6_H_6_)_4_][{Ca[N(Mes)(SiMe_3_)](H)}_2_K_3_] (**3A**) and [K(η^6^-C_7_H_8_)_4_][{Ca[N(Mes)(SiMe_3_)](H)}_2_K_3_] (**3B**)

##### Method 1

Benzene (2 mL) was added to a freshly prepared sample of **2** (0.201 g, 0.27 mmol) and stirred at room temperature, yielding an instantaneous formation of a black mixture which lightened to form an orange solution. Slow evaporation of this solution yielded **3A** as large, yellow crystals overnight (0.060 g, 0.03 mmol, 24.9%).

##### Method 2

1,4-Cyclohexadiene (10 mL) was added to a freshly prepared sample of **2** (0.500 g, 0.68 mmol) and stirred at room temperature for 16 h. The resulting yellow suspension was filtered yielding **3A** as a yellow solid (0.166 g, 0.11 mmol, 16.5%). Crystallization from toluene afforded **3B**, where benzene molecules binding the potassium counterion are replaced by toluene.

^1^H NMR (500 MHz, 298 K, C_6_D_6_): δ/ppm = 0.55 (s, 54H, Si(C*H*_3_)_3_), 2.07 (s, 18H, *para*–C*H*_3_), 2.29 (s, 36H, *ortho*–C*H*_3_), 2.66 (s, 2H, Ca-*H*), 6.66 (s, 12H, Ar-*H*).

^13^C{^1^H} (125 MHz, 298 K, C_6_D_6_): δ/ppm = 4.88 (Si(*C*H_3_)_3_), 20.39 (*para*-*C*H_3_), 22.30 (*ortho*-*C*H_3_), 123.48 (Ar-*C*), 128.89 (Ar-*C*), 134.78 (Ar-*C*), 159.20 (Ar-*C*).

^29^Si{H} (80 MHz, 298 K, C_6_D_6_) δ/ppm = −13.4.

#### Synthesis of [K(η^6^-C_6_D_6_)_4_][{Ca[N(Mes)(SiMe_3_)](D)}_2_K_3_] (**3C**)

Benzene-d_6_ (0.5 mL) was added to a freshly prepared sample of **2** (0.017 g, 0.023 mmol) in a J. Young’s NMR tube yielding an instantaneous formation of a dark brown mixture. The reaction mixture was frozen using liquid nitrogen and the solvent removed by sublimation *in vacuo* yielding a light brown powder, which was fully dried under reduced pressure. Benzene-H_6_ (0.5 mL) was added and the ^2^H NMR spectrum obtained. ^2^H{^1^H} NMR (61 MHz, C_6_H_6_, 298 K): δ/ppm = 2.73 (s, Ca-*H*).

## Supporting Information Available

Additional experimental details and spectroscopic data (multinuclear NMR, IR, UV–vis, EPR), crystallographic data (single crystal XRD, powder XRD) for all compounds, with additional computational details for compounds **2** (AIRSS) and **3** (DFT). Additional research data supporting this publication are available from FigShare at http://dx/doi.org/10.25392/leicester.data.26105326. (PDF)
