# Supplementary material for: Mechanochemical Synthesis, Characterization and Reactivity of a Room Temperature Stable Calcium Electride
Source: J Am Chem Soc. 2024 Oct 8;146(42):28914–24. doi: 10.1021/jacs.4c09408 (PMC11503782; doi:10.1021/jacs.4c09408)
Supplement: Supplementary file 1 — ja4c09408_si_001.pdf [file ja4c09408_si_001.pdf]

Mechanochemical synthesis, characterization and reactivity of a room temperature stable calcium electride

Alex W. J. Bowles,[a§] James A. Quirk,[b§] Yu Liu,[a] George H. Morritt,[c] Marina Freitag,[b] George F. S. Whitehead,[d] Adam W. Woodward,[d] Adam Brookfield,[e] Conrad A. P. Goodwin,[d] David Collison,[e] Floriana Tuna,[e*] Claire L. McMullin,[f*] James A. Dawson,[b*] Erli Lu,[g*] and Fabrizio Ortu[a*]

[a]School of Chemistry, University of Leicester, University Road, Leicester, LE1 7RH, UK

[b]Chemistry – School of Natural and Environmental Sciences, Newcastle University, Newcastle upon Tyne, NE1 7RU, UK

[c]School of Mathematics, Statistics, and Physics, Newcastle University, Newcastle upon Tyne, NE1 7RU, UK

[d]Department of Chemistry, The University of Manchester, Manchester, M13 9PL, UK

[e]Department of Chemistry and Photon Science Institute, The University of Manchester, Manchester, M13 9PL, UK

[f]Department of Chemistry, University of Bath, Claverton Down, Bath, BA2 7AY, UK

[g]School of Chemistry, University of Birmingham, Edgbaston, Birmingham, B15 2TT, UK

[§]These authors contributed equally

*email: floriana.tuna@manchester.ac.uk, c.mcmullin@bath.ac.uk, James.Dawson@newcastle.ac.uk, e.lu@birmingham.ac.uk, fabrizio.ortu@leicester.ac.uk

Contents

S1. Additional experimental details S2

S2. NMR and IR data S4

S3. Crystallographic data S9

S4. UV-vis measurements S16

S5. Electroconductivity measurements S18

S6. Computational details S19

S7. EPR and SQUID measurements S53

S8. References S61

S1. Additional experimental details

Reaction of 2 with pyridine

A Schlenk flask was charged with a glass-coated magnetic stirrer bar and **2** (0.25 g, 0.3395 mmol) before pyridine (10 mL) was added at room temperature. The reaction mixture was stirred overnight before propan-2-ol (1 mL) was added. All volatile components were removed in vacuo before dichloromethane (25 mL) was used to extract the organic components. Removal of the solvent under reduced pressure yielded an oily residue confirmed to be 4,4′-bipyridine and ligand degradation products by ^{1}H NMR spectroscopy (Figure S5) and mass spectrometry.

Synthesis of [Ca{N(Mes)(SiMe$_3$)}$_2${C$_6$H$_2$(NSi(Me)$_2$CH$_2$)(Me)$_2$}] (4)

During the isolation of Ca hydride complex **3**, we identified complex [Ca{N(Mes)(SiMe$_3$)}$_2${C$_6$H$_2$(NSi(Me)$_2$CH$_2$)(Me)$_2$}] (**4**), which formed as a result of degradation of one of the supporting amide ligands. Control experiments revealed that the formation of **4** is not related to the activation of benzene by **2**, but it is associated with the degradation of residual starting material **1** present in the reaction, as confirmed *via* PXRD characterization (see Section S3). PXRD analysis also revealed that less of the starting material **1** is present in the electride material **2** when prepared using excess potassium metal (**2′**), and gratifyingly we observed a significant reduction in the formation of **4** when **2′** is reacted with benzene. Complex **4** was prepared directly by heating a toluene solution of **1** to 70 °C for 3 hours (Scheme S1); colourless crystals were obtained from storing this solution at −30 °C. Complex **4** was fully characterised by multinuclear NMR spectroscopy, XRD studies, FT-IR spectroscopy and elemental analysis. The ^{1}H NMR spectrum clearly shows activation of one NMes ligand and the consequential loss of symmetry with additional resonances compared to that of **1** (Figure S3). Most telling of this is the formation of two additional singlets at 6.42 ppm and 6.93 ppm each integrating to one H-atom. These resonances correspond to the two aryl-protons of the C–H activated ligand, which are now inequivalent due to loss of symmetry.

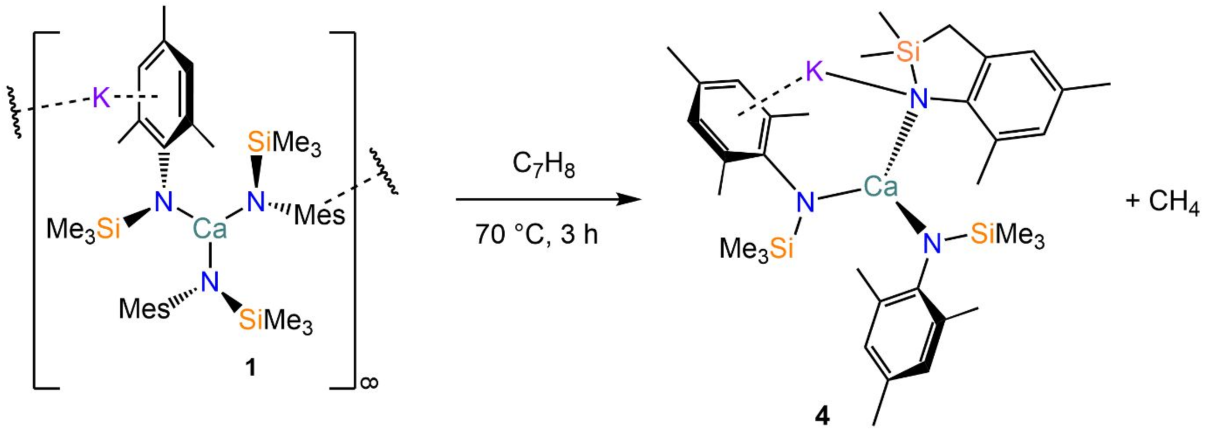

Scheme S1: Formation of decomposition product **4**.

A Schlenk flask was charged with a magnetic stirrer bar and **1** (0.25 g, 0.358 mmol) before toluene (25 mL) was added at room temperature. The resulting pale yellow solution was heated to 70 °C for 3 hours before the volume of solvent was concentrated under reduced pressure and the solution stored at −30 °C yielding **4** as large colourless crystals (0.178 g, 0.261 mmol, 67.9%).

^{1}H NMR (500 MHz, 298 K, C_6D_6): δ/ppm = −0.05 (br s, 6H, Si(C*H*$_3$)$_2$), 0.47 (s, 18H, Si(C*H*$_3$)$_3$), 1.36 (s, 3H, (C*H*$_3$)C), 1.77 (br s, 2H, C*H*$_2$), 2.05 (br s, 6H, *para*-C*H*$_3$), 2.26 (s, 3H, (C*H*$_3$)C), 2.27 (s, 12H, *ortho*-C*H*$_3$), 6.42 (s, 1H, C*H*), 6.63 (br s, 4H, *meta-H*), 6.93 (s, 1H, C*H*). ^{13}C{^{1}H} (125 MHz, 298 K, C_6D_6): δ/ppm = 4.6 (Si(*C*H$_3$)$_2$), 17.9 ((*C*H$_3$)C), 19.6 (*C*H$_2$), 20.7 (*para*-*C*H$_3$), 20.8 ((*C*H$_3$)C), 21.2 (*ortho*-C(*C*H$_3$)), 117.7 (*C*(CH$_3$)), 122.5 (*para-C*) 128.8 ((CH$_3$)*C*), 129.2 (*meta-C*), 129.4 ((CH$_3$)*C*), 133.1 (*C*(CH$_3$)), 134.1 (*ortho*-*C*(CH$_3$)), 158.5 (N(*C*)). No observable signals were obtained from ^{29}Si{^{1}H} NMR experiments. Anal. calcd. for $C_{35}H_{56}N_3KCaSi_3$: C, 61.61 %; H, 8.27 %; N, 6.16 %. Found: C, 61.23 %; H, 7.98 %; N, 6.08 %.

S2. NMR and IR data

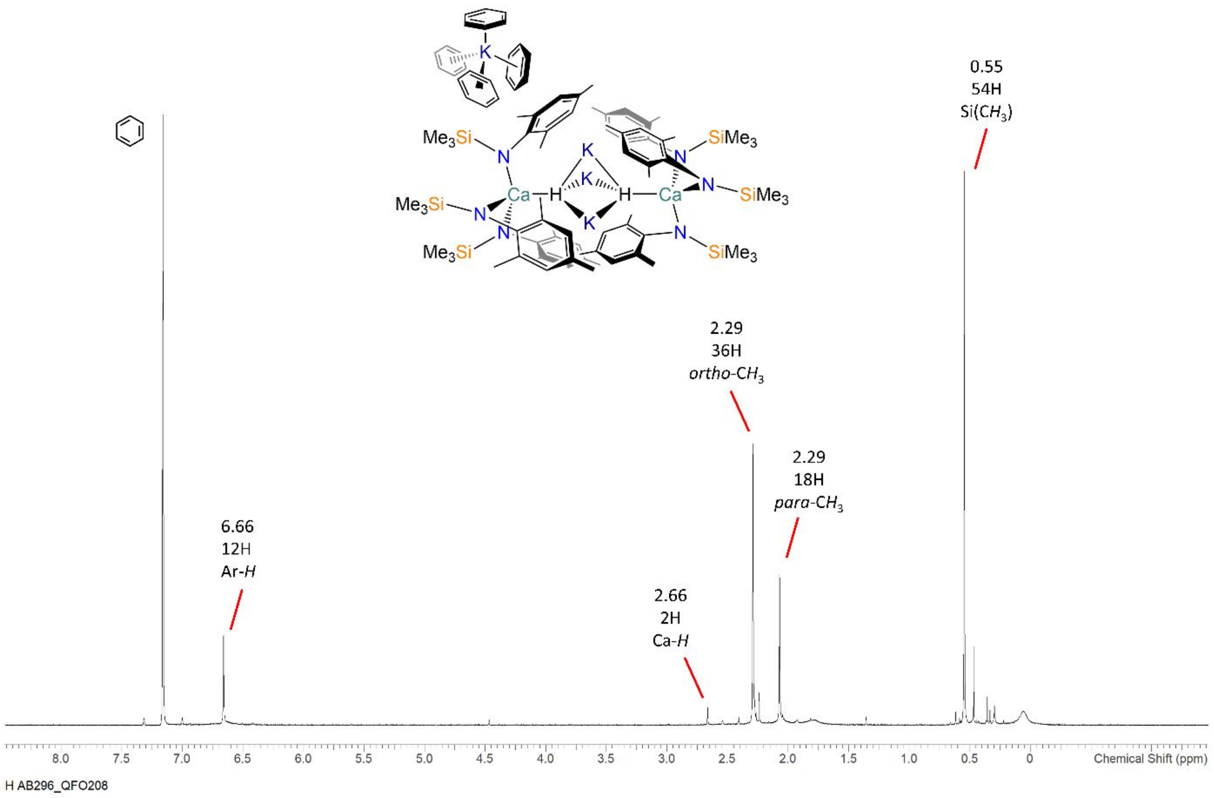

Figure S1: ^{1}H NMR (C_6D_6, 298 K, 400 MHz) spectrum of **3**.

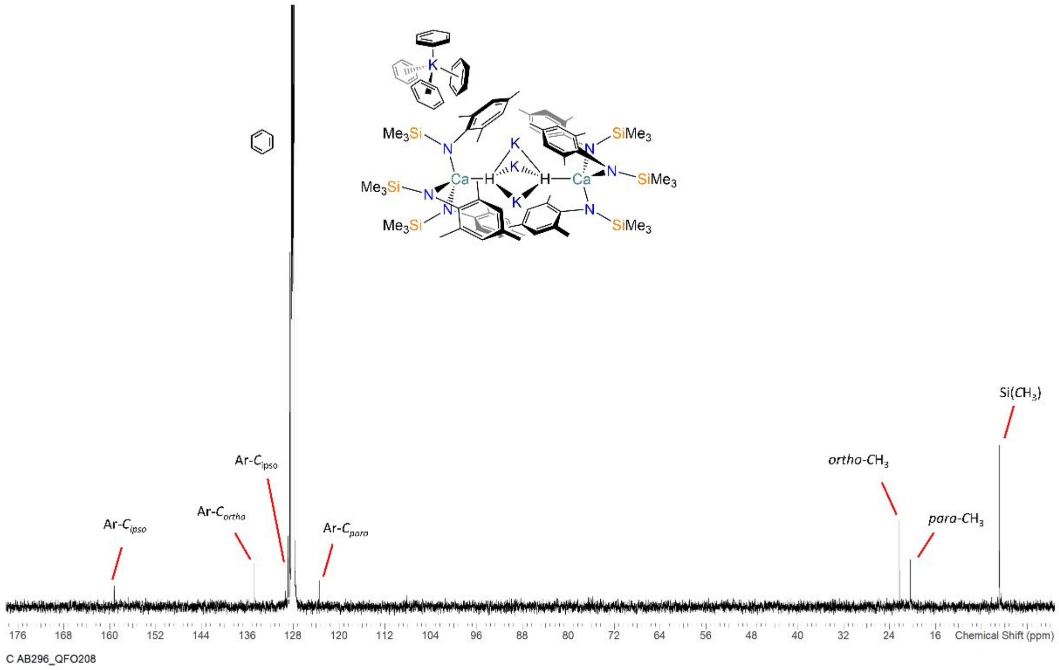

Figure S2: $^{13}C\{^{1}H\}$ NMR (C_6D_6, 298 K, 100 MHz) spectrum of **3**.

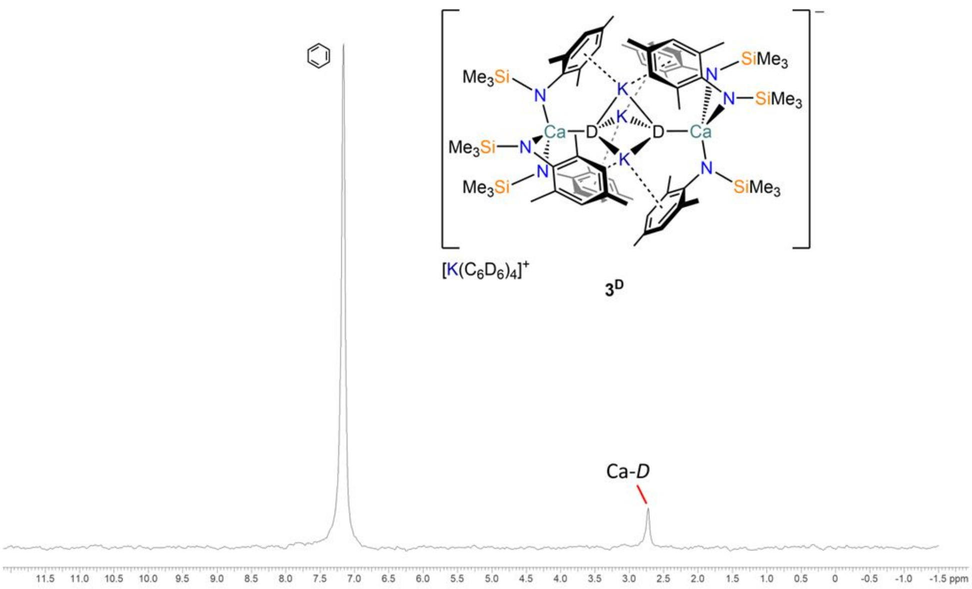

Figure S3: $^{2}H\{^{1}H\}$ NMR (61 MHz, 298 K, C_6D_6) spectrum of $\mathbf{3^D}$.

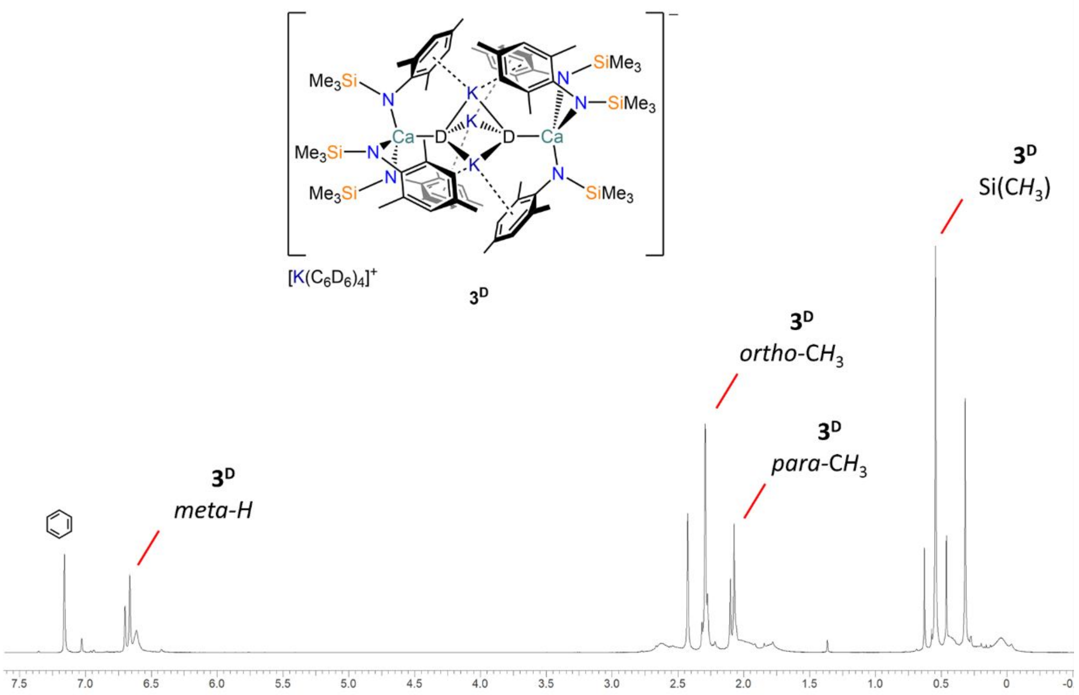

Figure S4: ^{1}H NMR (400 MHz, 298 K, C_6D_6) spectrum of $\mathbf{3^D}$ (crude mixture, no workup).

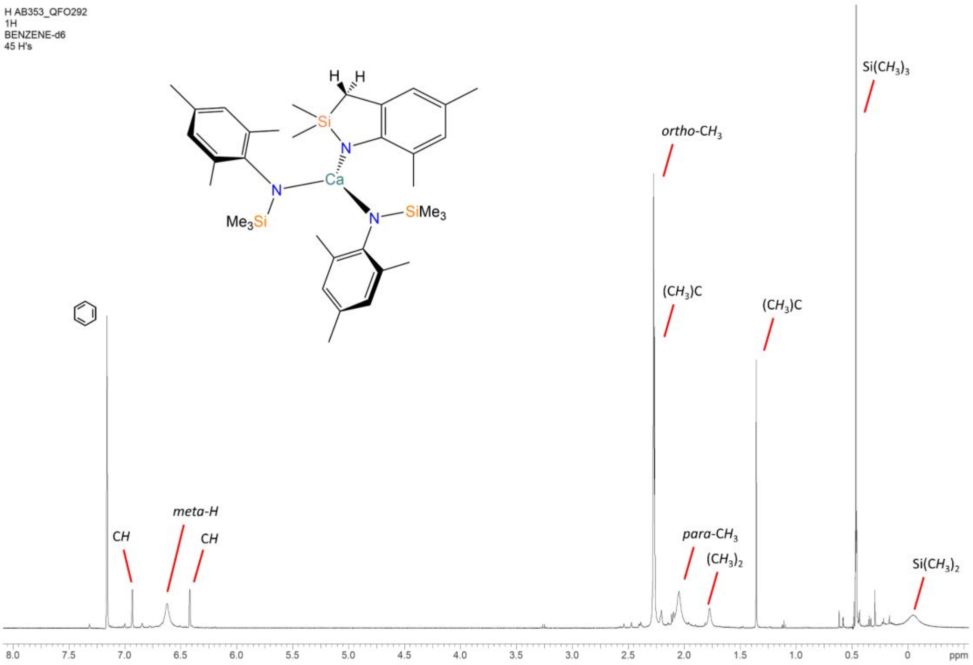

Figure S5: ^{1}H NMR (500 MHz, 298 K, C_6D_6) spectrum of **4**.

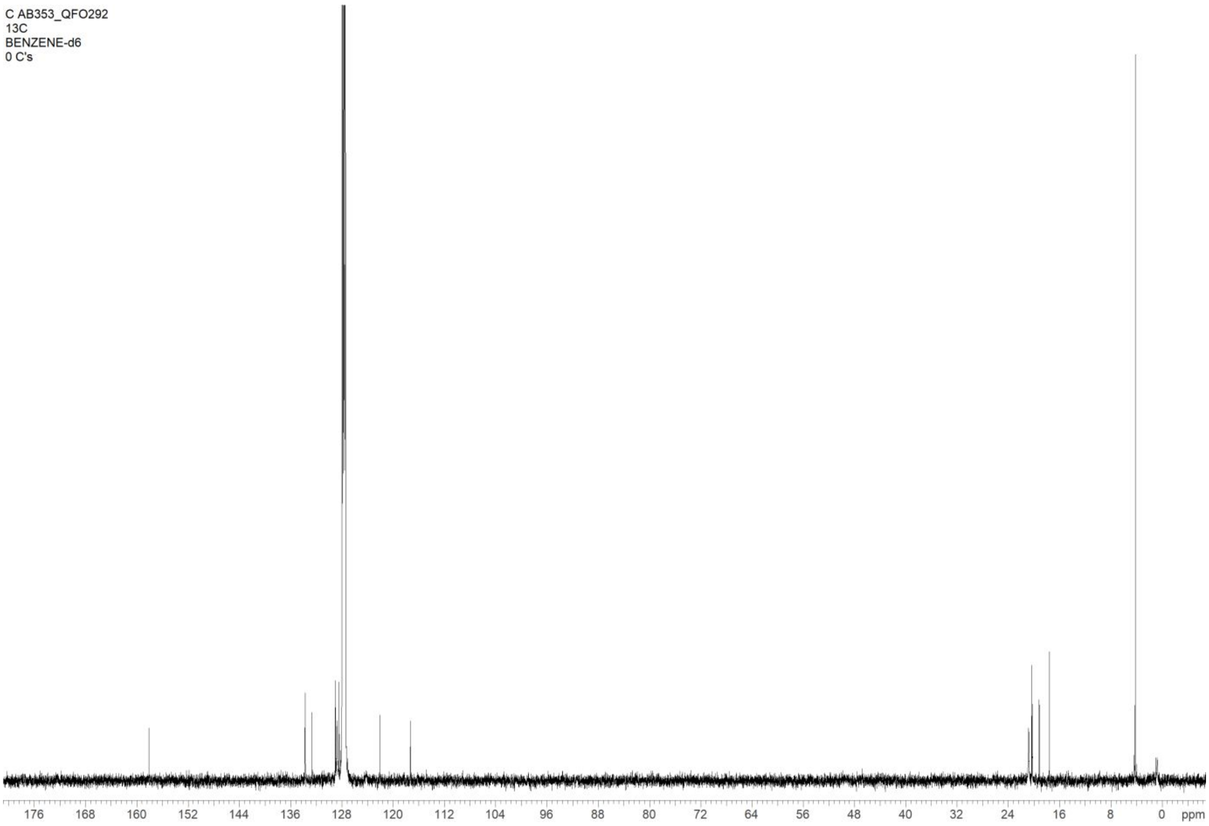

Figure S6: $^{13}C\{^{1}H\}$ NMR (125 MHz, 298 K, C_6D_6) spectrum of **4**.

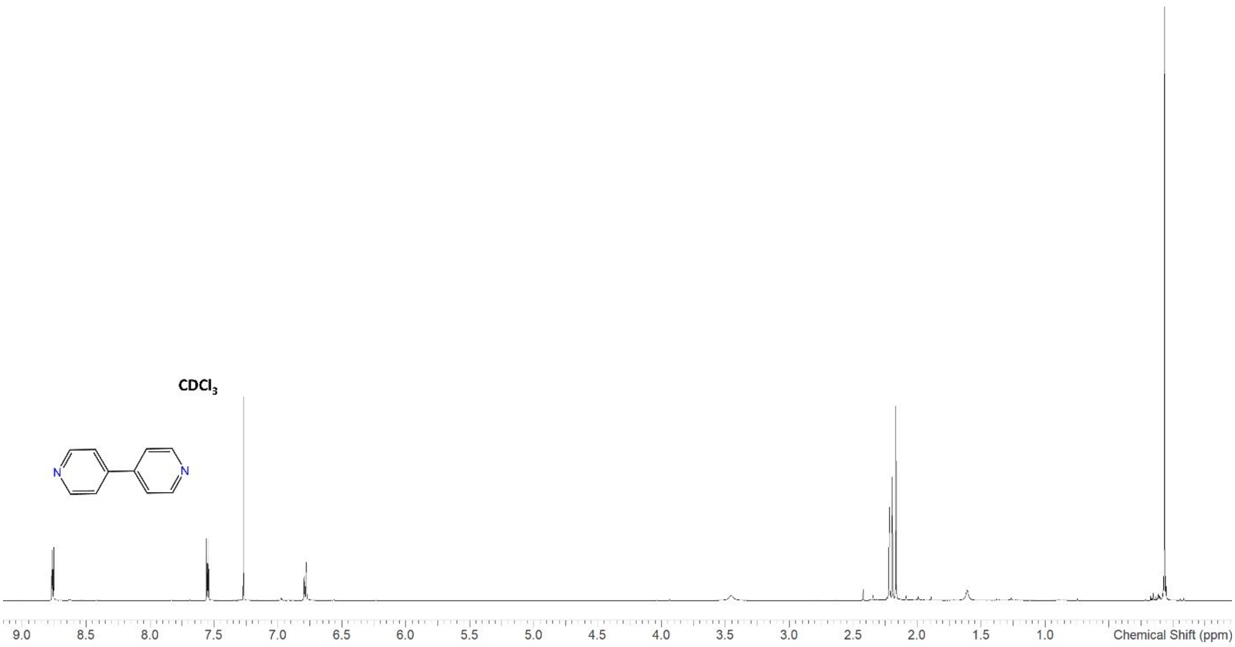

Figure S7: ^{1}H NMR (400 MHz, 298 K, $CDCl_3$) spectrum of crude product obtained from the addition of pyridine to **2** following quenching with propan-2-ol.

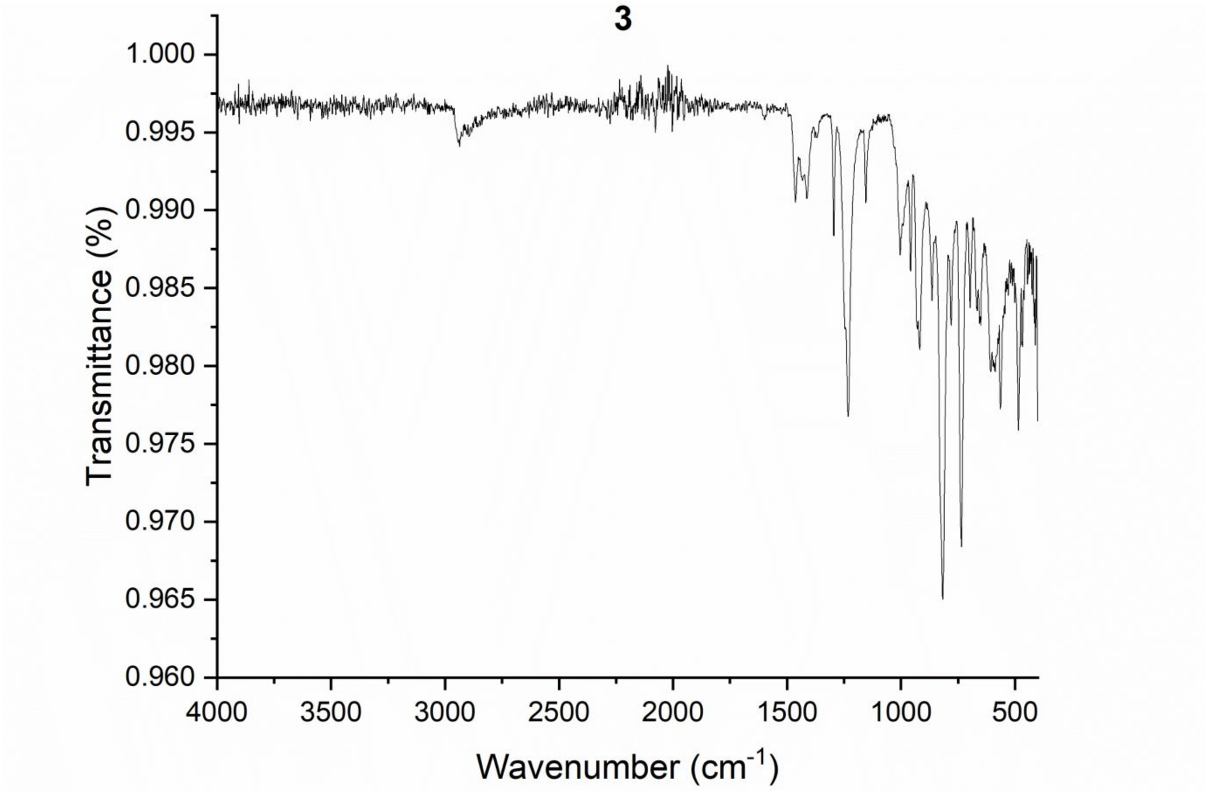

Figure S8: Solid state IR spectrum of **3**.

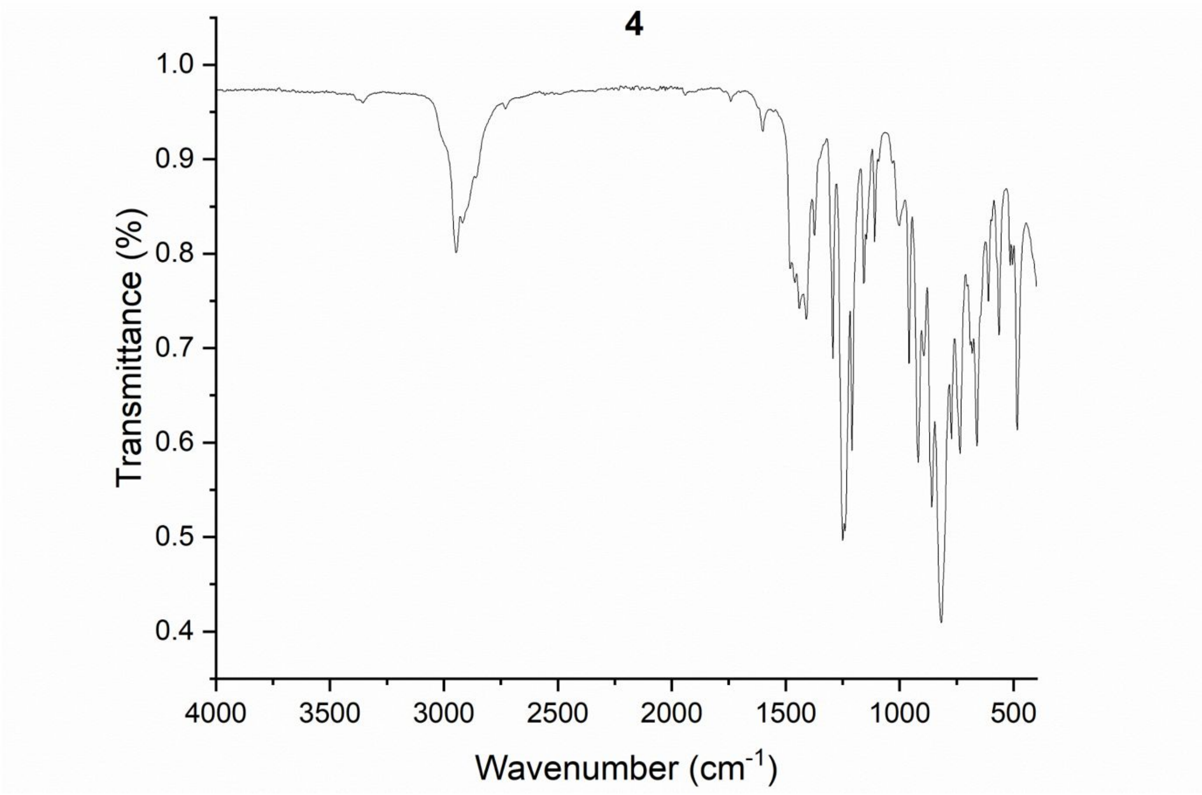

Figure S9: Solid state IR spectrum of **4**.

S3. Crystallographic data

Crystallographic method

Single Crystal XRD. The crystal data for all compounds are compiled in Table S1. Crystals of **3A**, **3B** and **4** were examined using a Bruker D8 Quest diffractometer with a Photon III detector and a microfocus source with Cu-Kα radiation (λ = 1.54178 Å). Intensities were integrated from data recorded on 1° frames by ω or φ rotation. A multi-scan absorption correction method with a beam profile was applied.[1] The structures were solved using SHELXS[2] or SHELXT;[3] the datasets were refined by full-matrix least-squares on reflections with F2≥2σ(F2) values, with anisotropic displacement parameters for all non-hydrogen atoms, and with constrained riding hydrogen geometries;[4] Uiso(H) was set at 1.2 (1.5 for methyl groups) times Ueq of the parent atom. The largest features in final difference syntheses were close to heavy atoms and were of no chemical significance. SHELX[2,3] was employed through OLEX2[4] for structure solution and refinement. The structures have been deposited with the Cambridge Crystallographic Data Centre (CCDC 2346544-2346546). This information can be obtained free of charge from www.ccdc.cam.ac.uk/data_request/cif.

Powder XRD. Microcrystalline samples of **2** were mounted on a goniometer head with a micromount using a minimum amount of Fomblin. X-ray diffraction data were collected at 100 K using a Rigaku FR-X rotating anode single crystal X-ray diffractometer with Cu Kα radiation (λ = 1.5418 Å), a Hypix-6000HE detector and an Oxford Cryosystems nitrogen flow gas system. Data were collected between 3-70 °θ, with a detector distance of 150 mm and a beam divergence of 1.5 mRad. CrysAlisPro was used to measure, reduce and integrate XRD data.[5] Le Bail method profile fitting was performed using JANA2020 software.[6,7] In each case Le Bail method profile fitting was performed using the unit cell parameters for **1** and potassium metal.

Table S1: Crystallographic data for **3A**, **3B** and **4**.

	**3A**	**3B**	**4**
Formula	$C_{102}H_{152}Ca_2K_4N_6Si_6$	$C_{101}H_{157}Ca_2K_4N_6Si_6$	$C_{35}H_{56}CaKN_3Si_3$
Formula Weight	1867.39	1860.42	682.27
Crystal Size, mm	0.356×0.266×0.222	0.01×0.008×0.008	0.346×0.33×0.222
Crystal System	monoclinic	monoclinic	monoclinic
Space group	$P2_1/c$	$C2/c$	$P2_1/c$
a, Å	20.0459(5)	20.148(3)	17.9787(6)
b, Å	19.8009(5)	18.945(3)	12.2698(4)
c, Å	27.7174(7)	33.091(5)	19.6594(9)
α, °	90	90	90
β, °	97.427(2)	100.175(9)	117.1260(10)
γ, °	90	90	90
V, Å^3	10909.5(5)	12432(3)	3859.7(3)
Z	4	4	4
Temperature, K	120(2)	120(2)	120(2)
ρ_{calc}, g cm^{-3}	1.137	0.994	1.174
μ, mm^{-1}	3.239	2.839	3.452
F(*000*)	4024	4020	1472
No. of reflections (unique)	104042 (21410)	111995 (11395)	102125 (8432)
S^a	1.02	1.25	1.03
$R_1(wR_2)$ ($F^2 > 2\sigma(F^2)$)	0.0395 (0.1039)	0.1497 (0.4451)	0.0331 (0.0912)
R_{int}	0.0531	0.430	0.0308
Min./max. diff map, Å^{-3}	–0.26, 0.51	–1.07, 1.54	–0.35, 0.58

^{a}Conventional $R = \Sigma||Fo| - |Fc||/\Sigma|Fo|$; $R\text{w} = [\Sigma w(Fo^2 - Fc^2)^2/\Sigma w(Fo^2)^2]^{1/2}$; $S = [\Sigma w(Fo^2 - Fc^2)^2/\text{no. data} - \text{no. params})]^{1/2}$ for all data.

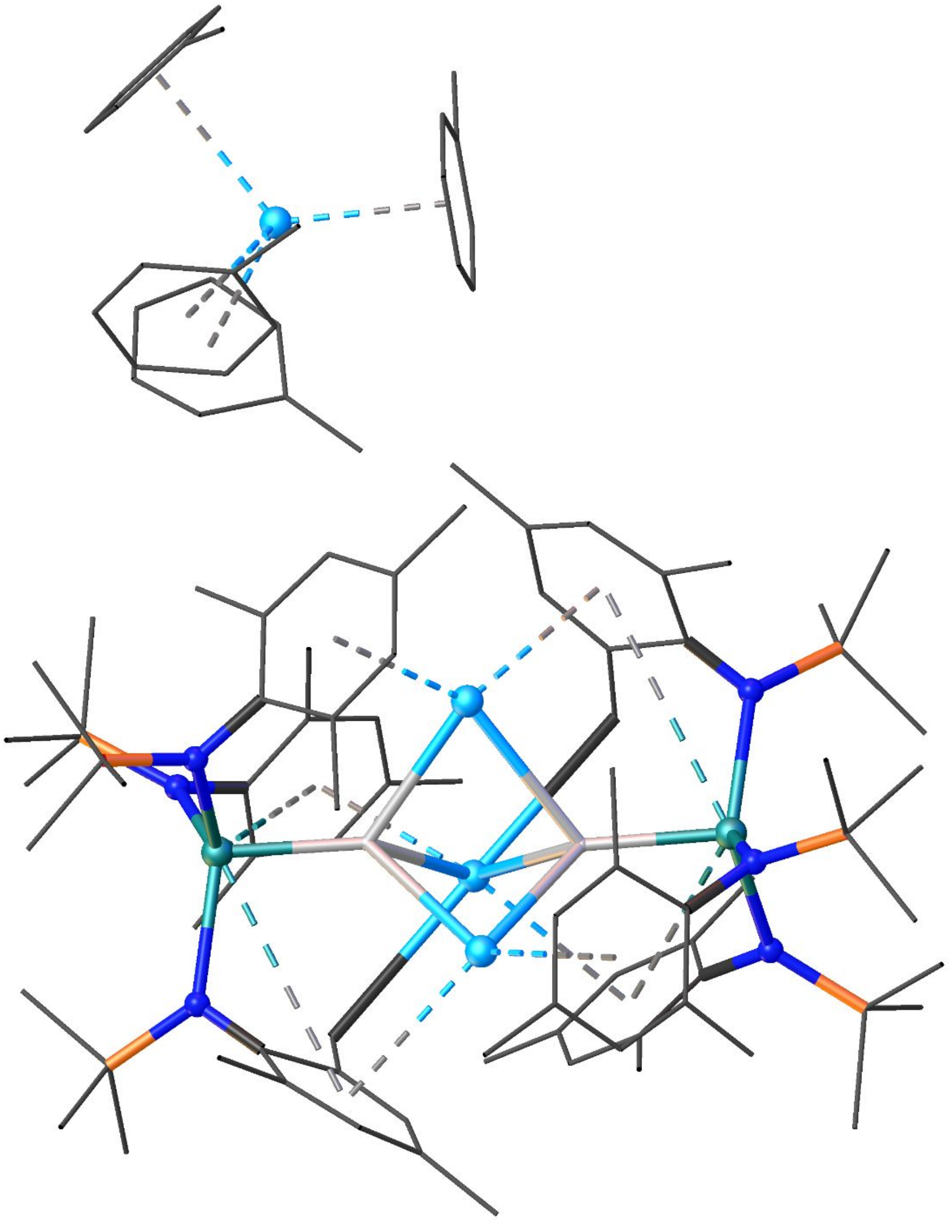

Figure S10: Ball and stick representation of the molecular structure of **3B**. Toluene molecules, together with mesityl and methyl groups are displayed as wireframe for clarity. Hydrogen atoms have also been omitted for clarity with the exception of hydride ligands. Legend: carbon (black), nitrogen (blue), silicon (orange), potassium (light blue), calcium (aquamarine), hydrogen (white).

*Solid state characterisation of **4***

In the solid state, complex **4** displays a polymeric arrangement supported by potassium-aryl interactions akin to those exhibited by **1**. The Ca–N bond distances are also comparable to those measured for the parent tris(amide) **1**. In contrast to **1**, complex **4** features a K–N bond between the potassium cation and the nitrogen atom of the activated ligand. As a result, there is a distortion of the trigonal planar geometry around the calcium centre.

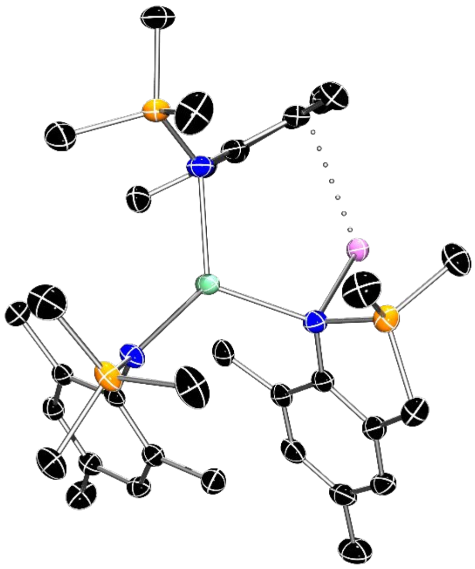

Figure S11: Crystal structure of **4**. Ellipsoids are set at 30% probability level. Hydrogen atoms have been omitted for clarity. Legend: carbon (black), nitrogen (blue), silicon (orange), potassium (pink), calcium (aquamarine).

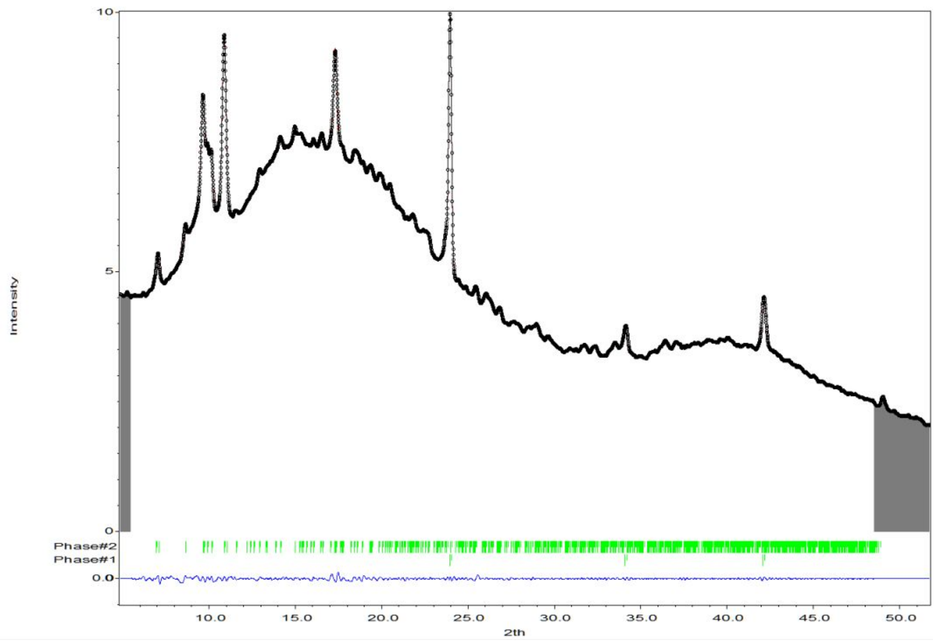

Figure S12: PXRD analysis of **2** obtained using an IKA ULTRA-TURRAX mixer mill within a 25 mL PTFE jar and four 3 mm ball bearings (4000 rpm, 3 hours). Black = measured observed data, Red = fitted profile, Green = expected peak positions tick marks, Blue = Observed - fitted difference, Grey regions are regions of the full pattern excluded from the Le Bail method profile fitting.

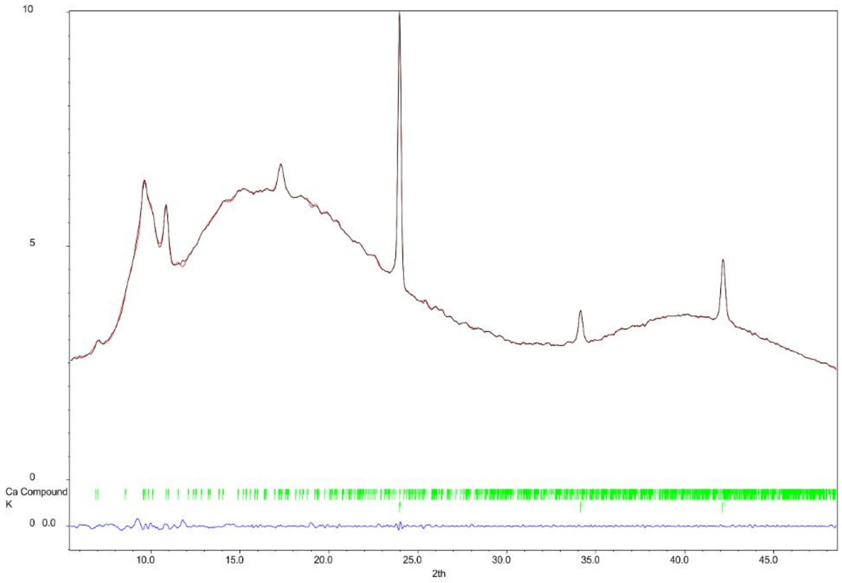

Figure S13: PXRD analysis of **2** obtained using a Retsch™ MM400 mixer mill within a 25 mL Retsch™ stainless steel jar and a 10 mm diameter stainless steel ball (30 Hz, 30 minutes). Black = measured observed data, Red = fitted profile, Green = expected peak positions tick marks, Blue = Observed - fitted difference

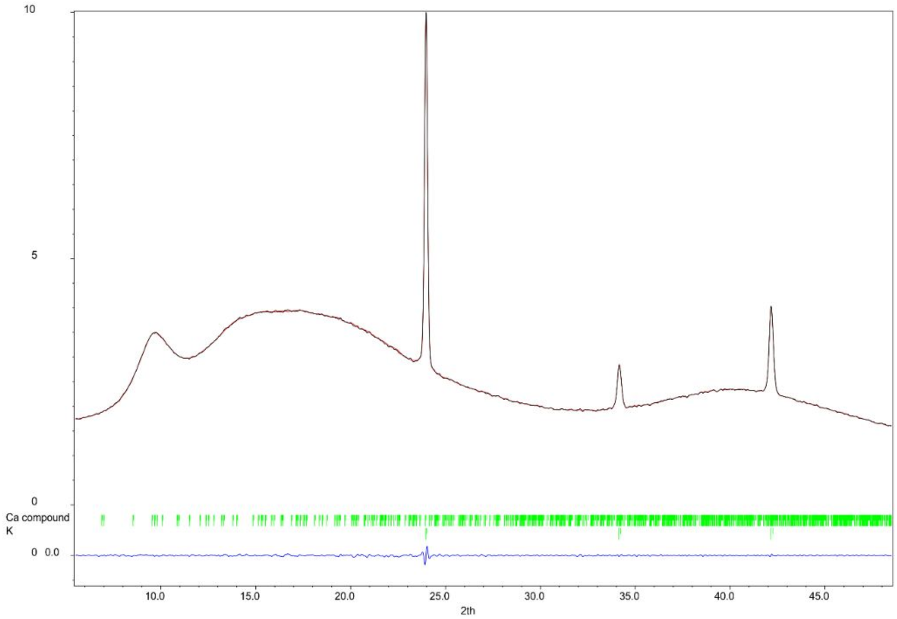

Figure S14: PXRD analysis of **2′** obtained using an excess of potassium (**1**:K 1:1.1). Black = measured observed data, Red = fitted profile, Green = expected peak positions tick marks, Blue = Observed - fitted difference

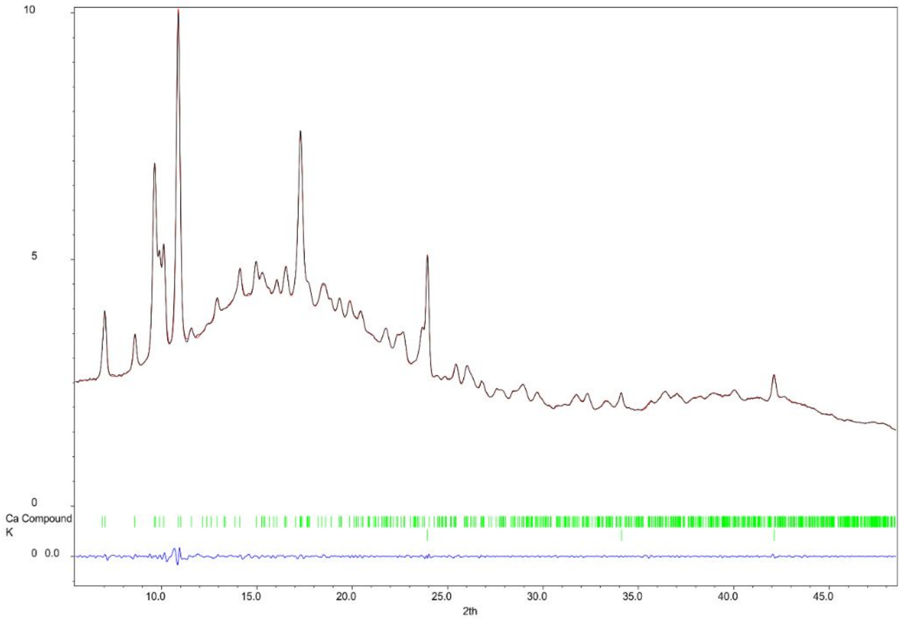

Figure S15: PXRD analysis of **2** (incomplete conversion). Material was obtained using an IKA ULTRA-TURRAX mixer mill within a 25 mL PTFE jar and four 3 mm ball bearings (4000 rpm, 30 minutes). Red = fitted profile, Green = expected peak positions tick marks, Blue = Observed - fitted difference

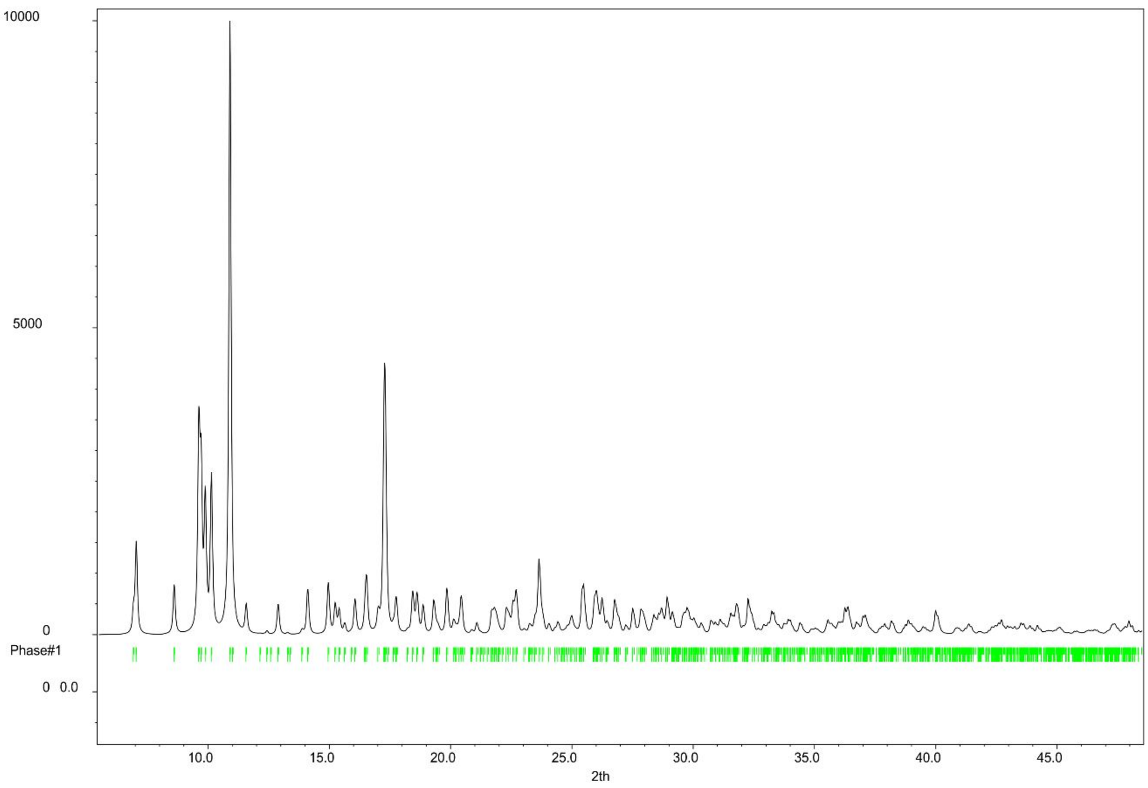

Figure S16: Simulation (black) of PXRD pattern of **1,** Green = expected peak positions tick marks.

S4. UV-vis measurements

The solid-state UV–vis absorption spectrum of **2** was run on a powdered sample using an Edinburgh Instruments FP920 Phosphorescence Lifetime Spectrometer equipped with a 450 W steady state Xenon lamp (with single 300 mm focal length excitation and emission monochromators in Czerny Turner configuration), a red sensitive photomultiplier in Peltier housing (Hamamatsu R928P) and fitted with an integrating sphere. The instrument was set to measure the total spectral reflectance of the sample in a protected environment against a blank reference plug. These traces were convoluted within the software to produce an absorption spectrum. For completeness, we also measured the UV-vis spectrum of a solution of '[K(18-crown-6)]e⁻' in THF (0.1 mM) at room temperature.

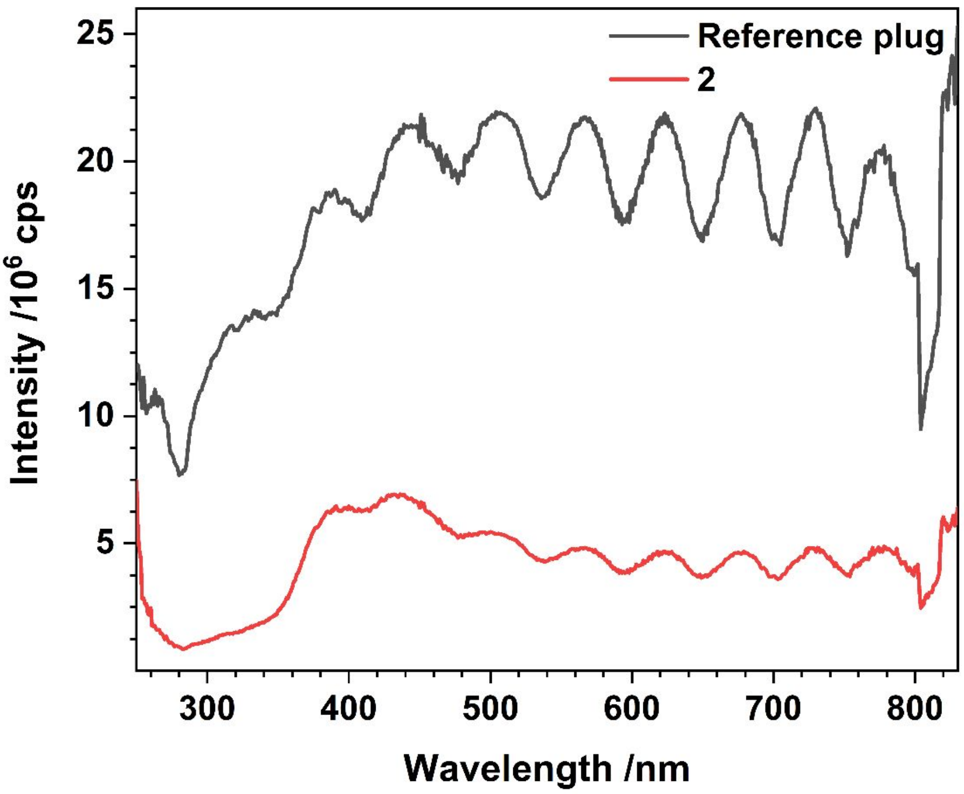

Figure S17: Raw reflected light spectrum of **2** (solid sample, r.t.).

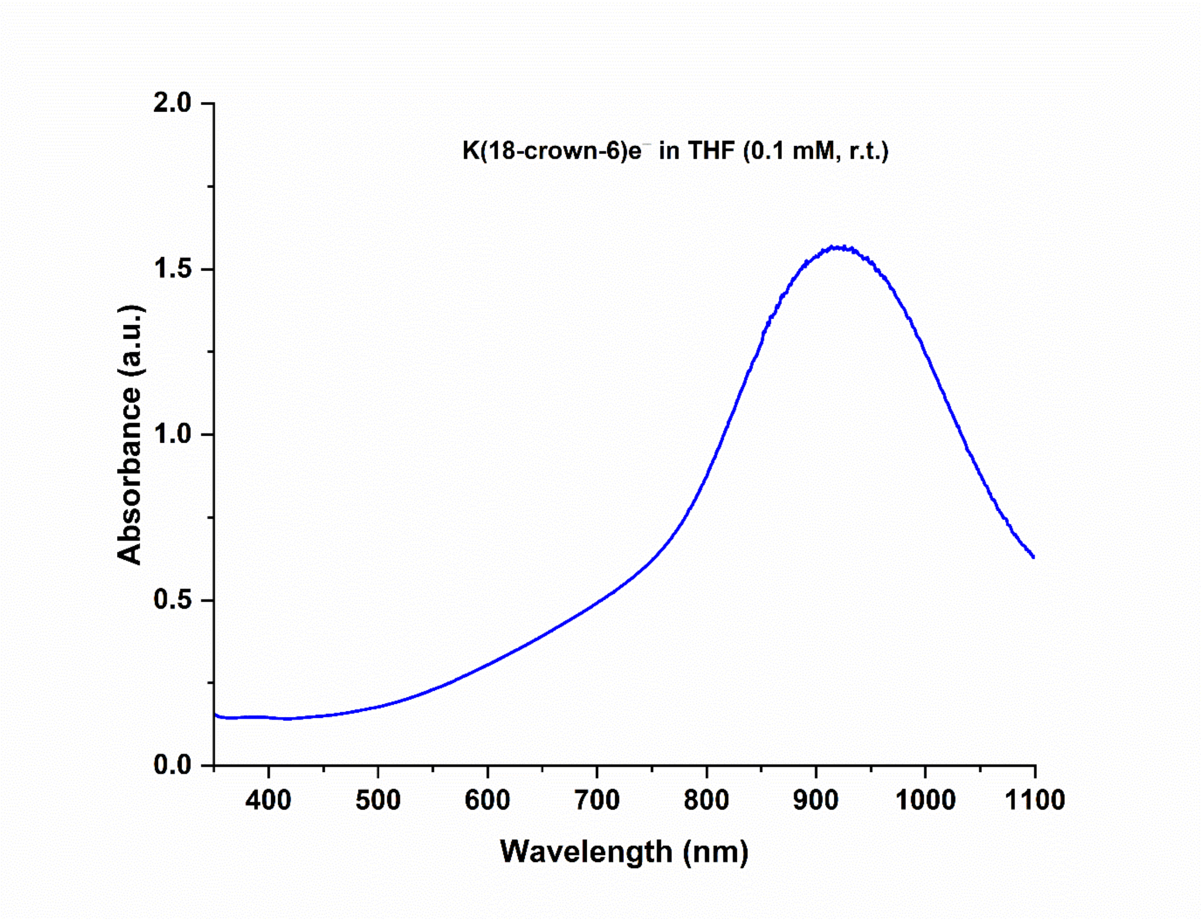

Figure S18: UV-vis spectrum of '[K(18-crown-6)]e^{-}'in THF (0.1 mM, r.t.).

S5. Electroconductivity measurements

A similar protocol to our previous report[8] is employed for the electroconductivity measurements herein.

To record the electrical conductivities, interdigitated 'finger-pattern' electrodes were scribed into ITO-coated glass (see Figure S19) using a Lasermark Primary (LaserLines) etcher and washed with isopropanol and acetone. The electrodes were subsequently examined with an optical microscope (Celestron); the inter-electrode distance was 200 μm and the length of the channel 99.8 cm on a ca. 2 cm × 1.5 cm glass slide. After transfer into a N_2-filled glovebox, the sample powders were carefully spread onto the electrode and held in place with an additional (non-conductive) glass slide. The interdigitated electrodes were connected by crocodile clamps and wired out of the glovebox and into a Keithley 2450 source meter. Then, current-voltage sweeps were applied to the samples across different potential ranges, depending on the conductivity of the sample, and linear fits were applied to extract the resistance. After removal from the N_2 glovebox, the samples were examined with optical microscopy. Owing to poor surface coverage of the powder, only around 5% of the channel between the interdigitated electrodes was found to be actively bridged by sample material, giving an effective channel length of 5 cm. The height of the powder film with a Dektak 3ST (sloan) profilometer was on average around 2 μm. Ultimately, the materials' conductivity σ was obtained as:

$$\sigma = \frac{contact\ distance}{resistance \bullet channel\ length \bullet film\ thickness}$$

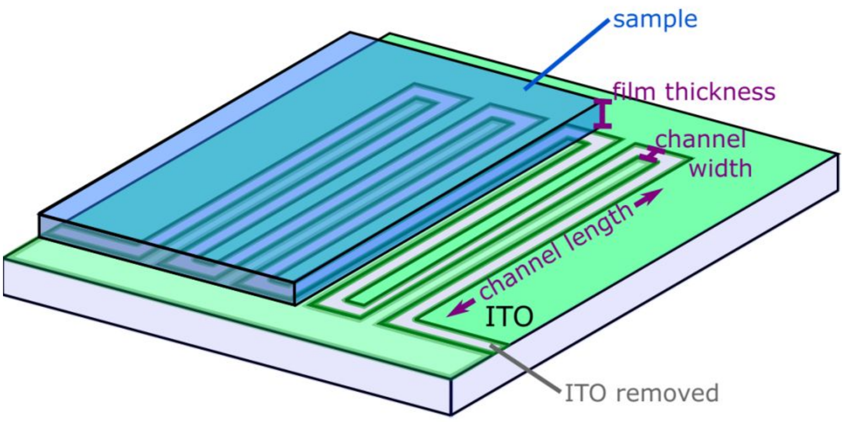

Figure S19. Instrument set-up for the electroconductivity measurements

S6. Computational details

S6.1 AIRSS Calculations

In order to determine low-energy candidate structures, ab-initio random structure searching (AIRSS) was used.[9] The geometry of several hundred random structures were optimized without spin, which was found to be sufficient in previous models of electrides.[8] A selection of low-energy structures were then optimized again with spin included. Once it became apparent that hydride geometries were overwhelmingly more favorable, hundreds more structures were produced containing H atoms, which were then pre-optimized using the MACE-MP-0 universal forcefield[10] with dispersion corrections, with the best structures being taken forward to be optimized at the same level of theory as the rest of the candidate structures.

Final AIRSS optmizations were carried out in VASP using a plane wave cutoff energy of 500 eV. Projector-augmented wave (PAW) pseudopotentials were employed where the following orbitals were treated as valence: 1*s* for H; 2*s*2*p* for C and N; 3*s*3*p* for Si; and 3*s*3*p*4*s* for K and Ca.[11] All geometry optimizations were carried out using the r2SCAN+vv10 functional[12] (a dispersion-corrected meta generalized gradient approximation) until the forces on atoms were less than 0.05 eV $Å^{-1}$.

As the stable structures included hydride-like geometries with anionic electrons highly localized in space, it was possible to use Gaussian basis sets without the risk of compromising the completeness of the basis so long as ghost atoms were included as floating basis centres. Considering this, detailed analysis of the most stable predicted structure and possible defective dimer complexes was carried out in CP2K,[13] which allows for faster calculations than in VASP. All calculations used the r2SCAN0+vv10 dispersion-corrected hybrid functional[14] with 25% Fock exchange included. Triple-ζ MOLOPT basis sets with GTH pseudopotentials were employed, the anionic electrons used the same basis as H. Hybrid DFT-calculations were accelerated using the auxiliary density matrix method using FIT11 sets for K and Ca and pFIT3 sets for all other elements. For the calculations of the defective complexes, a wavelet Poisson solver was used without periodic boundary conditions to avoid errors due to the interaction of image charges.

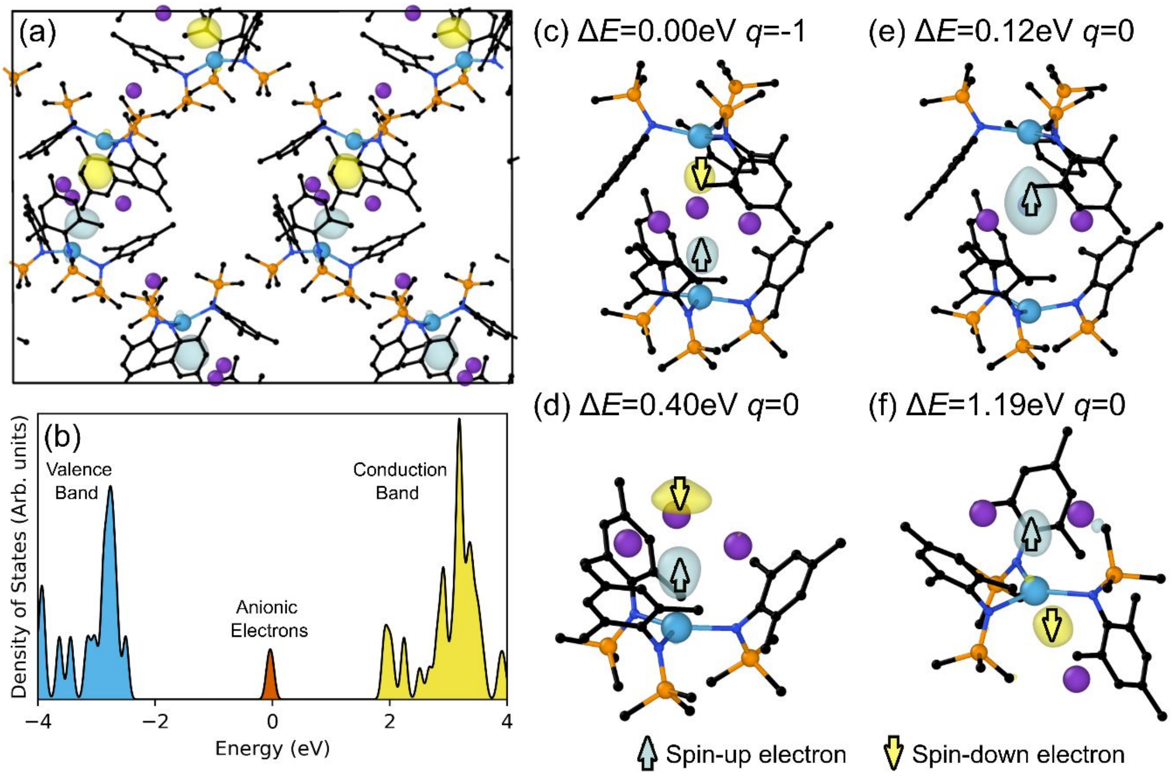

Figure S20A: Adapted version of Figure 3 from the manuscript with additional labelling of electronic spin states.

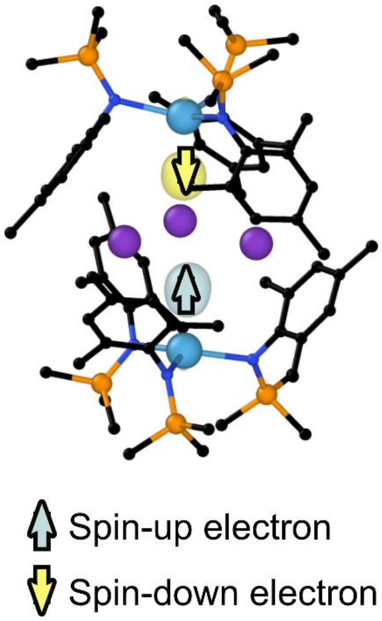

Figure S20B: Structure of dimeric **2** (Figure 3 (c) and Figure S28 (c)) with additional labelling of electronic spin states.

S6.2 DFT Methodology

DFT calculations were run with Gaussian 16 (A.03).[15] The Ca, K and Si centres were described with the Stuttgart RECPs and associated basis sets,[16] and the 6-31G** basis set was used for all other atoms (BS1).[17,18] A polarization function was also added to Si (ζ_d = 0.284) and K (ζ_d = 1.000). Initial BP86 optimizations were performed using the 'grid = ultrafine' option,[19,20] with all stationary points being fully characterized via analytical frequency calculations as minima (all positive eigenvalues). All energies were recomputed with a larger basis set featuring 6-311++G** basis sets on all atoms (BS2). Corrections for the effect of benzene (ε = 2.2706) solvent were run using the polarizable continuum model and BS1.[21] Single-point dispersion corrections to the BP86 results employed Grimme's D3 parameter set with Becke-Johnson damping as implemented in Gaussian.[22]

The Quantum Theory of Atoms in Molecules (QTAIM, AIMALL program[23]) and Natural Bonding Orbital (NBO7[24,25]) analyses were performed on the BP86-optmised geometry of the anion of **3**. The QTAIM topological analyses used wavefunction files obtained with Gaussian 16 (C.01) at the BP86/6-311++G** level, whilst NBO analyses were carried out with NBO 7 within Gaussian (C.01) at the same methodology level as the QTAIM calculations. Contour plots were generated in the AIMStudio package, using critical point (CP) visualisation threshold values of 0.02 e·Å^{-3} (solid line BCP = strong) and 0.005 e·Å^{-3} (dashed line BCP = weak). The NBO energies of donor-acceptor interactions ("$\Delta E^{(2)}$") between the various molecular fragments of the structures were estimated with second-order perturbation theory analysis of the Fock matrix in the NBO basis, as calculated by NBO7, with selected donor-acceptor NBO interactions provided. Wiberg bond indices (WBI) were calculated using NBO v7.0.

*S6.3 DFT Electronic Structure Analysis of **3***

Natural Bond Orbital Analysis

Wiberg Bond Indices (WBIs), with atom numbering in Figure S20

Ca1 – Ca3	0.0001	H2 – H4	0.0027		
Ca1 – H2	0.1369	Ca3 – H4	0.1389		
Ca1 – H4	0.0010	Ca3 – H2	0.0027		
Ca1 – K5	0.0046	Ca1 – K6	0.0045	Ca1 – K7	0.0046
Ca3 – K5	0.0046	Ca3 – K6	0.0047	Ca3 – K7	0.0047
H2 – K5	0.0383	H2 – K6	0.0367	H2 – K7	0.0374
H4 – K5	0.0360	H4 – K6	0.0378	H4 – K7	0.0359
Ca1 – N14	0.0773	Ca1 – N15	0.0739	Ca1 – N16	0.0730
Ca3 – N17	0.0755	Ca3 – N18	0.0747	Ca3 – N19	0.0757

Natural Bonding Orbitals

There were no NBOs calculated to involve bonding between the Ca, H and K atoms, suggesting an electrostatic / ionic interaction within the anion of **3**.

(NB. There were core and lone pair orbitals for these atoms, but nothing showing any bonding between Ca, H or K and any other atom.)

Second Order Perturbation Energies

Analysis of the NBO calculated Perturbation Energies ($\Delta E^{(2)}$) showed no donor-acceptor interactions for Ca or K atoms. However, the lone pair of the hydrogen atoms (H2 and H4) were shown to interact with the antibonding lone pair orbitals for their nearest Ca and K atoms. These values are given below, and are in units of kcal/mol.

Donor NBO	Acceptor NBO	$\Delta E^{(2)}$	Donor NBO	Acceptor NBO	$\Delta E^{(2)}$

154. LP(1) H2	390. LV(1) Ca1	13.47	155. LP(1) H4	391. LV(1) Ca3	14.09
154. LP(1) H2	392. LV(1) K5	3.03	155. LP(1) H4	392. LV(1) K5	2.83
154. LP(1) H2	393. LV(1) K6	2.95	155. LP(1) H4	393. LV(1) K6	2.96
154. LP(1) H2	394. LV(1) K7	2.99	155. LP(1) H4	394. LV(1) K7	2.77

QTAIM Analysis

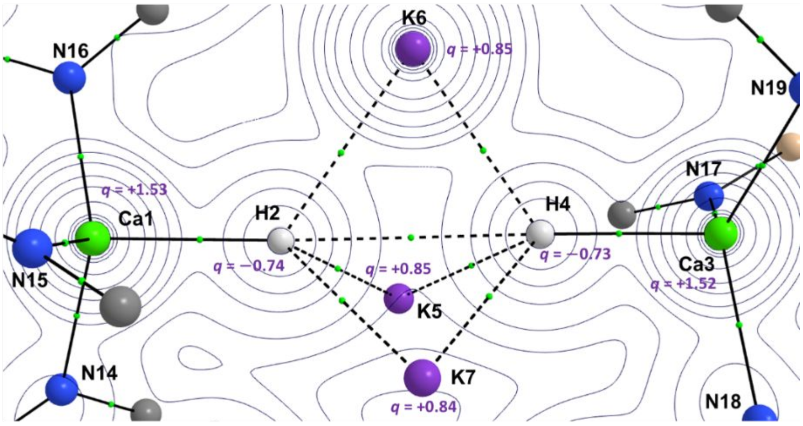

Figure S21: Bader charges (q, purple) for the core atoms of the anion component of **3** from QTAIM calculations on the BP86-optimised geometry.

Electron density (ρ) Contour Plot

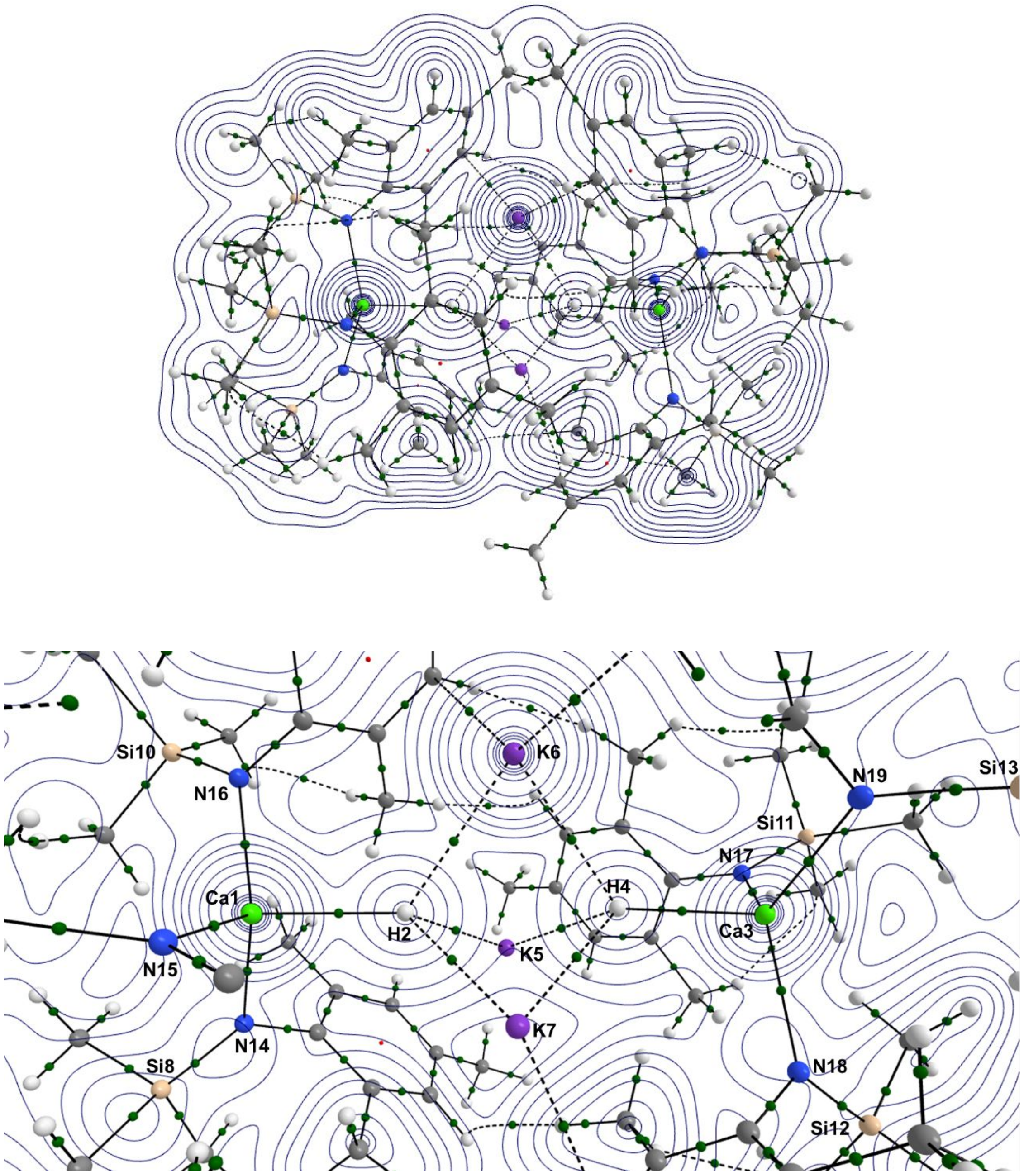

Figure S22A: Contour plot of ρ(r) of the DFT-optimized anion of **3**.

Table S2. Selected QTAIM BCP data for anion of **3**

**BCP**	**ρ(r)**	**∇^2ρ(r)**	**ε**	**G(r)**	**V(r)**	**H(r)**
H2 - H4	0.005034	+0.007452	0.047408	+0.001613	-0.001362	-0.001625
Ca1 - H2	0.026262	+0.055751	0.009217	+0.014892	-0.015846	-0.026480
Ca3 - H4	0.026234	+0.055978	0.002908	+0.014928	-0.015862	-0.026303
H2 - K5	0.010418	+0.026437	0.039656	+0.005879	-0.005149	-0.007588
H4 - K5	0.009234	+0.023365	0.048880	+0.005109	-0.004377	-0.006505
H2 - K6	0.009121	+0.022981	0.049431	+0.005022	-0.004299	-0.006388
H4 - K6	0.010450	+0.026346	0.034138	+0.005870	-0.005153	-0.007578
H2 - K7	0.009457	+0.023881	0.040789	+0.005245	-0.004519	-0.006701
H4 - K7	0.009623	+0.024298	0.047186	+0.005348	-0.004621	-0.006857
Ca1 - N14	0.039786	+0.151569	0.167309	+0.036897	-0.035902	-0.046963
Ca1 - N15	0.040371	+0.156698	0.193809	+0.038071	-0.036968	-0.048523
Ca1 - N16	0.038637	+0.148490	0.191555	+0.035853	-0.034583	-0.045720
Ca3 - N17	0.041160	+0.159858	0.190813	+0.038966	-0.037968	-0.049593
Ca3 - N18	0.040500	+0.157103	0.191450	+0.038194	-0.037113	-0.048585
Ca3 - N19	0.041090	+0.159030	0.189163	+0.038791	-0.037825	-0.049452

Table S3. Selected QTAIM atomic data for anion of **3**

Atom	q(A)	L(A)	N(A)	Vol(A)	%Loc(A)
Ca1	+1.525345	+0.000029	18.474655	106.761068	96.638769
H2	-0.736425	-0.000017	1.736425	133.033326	77.797187
Ca3	+1.521895	-0.000205	18.478105	106.411304	96.602839
H4	-0.734756	+0.000001	1.734756	129.301951	77.501661
K5	+0.845435	+0.000014	18.154565	163.754390	98.509111
K6	+0.846105	+0.000120	18.153895	165.073482	98.519207
K7	+0.841805	+0.000010	18.158195	163.223954	98.472725
Si8	+2.770185	-0.000147	11.229815	37.622704	90.520310
Si9	+2.778051	+0.000091	11.221949	37.436193	90.553858
Si10	+2.770760	-0.000369	11.229240	37.357207	90.521091
Si11	+2.773156	-0.000400	11.226844	37.412212	90.533300
Si12	+2.773932	-0.000229	11.226068	37.362159	90.536180
Si13	+2.774417	-0.000487	11.225583	37.410256	90.538482
N14	-1.633525	-0.000472	8.633525	124.068300	83.007471
N15	-1.639089	+0.000045	8.639089	121.278043	82.874581
N16	-1.624130	-0.000062	8.624130	118.880823	82.810524
N17	-1.630751	-0.000083	8.630751	118.063778	82.821953
N18	-1.631180	+0.000135	8.631180	118.224338	82.820650
N19	-1.633376	-0.000250	8.633376	117.687379	82.810318

Laplacian ($\nabla^2\rho$) Contour Plot

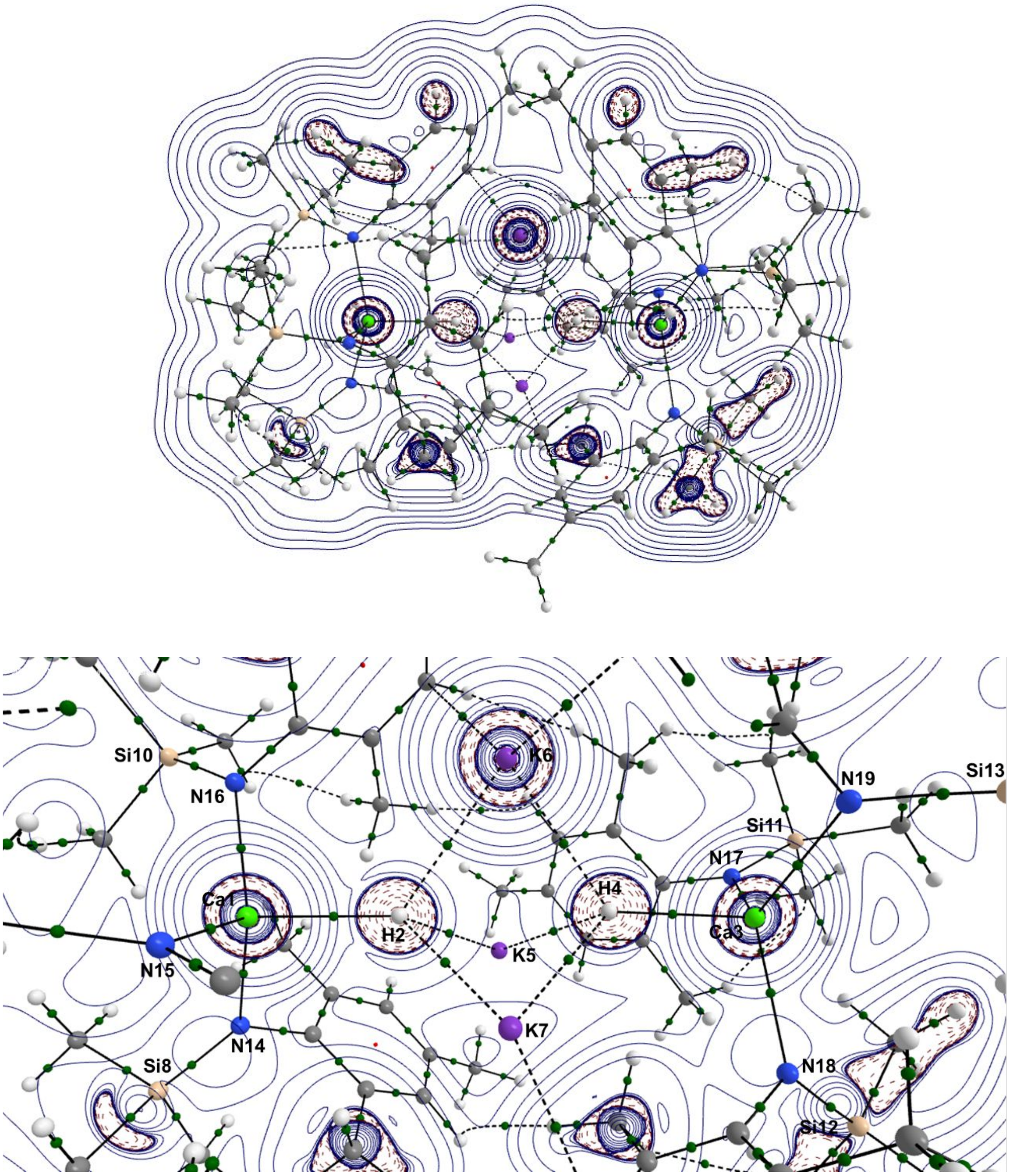

Figure S22B: Contour plot of $\nabla^2\rho(r)$ of the DFT-optimized anion of **3**.

S6.3 DFT Mechanistic Studies

Formation of 3 from 1

*Ligand Conformations in **1***

When −90 < τ < −135 °, the mesityl group could be classed as "*up*" (*u*), and when +70 < τ < +110 ° classed as "*down*" (*d*), based on the slight pyramidal nature of the trigonal planar Ca centre, with the molecule orientated such that the Ca centre is minimally above the {NNN} ligand plane. Five different conformations were isolated (see Table S4) and are close in free energy, with conformers **1a**, **1b** and **1c** having essentially the same geometry. To further probe the flexibility of the ligand and its ability to adapt to the structural environment, one ligand was rotated about the Ca–N bond in **1a** (Scheme 2). As the ligand is rotated, when the Mes and $SiMe_3$ groups need to pass through the {NNN} ligand plane, there is a large increase in energy of *ca.* 16 kcal mol^{-1}. Naturally as the ligand rotates, the other two ligand's orientations respond to the structural changes.

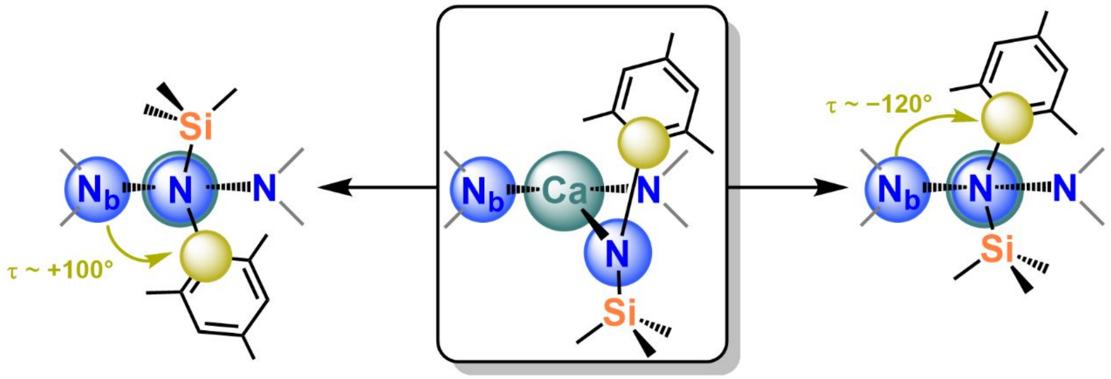

Scheme S2: The dihedral angle (τ = N_b–Ca–N–C_{ipso}) describing the orientation of the N(Mes)($SiMe_3$) mesityl groups relative to the N–Ca–N plane

Table S4 – the Free Energy values (ΔG_{bnz} in kcal mol^{-1}) of different conformers of the anion **1**, [Ca{N(Mes)($SiMe_3$)}$_3$]$^-$, alongside the dihedral (τ, in °) to describe the orientation of all three N(Mes)($SiMe_3$) ligands.

**Computed Structure**	**Structural Basis**	**Conformer**	ΔG_{bnz} [kcal mol^{-1}]	τ_1 [°]	τ_2 [°]	τ_3 [°]
**1a**	LILKAW – 2259496[a]	*uuu*	0.0	−98.3	−119.4	−137.7
**1b**	Half of **3** crystal structure	*uuu*	0.1	−98.3	−118.7	−135.1
**1c**	LILLAX – 259504 (**1**)[b]	*uuu*	0.4	−98.3	−118.3	−134.6
**1d**	LILKIE – 2259501[c]	*udd*	2.8	−59.8	+90.3	+105.3
**1e**	Manually built	*uuu*	6.3	−82.4	−93.5	−48.5

[a]CSD Entry for [Sr{N(Mes)($SiMe_3$)}$_3$K].

[b]CSD Entry for [Ca{N(Mes)($SiMe_3$)}$_3$K] (**1**).

[c]CSD Entry for [Mg{N(Mes)($SiMe_3$)}$_3$K].

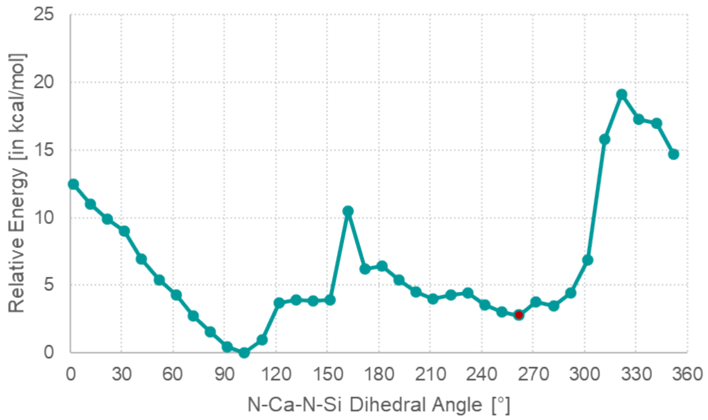

Figure S23 – Electronic Relative Energy (E in kcal mol^{-1}) rotation profile for one of the N(Mes)($SiMe_3$) ligands in **1a**. The red data point depicts the original orientation of the ligand (τ = 261.7°).

*Association of Benzene to **1***

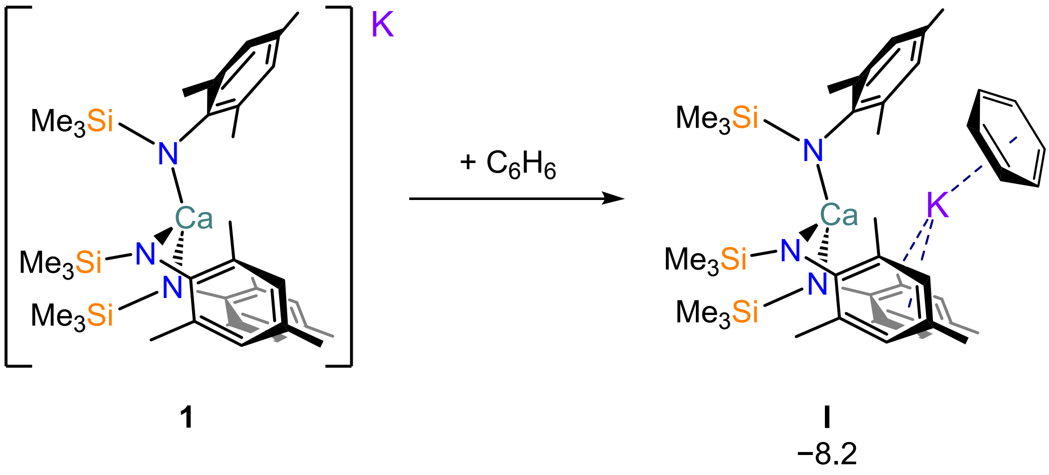

Figure S24: The Free energy addition of benzene to **1** in kcal mol^{-1} at the BP86-D3BJ(C_6H_6)/BS2//BP986/BS1 level of theory.

Coordination of benzene to **1** was unsuccessful. In all optimisations, the benzene associated to the K cation present in the computational model instead of the Ca centre, with a free energy of −8.2 kcal mol^{-1} (see Figure S24). Attempts to model benzene association to only the anionic Ca complex, saw an increase in free energy to +4.9 kcal mol^{-1}, with the benzene far from the Ca centre (**I'**; Ca···H_{bnz} = 5.96 Å). From the multiple efforts made to try and associate benzene to the Ca centre, it is logical to conclude that there is insufficient space at the Ca for the benzene (or indeed pyridine) to coordinate and is prevented by the mesityl substituents of the ligands. No Wheland intermediate was ever successfully isolated, and therefore we can confidently conclude that the Ca centre does not facilitate the C–H activation of the benzene (or pyridine).

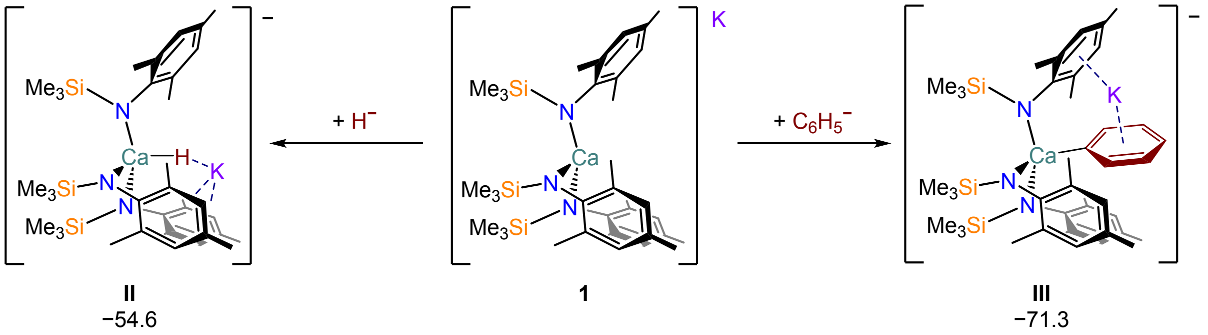

Figure S25: Addition of benzene to **1** in kcal mol^{-1} at the BP86-D3BJ(C_6H_6)/BS2//BP986/BS1 level of theory.

Coordination of a hydride or phenyl anion to **1** is energetically favoured by 54.6 and 71.3 kcal mol^{-1} forming anionic intermediates **II** and **III** respectively (see Figure S25). However, neither

of these values take into account the corresponding cation of the benzene substrate (*i.e.* either $C_6H_6^+$ or a proton) but the equivalent anion. Nor do they incorporate the free energy gain that the formation of biphenyl would provide.

Finally, the formation of **3**, [K(η^6-C_6D_6)$_4$][{Ca[N(Mes)($SiMe_3$)](H)}$_2$K$_3$], and the biphenyl C_6H_5-C_6H_5 from **1**, four potassium cations and six benzenes, gives a formation Free energy of −254.9 kcal mol^{-1}.

Formation of 4 from 1

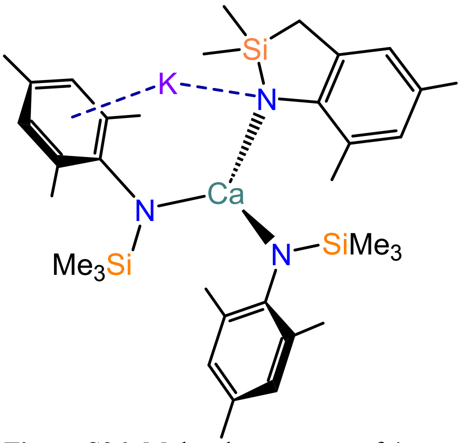

Figure S26: Molecular structure of **4**

The free energy of formation for **4** + CH_4, relative to **1**, was computed to be −22.7 kcal mol^{-1}.

To mechanistically understand this process, a range of structures were optimised, where either a H atom or CH_3 group had been removed from **1**. This provided a range of cationic and anionic species, as a proton, a hydride, a methyl anion or cation were deleted from **1**. Across all the species optimised, there was a clear preference for anion loss to occur from the $SiMe_3$ group (leaving a cationic Ca complex behind) or cation loss from the methyl group on the Mes group (leaving an anionic Ca complex behind). Pathways involving proton, hydride, methyl cation and anion loss were explored from both the $SiMe_3$ group or the methyl group of the Mes substituent, however, only one full reaction process was fully mapped. The pathway shown in Figure S26, starts from the loss of a proton at the methyl position on one of the Mes ligand substituents, **IV**. The CH_2 group then attacks the adjacent $SiMe_3$ group via **TS(IV-V)** as one of the methyl groups of the attacked $SiMe_3$ substituent then migrates to the Ca centre, with a facile barrier of 4.1 kcal mol^{-1} to exergonically form intermediate **V** at −4.1 kcal mol^{-1}. The potassium ion then travels (ΔΔG = 2.0 kcal mol^{-1}) towards the methyl anion in **TS(V-VI)** to give intermediate **VI** at −8.4 kcal mol^{-1}. Finally, transfer of the anion to the potassium affords **VII** (ΔG_{bnz} = −5.9 kcal mol^{-1}).

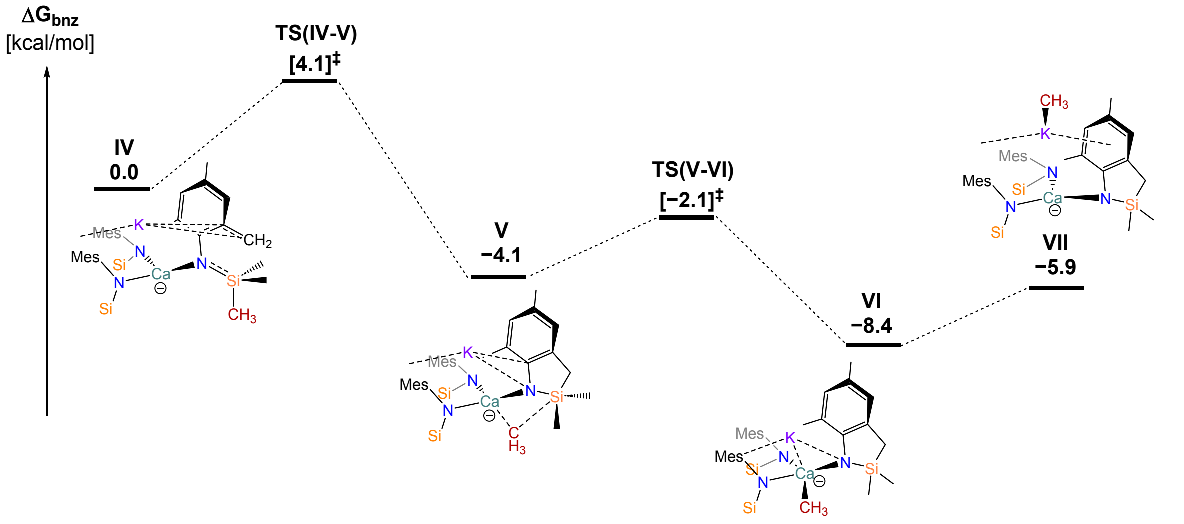

Figure S27: Addition of benzene to **1** in kcal mol^{-1} at the BP86-D3BJ(C_6H_6)/BS2//BP986/BS1 level of theory.

S6.4 DFT Breakdown of Energy Contributions

The following tables detail the evolution of the relative energies as the successive corrections to the initial SCF energy are included. Terms used are:

$\boldsymbol{\Delta E_{BSI}}$	SCF energy computed with the BP86 functional with BS1
$\boldsymbol{\Delta H_{BSI}}$	Enthalpy at 0 K with BS1
$\boldsymbol{\Delta G_{BSI}}$	Free energy at 298.15 K and 1 atm with BS1
$\boldsymbol{\Delta G_{BSI/bnz}}$	Free energy corrected for benzene solvent with BS1
$\boldsymbol{\Delta G_{BSI/bnz+D3BJ}}$	Free energy corrected for benzene and dispersion effects with BS1
$\boldsymbol{\Delta E_{BS2}}$	SCF energy computed with the BP86 functional with BS2
$\boldsymbol{\Delta G_{bnz}}$	Free energy corrected for basis set (BS2), dispersion effects and benzene solvent

In each case the final data used in the main article are highlighted in bold.

Table S5. Relative energies for conformer structures of **1** in kcal mol^{-1}. Data in bold are those used in the main text.

		ΔE_{BSI}	ΔH_{BSI}	ΔG_{BSI}	$\Delta G_{BSI/bnz}$	$\Delta G_{BSI/bnz+D3BJ}$	ΔE_{BS2}	ΔG_{bnz}
$[Ca\{N(Mes)(SiMe_3)\}_3]^-$	**1a**	0.0	0.0	0.0	0.0	0.0	0.0	**0.0**
	**1b**	2.8	2.3	1.2	0.8	0.1	2.8	**0.1**
	**1c**	2.8	2.2	1.5	1.1	0.5	2.8	**0.4**
	**1d**	3.6	3.7	3.6	3.8	2.6	3.8	**2.8**
	**1e**	5.7	6.1	6.3	6.7	6.2	5.8	**6.3**

Table S6. Relative energies for computed structures in kcal mol^{-1} to **1a** + $\mathbf{C_6H_6}$. Data in bold are those used in the main text.

	ΔE_{BSI}	ΔH_{BSI}	ΔG_{BSI}	$\Delta G_{BSI/bnz}$	$\Delta G_{BSI/bnz+D3BJ}$	ΔE_{BS2}	ΔG_{bnz}
**I**	−21.7	−19.6	−6.2	3.5	−13.0	−16.9	**−8.2**
**I′**	76.8	76.6	76.1	1.9	3.8	77.9	**4.9**
**II**	13.2	6.7	7.8	−32.7	−32.4	−9.0	**−54.6**
**III**	54.6	47.9	56.5	−7.1	−21.4	4.7	**−71.3**

Table S7. Relative energies for computed structures in kcal mol^{-1} to the anionic **IV** (which is based on **1a** with a proton removed from the Mes group). Data in bold are those used in the main text.

	ΔE_{BSI}	ΔH_{BSI}	ΔG_{BSI}	$\Delta G_{BSI/bnz}$	$\Delta G_{BSI/bnz+D3BJ}$	ΔE_{BS2}	ΔG_{bnz}
**IV**	0.0	0.0	0.0	0.0	0.0	0.0	**0.0**
**TS(IV-V)**	4.6	4.1	4.5	4.6	3.6	5.0	**4.1**
**V**	−3.1	−4.0	−5.2	−5.6	−5.6	−4.6	**−4.1**
**TS(V-VI)**	−2.5	−3.7	−4.3	−4.6	−4.1	−0.5	**−2.1**
**VI**	−7.9	−9.2	−10.2	−12.2	−10.0	−6.2	**−8.4**
**VII**	−4.0	−5.1	−6.5	−7.7	−8.3	−1.6	**−5.9**

Cartesian Coordinates and DFT Computed Energies [in Hartrees]

3

```
SCF (BP86) Energy = -4285.74893374
Enthalpy 0K = -4283.652271
Enthalpy 298K = -4283.490792
Free Energy 298K = -4283.885440
Lowest Frequency = 4.9834 cm-1
Second Frequency = 7.0562 cm-1
SCF (BP86-D3BJ) Energy =
                   -4286.52154667
SCF (C6H6) Energy = -4285.77618007
SCF (BS2) Energy = -9568.73732437
```

```
Ca  4.47680 -2.68582 -0.05402
H   3.06815 -0.85919 -0.01825
Ca -0.35890  3.46052  0.00348
H   1.05517  1.66368 -0.02098
K   1.40053 -0.28994  2.10182
K   3.97694  1.88374 -0.15274
K   1.27680 -0.28160 -2.05855
Si  3.13966 -6.14801  0.82038
Si  5.91887 -3.83516 -3.08835
Si  7.57896 -2.65328  2.10345
Si -0.74242  5.56465  3.04296
Si -4.00503  2.97391 -0.51469
Si -0.07584  6.27891 -2.39163
N   3.13907 -4.39195  1.01503
N   4.62873 -2.83776 -2.44008
N   6.33438 -1.79359  1.19268
N  -0.39306  3.99489  2.32170
N  -2.39331  2.43003 -0.87125
N   0.68086  4.99381 -1.46220
C   2.15277 -3.86676  1.85542
C   2.40331 -3.57589  3.24383
C   1.36365 -3.12594  4.08339
H   1.58360 -2.96704  5.14796
C   0.04241 -2.94207  3.62760
C  -0.19375 -3.17080  2.25595
H  -1.21681 -3.06185  1.86519
C   0.81726 -3.60455  1.37712
C   3.76815 -3.85256  3.82821
H   3.83049 -3.51177  4.87568
H   4.56932 -3.36321  3.24855
H   3.99479 -4.93363  3.80535
C  -1.09030 -2.61846  4.58031
H  -1.67931 -3.52243  4.82819
H  -1.79525 -1.88036  4.15690
H  -0.71372 -2.20862  5.53219
C   0.49185 -3.86473 -0.07308
H   1.20962 -3.35463 -0.74057
H  -0.53260 -3.53289 -0.31766
H   0.57041 -4.93628 -0.32739
C   4.69958 -7.02966  1.49816
H   5.62294 -6.70103  0.99352
H   4.60219 -8.11927  1.33728
H   4.83634 -6.86118  2.57977
C   3.04999 -6.69097 -1.01338
H   3.24400 -7.77483 -1.10597
H   3.80492 -6.16203 -1.61953
H   2.06328 -6.48432 -1.46132
C   1.66639 -6.95803  1.74397
H   1.68671 -8.05229  1.59504
H   0.68801 -6.58732  1.39460
H   1.72250 -6.76594  2.82935
C   3.81093 -2.13317 -3.32773
C   4.11408 -0.77645 -3.71314
C   3.33331 -0.10491 -4.67429
H   3.64543  0.89924 -4.99324
C   2.19820 -0.68935 -5.27341
C   1.84495 -1.97941 -4.83139
H   0.96093 -2.46639 -5.26583
C   2.60657 -2.69838 -3.88630
C   5.30680 -0.07810 -3.10735
H   5.35479 -0.25054 -2.01722
H   5.27857  1.00680 -3.30874
H   6.25894 -0.46818 -3.50363
C   1.43593  0.01123 -6.37958
H   1.88980 -0.18611 -7.36932
H   1.42678  1.10663 -6.24341
H   0.38810 -0.33156 -6.43169
C   2.16913 -4.09019 -3.49471
H   1.08149 -4.22308 -3.63505
H   2.43614 -4.30640 -2.44887
H   2.67332 -4.86182 -4.10280
C   7.24853 -2.88841 -4.09859
H   7.80192 -2.16577 -3.47521
H   6.79607 -2.33824 -4.94241
H   7.98328 -3.59616 -4.52371
C   5.37049 -5.25500 -4.25688
H   4.83612 -4.86305 -5.14010
H   4.71148 -5.98064 -3.75153
H   6.25661 -5.80347 -4.62516
C   6.79724 -4.61706 -1.58718
H   6.13531 -5.29752 -1.02184
H   7.17696 -3.84014 -0.89925
H   7.66634 -5.21340 -1.91588
C   6.42403 -0.39377  1.24599
C   5.72064  0.35811  2.25691
C   5.91239  1.74761  2.39129
H   5.43019  2.26550  3.23237
C   6.74406  2.48396  1.52177
C   7.34265  1.77081  0.46435
H   7.98376  2.31290 -0.24470
C   7.20437  0.37549  0.30818
C   4.79946 -0.35689  3.21673
H   4.11775  0.35375  3.71608
H   5.35386 -0.89353  4.00490
H   4.21418 -1.13251  2.69147
C   7.03406  3.95208  1.75971
H   7.33343  4.46471  0.82978
H   7.86280  4.08430  2.48102
H   6.16062  4.48521  2.17402
C   7.94206 -0.30820 -0.82134
H   7.41313 -1.21988 -1.14160
H   8.95253 -0.62486 -0.50911
H   8.06195  0.36769 -1.68528
C   7.69286 -2.13182  3.95067
H   7.76663 -1.03608  4.06286
H   8.59669 -2.57243  4.40923
H   6.82289 -2.48068  4.53343
C   9.37367 -2.42794  1.45778
H  10.06471 -3.03572  2.07042
H   9.70821 -1.37851  1.52949
H   9.48124 -2.75343  0.40934
C   7.22432 -4.52230  2.07329
```

H	6.15977	-4.73903	2.25315
H	7.80698	-5.02053	2.86845
H	7.51064	-4.98249	1.11382
C	0.05726	2.99463	3.19666
C	1.45683	2.83680	3.50384
C	1.87737	1.92297	4.49006
H	2.94256	1.89572	4.75832
C	0.97882	1.08220	5.17847
C	-0.37280	1.14474	4.78605
H	-1.09968	0.48580	5.28068
C	-0.84150	2.05830	3.81974
C	2.48798	3.67293	2.78383
H	2.24807	3.75100	1.70823
H	3.49679	3.24192	2.90301
H	2.52360	4.70996	3.15531
C	1.44119	0.20669	6.32501
H	1.51256	0.78457	7.26598
H	2.43943	-0.22614	6.13876
H	0.74146	-0.62562	6.51025
C	-2.31429	2.08259	3.47582
H	-2.82167	1.18919	3.88449
H	-2.46966	2.11730	2.38354
H	-2.81753	2.97432	3.88705
C	-2.22866	5.56158	4.26326
H	-2.37298	6.57170	4.68839
H	-2.05613	4.87170	5.10812
H	-3.17056	5.27123	3.76679
C	-1.15414	6.80424	1.65498
H	-0.45502	6.70336	0.80718
H	-1.07261	7.83678	2.03780
H	-2.18123	6.67038	1.27811
C	0.68623	6.32631	4.07597
H	1.52883	6.65083	3.44190
H	1.07287	5.61752	4.82865
H	0.31835	7.21601	4.61868
C	-2.22657	1.29697	-1.67974
C	-2.05278	-0.00922	-1.09641
C	-1.93440	-1.15558	-1.91046
H	-1.82683	-2.13624	-1.42496
C	-1.95907	-1.08855	-3.31912
C	-2.04577	0.19757	-3.88948
H	-2.02748	0.29390	-4.98357
C	-2.16207	1.37120	-3.11786
C	-1.98245	-0.15341	0.40552
H	-2.95377	0.03373	0.89609
H	-1.28916	0.59440	0.83182
H	-1.64074	-1.16342	0.69238
C	-1.94012	-2.33811	-4.17555
H	-2.95646	-2.75868	-4.30364
H	-1.31578	-3.13274	-3.73191
H	-1.55084	-2.12992	-5.18591
C	-2.20236	2.71118	-3.81463
H	-1.51445	3.42497	-3.33118
H	-3.20257	3.17381	-3.77558
H	-1.91935	2.61077	-4.87580
C	-5.10374	3.54262	-1.98731
H	-4.67003	4.41937	-2.49624
H	-6.10589	3.83569	-1.62101
H	-5.24101	2.74584	-2.73994
C	-5.09901	1.55555	0.25044
H	-6.15813	1.87900	0.25565
H	-4.78501	1.30909	1.27900
H	-4.97157	0.66933	-0.39989
C	-3.95829	4.40421	0.73156
H	-4.92869	4.50705	1.24896
H	-3.75325	5.35760	0.21761
H	-3.17645	4.24660	1.49403
C	2.05431	4.76306	-1.64115
C	2.53063	3.82777	-2.62759
C	3.91096	3.66987	-2.86464
H	4.23062	3.00403	-3.67821
C	4.88899	4.37370	-2.13235
C	4.42641	5.20722	-1.09389
H	5.15982	5.75489	-0.48621
C	3.05597	5.41022	-0.83283
C	1.54427	3.02853	-3.44698
H	0.70598	2.67551	-2.81933
H	2.03640	2.16565	-3.92846
H	1.08007	3.63294	-4.24474
C	6.35863	4.29153	-2.49220
H	6.63543	3.29543	-2.87914
H	7.00450	4.50821	-1.62447
H	6.61864	5.02521	-3.27874
C	2.64901	6.35317	0.27653
H	3.49514	6.55123	0.95644
H	1.80863	5.94467	0.86199
H	2.30454	7.32323	-0.12089
C	0.15068	6.12953	-4.29437
H	-0.22844	7.03985	-4.79344
H	-0.39596	5.26763	-4.71375
H	1.21578	6.02496	-4.56560
C	0.53593	8.05510	-1.99810
H	0.35683	8.32898	-0.94472
H	0.00112	8.78708	-2.63087
H	1.61433	8.16868	-2.20545
C	-1.95212	6.24762	-2.03535
H	-2.18047	6.69730	-1.05504
H	-2.34202	5.21528	-2.04805
H	-2.49483	6.82564	-2.80410
K	-7.13498	-0.81949	0.43411
C	-6.67439	-1.47580	3.78280
H	-7.06257	-0.72008	4.47354
C	-5.36579	-1.35842	3.27800
H	-4.73796	-0.51001	3.56917
C	-4.85227	-2.34783	2.41778
H	-3.82762	-2.26370	2.04247
C	-5.64792	-3.45305	2.06196
H	-5.23699	-4.23596	1.41641
C	-6.95886	-3.56772	2.56237
H	-7.56975	-4.43919	2.30424
C	-7.47142	-2.57950	3.42382
H	-8.48089	-2.68346	3.83514
C	-9.21804	1.64329	1.86584
H	-8.76979	2.29935	2.61908
C	-9.82056	0.43185	2.25313
H	-9.84626	0.14803	3.31025
C	-10.41472	-0.40050	1.28535
H	-10.90434	-1.33447	1.58081
C	-10.41033	-0.01904	-0.06964
H	-10.89406	-0.66245	-0.81210
C	-9.81052	1.19448	-0.45614
H	-9.82394	1.50367	-1.50634
C	-9.21315	2.02476	0.51079
H	-8.76096	2.97586	0.21224
C	-5.55556	-1.13715	-2.64413
H	-4.64865	-0.54722	-2.81779
C	-6.81791	-0.64380	-3.02542
H	-6.89639	0.33985	-3.49995

```
C  -7.97285 -1.42128 -2.81754
H  -8.95119 -1.04594 -3.13556
C  -7.87102 -2.69624 -2.22739
H  -8.77039 -3.30543 -2.08381
C  -6.60783 -3.19265 -1.84997
H  -6.52184 -4.19584 -1.41850
C  -5.45344 -2.41308 -2.05714
H  -4.46510 -2.79537 -1.78248
C  -12.94248 -2.84912 -0.99188
H  -13.60810 -1.97988 -1.02182
C  -12.32594 -3.30416 -2.17040
H  -12.51027 -2.78923 -3.11929
C  -11.49278 -4.43656 -2.13570
H  -11.03099 -4.80471 -3.05816
C  -11.27357 -5.11137 -0.92176
H  -10.63886 -6.00366 -0.89855
C  -11.88899 -4.65551  0.25707
H  -11.73352 -5.19277  1.19879
C  -12.72423 -3.52550  0.22190
H  -13.22332 -3.18401  1.13529
```

Anion of **3**

SCF (BP86) Energy = -3328.49256715
Enthalpy 0K = -3326.790324
Enthalpy 298K = -3326.658467
Free Energy 298K = -3326.968386
Lowest Frequency = 13.7956 cm^{-1}
Second Frequency = 14.5420 cm^{-1}
SCF (BP86-D3BJ) Energy = -3329.13925282
SCF (C6H6) Energy = -3328.52230157
SCF (BS2) Energy = -8039.61832111

```
Ca -3.91351 -0.06947  0.01425
H  -1.61630 -0.01213 -0.01305
Ca  3.90632  0.06044 -0.10917
H   1.61183  0.02585  0.01916
K  -0.07102  1.86214 -1.40175
K   0.03944  0.31730  2.31578
K   0.03841 -2.21971 -0.74246
Si -5.92034  0.16609 -3.22201
Si -5.68231 -2.98994  1.06876
Si -5.85277  2.46771  2.07045
Si  5.78271  3.24516  0.29204
Si  5.56767 -0.98072 -3.24858
Si  6.00501 -1.93772  2.21835
N  -4.53679  0.59298 -2.21629
N  -4.12438 -2.40347  0.52533
N  -4.40619  1.50548  1.78342
N   4.30470  2.41588 -0.17245
N   4.21824 -1.17360 -2.14071
N   4.52800 -1.03809  1.92196
C  -3.53685  1.35771 -2.83584
C  -3.49425  2.79187 -2.72734
C  -2.52004  3.53680 -3.42260
H  -2.55125  4.63291 -3.35111
C  -1.55566  2.93598 -4.25592
C  -1.56154  1.52705 -4.32413
H  -0.83881  1.02299 -4.98130
C  -2.50384  0.74292 -3.63029
C  -4.55898  3.51237 -1.93441
H  -4.35435  4.59572 -1.88550
H  -4.63738  3.12082 -0.90598
H  -5.55595  3.37152 -2.38967
C  -0.62167  3.76021 -5.11917
H  -1.00341  3.84554 -6.15484
H   0.38602  3.31362 -5.18623
H  -0.50975  4.78707 -4.73188
C  -2.47016 -0.76006 -3.76421
H  -2.47912 -1.24787 -2.77251
H  -1.57770 -1.09118 -4.32189
H  -3.36106 -1.14780 -4.28875
C  -7.59075  0.92925 -2.66445
H  -7.88475  0.60446 -1.65266
H  -8.38926  0.61330 -3.36149
H  -7.56383  2.03229 -2.66228
C  -6.26151 -1.71881 -3.25691
H  -7.23756 -1.92976 -3.73060
H  -6.28642 -2.13481 -2.23531
H  -5.48660 -2.26822 -3.81762
C  -5.69006  0.75241 -5.03394
H  -6.57121  0.46940 -5.63723
H  -4.79770  0.31407 -5.51115
H  -5.58406  1.84974 -5.08626
C  -3.04892 -3.29779  0.47501
C  -2.14859 -3.45342  1.58970
C  -1.12457 -4.42038  1.56237
H  -0.50796 -4.55507  2.46199
C  -0.88927 -5.24584  0.44424
C  -1.70681 -5.03083 -0.68279
H  -1.54296 -5.63922 -1.58275
C  -2.75681 -4.08935 -0.69473
C  -2.32233 -2.59531  2.81928
H  -2.51099 -1.54215  2.54456
H  -1.43344 -2.65174  3.47207
H  -3.19689 -2.89992  3.41827
C   0.15623 -6.34192  0.47296
H  -0.26776 -7.29612  0.84060
H   0.99872 -6.08550  1.13820
H   0.56849 -6.53725 -0.53189
C  -3.59922 -3.94364 -1.94026
H  -3.04702 -4.27586 -2.83698
H  -3.92636 -2.90121 -2.07551
H  -4.52298 -4.54581 -1.88111
C  -5.71393 -3.67518  2.86420
H  -5.48740 -2.89071  3.60610
H  -4.98083 -4.49090  2.99360
H  -6.71176 -4.08672  3.10315
C  -6.45926 -4.40489  0.02694
H  -5.80354 -5.29311  0.00605
H  -6.64994 -4.09584 -1.01446
H  -7.42341 -4.71632  0.46941
C  -6.87687 -1.50479  1.01020
H  -7.03543 -1.13989 -0.02029
H  -6.49630 -0.66865  1.62371
H  -7.86655 -1.78515  1.41187
C  -3.34187  1.74576  2.66715
C  -2.32634  2.72163  2.35591
C  -1.33337  3.05614  3.29777
H  -0.63580  3.87045  3.05657
C  -1.23724  2.42335  4.55464
C  -2.16363  1.39553  4.81772
H  -2.11395  0.87083  5.78223
C  -3.19366  1.04695  3.91950
C  -2.35229  3.43448  1.02468
H  -1.39440  3.94782  0.83214
H  -3.15492  4.18870  0.97080
H  -2.55877  2.72595  0.20260
```

C	-0.18578	2.83070	5.56821
H	-0.52665	2.62959	6.59843
H	0.04403	3.90793	5.49530
H	0.76845	2.28713	5.43029
C	-4.17939	-0.02796	4.32026
H	-4.58652	-0.53471	3.43091
H	-5.04233	0.39520	4.86396
H	-3.70678	-0.77419	4.98184
C	-5.53105	4.36428	2.13013
H	-4.69775	4.61447	2.80972
H	-6.43070	4.88894	2.50069
H	-5.29545	4.77375	1.13271
C	-6.76816	2.12289	3.72725
H	-7.68223	2.74322	3.77925
H	-6.14640	2.37925	4.60266
H	-7.07338	1.06682	3.81916
C	-7.12359	2.15923	0.68819
H	-6.64615	2.14143	-0.30381
H	-7.87517	2.96884	0.68683
H	-7.65926	1.20615	0.82681
C	3.21501	3.21171	-0.55166
C	2.25815	3.68418	0.41727
C	1.23812	4.58077	0.04319
H	0.57992	4.98184	0.82661
C	1.06225	5.01732	-1.28570
C	1.92213	4.46113	-2.25453
H	1.79975	4.75583	-3.30588
C	2.96896	3.57547	-1.92429
C	2.35848	3.23518	1.85569
H	2.57110	2.15259	1.91555
H	1.42669	3.45631	2.40407
H	3.18611	3.72700	2.39264
C	0.04092	6.07943	-1.63800
H	0.45028	7.09676	-1.48627
H	-0.86634	6.00169	-1.01390
H	-0.27016	6.01149	-2.69422
C	3.86114	3.04194	-3.02211
H	3.42902	3.24868	-4.01664
H	4.01595	1.95382	-2.92253
H	4.86584	3.49631	-2.98891
C	6.62956	4.24111	-1.11816
H	7.53377	4.74941	-0.73509
H	5.96003	5.02051	-1.52274
H	6.94034	3.58788	-1.95114
C	7.04668	1.95458	0.90140
H	6.57335	1.21533	1.56997
H	7.85608	2.45226	1.46447
H	7.50801	1.41565	0.05776
C	5.57757	4.54754	1.69355
H	5.35664	4.07759	2.66710
H	4.77001	5.26544	1.46661
H	6.51239	5.12655	1.80777
C	3.21307	-2.08099	-2.50575
C	2.07833	-1.67335	-3.29655
C	1.13165	-2.61802	-3.74264
H	0.31871	-2.27716	-4.39906
C	1.21490	-3.98548	-3.41161
C	2.26130	-4.36599	-2.54845
H	2.34442	-5.41953	-2.24669
C	3.23726	-3.46021	-2.08771
C	1.90577	-0.22024	-3.67303
H	2.51864	0.06362	-4.54573
H	2.24315	0.43087	-2.84739
H	0.85392	0.00253	-3.92437
C	0.26484	-5.00382	-4.00769
H	0.22622	-5.92536	-3.40225
H	0.57973	-5.29934	-5.02709
H	-0.76410	-4.61200	-4.09237
C	4.33737	-3.96026	-1.18066
H	4.48752	-3.28370	-0.32264
H	5.30701	-4.01140	-1.70427
H	4.10417	-4.96919	-0.79890
C	6.59572	-2.57567	-3.55641
H	7.09463	-2.92339	-2.63574
H	7.38061	-2.38354	-4.31107
H	5.96532	-3.39815	-3.93766
C	5.09325	-0.40509	-5.02217
H	5.98984	-0.40094	-5.66901
H	4.67270	0.61486	-5.02751
H	4.35603	-1.08447	-5.48494
C	6.75871	0.32272	-2.53154
H	7.46630	0.66259	-3.30846
H	7.35008	-0.09429	-1.69958
H	6.21181	1.20720	-2.16190
C	3.54605	-1.00924	2.92152
C	2.48825	-1.98724	2.96066
C	1.54649	-1.99255	4.00993
H	0.79704	-2.79650	4.03849
C	1.56435	-1.03748	5.04672
C	2.54263	-0.02632	4.95956
H	2.58226	0.74294	5.74380
C	3.50869	0.01497	3.93503
C	2.39885	-3.03973	1.88123
H	2.62169	-2.60459	0.89090
H	1.39984	-3.50860	1.86470
H	3.14100	-3.84420	2.02172
C	0.62621	-1.13555	6.23277
H	-0.33431	-1.60576	5.95912
H	0.40599	-0.14353	6.66277
H	1.06504	-1.74872	7.04348
C	4.52970	1.12897	3.92741
H	4.23008	1.94295	4.60984
H	4.65696	1.54337	2.91337
H	5.52532	0.77313	4.24244
C	5.73548	-3.78849	2.66934
H	6.69332	-4.25145	2.96987
H	5.33607	-4.36772	1.81965
H	5.03618	-3.89616	3.51722
C	7.09911	-1.28590	3.65958
H	7.44657	-0.25489	3.47645
H	7.99356	-1.92627	3.77262
H	6.55932	-1.30184	4.62276
C	7.08213	-1.87429	0.64543
H	7.59244	-0.90133	0.55265
H	6.48064	-2.04041	-0.26456
H	7.85968	-2.65779	0.68466

1a

SCF (BP86) Energy = -1621.11850981
Enthalpy 0K = -1620.274261
Enthalpy 298K = -1620.212101
Free Energy 298K = -1620.374525
Lowest Frequency = 13.3173 cm^{-1}
Second Frequency = 17.5485 cm^{-1}
SCF (BP86-D3BJ) Energy = -1621.38388539
SCF (C6H6) Energy = -1621.14948349
SCF (BS2) Energy = -3119.22135675

```
Si  1.39136  3.28589  1.22469
Si  1.32228 -1.41053 -2.54477
Si -1.34339 -1.51628  2.84563
N   0.11714  2.35464  0.47428
N   1.91273 -0.86700 -0.99783
N  -1.50587 -1.39094  1.11673
C   0.85656  4.38483  2.71351
H   0.08419  5.11749  2.42033
H   1.72343  4.95368  3.09853
H   0.45510  3.78135  3.54583
C   2.28945  4.52252  0.05797
H   2.82814  4.00023 -0.75006
H   3.02521  5.12495  0.62227
H   1.57157  5.22242 -0.40498
C   2.69992  2.07240  1.90480
H   2.30730  1.47952  2.74893
H   3.57027  2.63842  2.28297
H   3.08123  1.37537  1.13778
C  -0.98781  2.96829 -0.15318
C  -2.19791  3.25714  0.56871
C  -3.29931  3.83589 -0.08591
H  -4.20452  4.04637  0.50121
C  -3.28849  4.14052 -1.45871
C  -2.11715  3.83651 -2.17143
H  -2.07605  4.05389 -3.24839
C  -0.98435  3.26620 -1.55997
C  -2.30724  2.92012  2.03843
H  -1.64245  3.54144  2.66161
H  -2.01473  1.87163  2.22348
H  -3.34171  3.06143  2.39630
C  -4.50518  4.72489 -2.14695
H  -5.04389  5.43185 -1.49074
H  -5.23240  3.94252 -2.43971
H  -4.22683  5.26685 -3.06782
C   0.24090  2.99542 -2.40658
H  -0.01548  2.99163 -3.48023
H   0.71511  2.03131 -2.15342
H   1.02399  3.75938 -2.25254
C  -0.54985 -0.97935 -2.56978
H  -1.14258 -1.51266 -1.80266
H  -0.97672 -1.28783 -3.54053
H  -0.74659  0.10872 -2.49777
C   1.50039 -3.29462 -2.86446
H   2.55606 -3.60388 -2.76507
H   1.16846 -3.56081 -3.88470
H   0.90465 -3.88378 -2.14789
C   2.07245 -0.59408 -4.11549
H   1.92880  0.49927 -4.11665
H   1.58844 -1.00235 -5.02217
H   3.15460 -0.79692 -4.19807
C   3.24971 -0.99540 -0.57561
C   3.65471 -2.08426  0.27026
C   4.98241 -2.18571  0.72052
H   5.25500 -3.03913  1.35795
C   5.96745 -1.23988  0.38480
C   5.56831 -0.16367 -0.42441
H   6.30743  0.60442 -0.69390
C   4.25160 -0.01897 -0.90097
C   2.65445 -3.13983  0.68205
H   2.27175 -3.70646 -0.18454
H   1.76325 -2.69850  1.16475
H   3.10681 -3.85744  1.38846
C   7.38309 -1.35185  0.91324
H   7.48930 -0.90224  1.92018
H   8.10276 -0.83559  0.25371
H   7.70326 -2.40578  0.99933
C   3.90442  1.18984 -1.74019
H   4.65526  1.98818 -1.60834
H   2.91113  1.58358 -1.46623
H   3.85736  0.95366 -2.81808
C  -2.83422 -0.86886  3.87030
H  -2.99345  0.21231  3.72006
H  -2.67954 -1.04386  4.95099
H  -3.76272 -1.38973  3.57708
C  -0.99699 -3.26063  3.57498
H  -1.81725 -3.96199  3.34216
H  -0.90877 -3.20624  4.67597
H  -0.06062 -3.68871  3.17948
C   0.17739 -0.43488  3.30119
H   1.12435 -0.84226  2.89729
H   0.30307 -0.40198  4.39781
H   0.06336  0.61550  2.97063
C  -2.52491 -2.07291  0.41591
C  -2.34983 -3.41461 -0.06418
C  -3.37351 -4.05136 -0.79138
H  -3.20089 -5.07766 -1.14614
C  -4.59445 -3.42324 -1.08579
C  -4.75275 -2.09726 -0.64485
H  -5.68480 -1.56327 -0.87862
C  -3.75815 -1.41739  0.07965
C  -1.05157 -4.15192  0.17122
H  -0.18795 -3.48124  0.02934
H  -0.95700 -5.01029 -0.51674
H  -0.96977 -4.54336  1.20086
C  -5.69714 -4.14492 -1.83315
H  -6.27050 -3.45529 -2.47810
H  -6.42552 -4.62007 -1.14629
H  -5.29216 -4.94646 -2.47578
C  -3.99160  0.02039  0.47922
H  -3.93670  0.16051  1.57157
H  -4.98138  0.36754  0.13613
H  -3.23462  0.70130  0.04968
Ca  0.14153  0.03195  0.22104
```

1b

SCF (BP86) Energy = -1621.11402243
Enthalpy 0K = -1620.270661
Enthalpy 298K = -1620.207916
Free Energy 298K = -1620.372580
Lowest Frequency = 10.4307 cm^{-1}
Second Frequency = 19.5926 cm^{-1}
SCF (BP86-D3BJ) Energy = -1621.38047266
SCF (C6H6) Energy = -1621.14566723
SCF (BS2) Energy = -3119.21690849

```
Ca -0.27613  0.23213 -0.55970
Si -3.12975  2.69636 -0.89815
Si  2.07314  1.68051 -2.47161
Si -1.66885 -2.79452 -1.75799
N  -2.27037  1.39971 -0.10057
N   1.73622  1.40834 -0.78640
N  -0.31859 -2.07963 -0.90413
C  -2.64114  0.85166  1.13838
C  -3.82279  0.05100  1.33611
C  -4.08064 -0.54368  2.58186
H  -4.98750 -1.15600  2.68878
```

```
C  -3.22791 -0.38903  3.69151
C  -2.09378  0.41825  3.51484
H  -1.42386  0.59340  4.36829
C  -1.79011  1.03720  2.28598
C  -4.80477 -0.16078  0.20666
H  -5.54583 -0.93479  0.47001
H  -4.29288 -0.46620 -0.72064
H  -5.36359  0.76221 -0.03564
C  -3.51592 -1.07638  5.01008
H  -3.00356 -0.57116  5.84742
H  -3.17533 -2.12992  5.01088
H  -4.59743 -1.08999  5.23637
C  -0.60818  1.98398  2.21689
H   0.29099  1.59360  1.69673
H  -0.25988  2.24335  3.23102
H  -0.87746  2.90921  1.68105
C  -4.14393  2.19275 -2.45402
H  -3.50314  1.66830 -3.18488
H  -4.55201  3.09028 -2.95480
H  -4.98717  1.52360 -2.21745
C  -1.87912  3.98408 -1.56594
H  -2.38146  4.74368 -2.19256
H  -1.10095  3.50437 -2.18539
H  -1.36559  4.50489 -0.74053
C  -4.30773  3.62656  0.29575
H  -4.84076  4.44034 -0.22789
H  -3.73784  4.07466  1.12797
H  -5.06284  2.95592  0.73942
C   2.61901  1.81558  0.23846
C   3.53405  0.87975  0.83207
C   4.37755  1.27771  1.88352
H   5.06668  0.53426  2.30870
C   4.36663  2.58230  2.41012
C   3.45553  3.48941  1.84683
H   3.40210  4.51189  2.24813
C   2.59517  3.14134  0.78776
C   3.59230 -0.54758  0.34075
H   2.64312 -1.09052  0.49983
H   4.38185 -1.11256  0.86457
H   3.78902 -0.60203 -0.74282
C   5.30904  2.99249  3.52334
H   6.31647  3.25347  3.14268
H   5.44827  2.18105  4.26020
H   4.93101  3.87670  4.06602
C   1.62946  4.17900  0.26158
H   1.37870  4.91840  1.04253
H   0.70462  3.69965 -0.09641
H   2.04362  4.73897 -0.59707
C   3.68931  0.87415 -3.12304
H   3.64504 -0.22688 -3.07596
H   4.55104  1.20060 -2.51454
H   3.88912  1.16419 -4.17095
C   2.17510  3.49984 -3.08446
H   3.03157  4.02721 -2.62925
H   1.26075  4.06643 -2.84173
H   2.31444  3.52576 -4.18132
C   0.60011  0.88718 -3.42315
H  -0.35939  1.40719 -3.23564
H   0.47758 -0.19070 -3.20132
H   0.77667  0.95990 -4.51083
C   0.64286 -2.91114 -0.28641
C   0.54485 -3.27177  1.10008
C   1.52294 -4.09246  1.69201
H   1.40989 -4.35705  2.75306
C   2.63013 -4.58422  0.97977
C   2.74254 -4.20515 -0.36899
H   3.60780 -4.55222 -0.95172
C   1.79414 -3.38280 -1.00350
C  -0.60601 -2.77466  1.94641
H  -0.51502 -3.14331  2.98266
H  -1.58404 -3.09304  1.55159
H  -0.65503 -1.67086  1.99758
C   3.68308 -5.44330  1.65008
H   4.16612 -6.12794  0.93041
H   3.25022 -6.05773  2.45950
H   4.48907 -4.83484  2.10525
C   2.02825 -2.95612 -2.43436
H   2.02172 -1.85469 -2.51676
H   1.24492 -3.32022 -3.11935
H   3.00198 -3.32601 -2.80019
C  -3.04688 -3.53282 -0.63928
H  -2.67436 -4.41141 -0.08298
H  -3.90949 -3.86665 -1.24593
H  -3.40959 -2.79518  0.09608
C  -1.22354 -4.24019 -2.94741
H  -2.13648 -4.78855 -3.24355
H  -0.54485 -4.96034 -2.45810
H  -0.73288 -3.88435 -3.86967
C  -2.46911 -1.41366 -2.81304
H  -2.67137 -0.50438 -2.21627
H  -3.43762 -1.74662 -3.22714
H  -1.81993 -1.13279 -3.66068
```

1c

SCF (BP86) Energy = -1621.11408483
Enthalpy 0K = -1620.270692
Enthalpy 298K = -1620.207989
Free Energy 298K = -1620.372126
Lowest Frequency = 9.8094 cm^{-1}
Second Frequency = 19.2929 cm^{-1}
SCF (BP86-D3BJ) Energy = -1621.38052769
SCF (C6H6) Energy = -1621.14568051
SCF (BS2) Energy = -3119.21696465

```
Ca  0.27711 -0.23031 -0.56260
N  -1.72686 -1.41996 -0.78968
Si -2.06701 -1.68687 -2.47513
Si  1.64097  2.80813 -1.76357
N   0.29935  2.08226 -0.90530
Si  3.14991 -2.67106 -0.90789
N   2.28003 -1.38389 -0.10652
C  -3.43107 -3.51898  1.83905
H  -3.37302 -4.54312  2.23548
C  -4.35998 -1.31035  1.88915
H  -5.04998 -0.57107  2.32011
C  -1.60716 -4.19487  0.24527
H  -2.02118 -4.75159 -0.61555
H  -1.35239 -4.93757  1.02177
H  -0.68462 -3.71045 -0.11194
C  -5.28087 -3.03587  3.52366
H  -6.28740 -3.30241  3.14443
H  -5.42369 -2.22683  4.26243
H  -4.89627 -3.91886  4.06374
C  -3.58570  0.52460  0.35223
H  -3.79019  0.58334 -0.72974
H  -2.63648  1.06873  0.50683
H  -4.37215  1.08623  0.88413
```

```
C  -2.15768 -3.50381 -3.09687
H  -3.01018 -4.03924 -2.64361
H  -1.23930 -4.06552 -2.85798
H  -2.29795 -3.52501 -4.19372
C  -3.69107 -0.88876 -3.11680
H  -4.54797 -1.22318 -2.50582
H  -3.89354 -1.17597 -4.16496
H  -3.65365  0.21232 -3.06543
C  -0.60254 -0.87885 -3.42740
H  -0.48662  0.19866 -3.20027
H  -0.78202 -0.94734 -4.51488
H   0.36096 -1.39337 -3.24541
C  -1.53760  4.07501  1.70905
H  -1.42562  4.32650  2.77335
C  -2.76557  4.19651 -0.34648
H  -3.63860  4.53742 -0.92118
C  -3.65890  5.48631  1.66162
H  -3.74415  5.28275  2.74367
H  -4.66287  5.36533  1.21753
H  -3.38803  6.55576  1.55626
C  -2.06224  2.95125 -2.41781
H  -1.29368  3.32919 -3.11211
H  -3.04614  3.30593 -2.77131
H  -2.03820  1.85023 -2.50151
C   2.45705  1.43121 -2.81141
H   2.66665  0.52614 -2.21075
H   3.42323  1.77201 -3.22452
H   1.81270  1.14106 -3.65962
C   3.00718  3.56933 -0.64590
H   2.61689  4.43577 -0.08281
H   3.86036  3.92572 -1.25298
H   3.38861  2.83558  0.08388
C   1.18043  4.24152 -2.96195
H   0.50136  4.96263 -2.47462
H   0.68572  3.87484 -3.87777
H   2.08870  4.79176 -3.26883
C   2.09265 -0.43330  3.51677
H   1.42535 -0.62333  4.36909
C   4.06698  0.56063  2.59053
H   4.96632  1.18317  2.70194
C   0.62652 -2.00776  2.20718
H  -0.27468 -1.62701  1.68374
H   0.27755 -2.27416  3.21926
H   0.90931 -2.92833  1.67014
C   3.49602  1.06840  5.02260
H   2.99211  0.54937  5.85662
H   3.14009  2.11682  5.03191
H   4.57750  1.09607  5.24775
C   4.79568  0.20232  0.21264
H   5.36525 -0.71276 -0.03480
H   5.52767  0.98332  0.48071
H   4.28042  0.50680 -0.71307
C   1.90959 -3.96363 -1.58560
H   1.39469 -4.48962 -0.76432
H   2.41884 -4.71866 -2.21217
H   1.13197 -3.48606 -2.20744
C   4.33149 -3.59757  0.28519
H   3.76324 -4.05207  1.11506
H   5.08102 -2.92285  0.73220
H   4.87125 -4.40596 -0.23989
C   4.16600 -2.15344 -2.45799
H   3.52449 -1.62997 -3.18888
H   4.58221 -3.04584 -2.96124
H   5.00370 -1.47960 -2.21494
C   3.21648  0.38710  3.69907
C   1.79634 -1.04674  2.28338
C   2.64428 -0.84135  1.13700
C   3.81609 -0.02802  1.34047
C  -1.81717  3.38109 -0.98970
C  -2.64555  4.57319  1.00244
C  -0.55973  3.26044  1.10832
C  -0.66003  2.91105 -0.28077
C  -3.52116 -0.90476  0.83684
C  -2.60514 -1.83495  0.23594
C  -2.57515 -3.16317  0.77898
C  -4.34327 -2.61740  2.40946
C   0.58719  2.74789  1.95080
H   0.61870  1.64344  2.00677
H   1.56811  3.04888  1.54996
H   0.50707  3.12094  2.98637
```

1d

SCF (BP86) Energy = -1621.11279532
Enthalpy 0K = -1620.268342
Enthalpy 298K = -1620.206352
Free Energy 298K = -1620.368825
Lowest Frequency = 9.1945 cm^{-1}
Second Frequency = 14.6700 cm^{-1}
SCF (BP86-D3BJ) Energy = -1621.38006573
SCF (C6H6) Energy = -1621.14338739
SCF (BS2) Energy = -3119.21530345

```
Si  0.93808  2.10263 -2.17302
Si -1.64524 -2.46481 -2.82868
N  -0.12307  2.08041 -0.77389
N  -1.54609 -1.64211 -1.29366
N   1.63922 -1.07388  1.27357
C  -0.65887  3.13970 -0.04099
C  -1.06099  2.90677  1.32603
C  -1.55172  3.93897  2.13964
H  -1.82422  3.69634  3.17669
C  -1.71174  5.25598  1.67832
C  -1.40048  5.47717  0.32753
H  -1.56350  6.47912 -0.09595
C  -0.91061  4.47544 -0.52980
C  -0.98723  1.52037  1.91575
H  -1.29833  1.52407  2.97367
H   0.03820  1.09470  1.90257
H  -1.69448  0.82029  1.41560
C  -2.18925  6.37057  2.58419
H  -2.89714  5.99988  3.34745
H  -2.70134  7.16528  2.01239
H  -1.35645  6.85629  3.13157
C  -0.74898  4.83932 -1.98804
H  -1.33174  4.16158 -2.63650
H   0.29598  4.80191 -2.34359
H  -1.11082  5.86672 -2.16710
C   0.21579  2.39490 -3.93740
H   0.86611  1.89468 -4.67842
H   0.13911  3.45508 -4.22357
H  -0.78819  1.94499 -4.02215
C   2.51451  3.17158 -1.95283
H   3.18282  2.68912 -1.21900
H   2.28410  4.18331 -1.58020
H   3.06931  3.27218 -2.90311
C   1.60374  0.29671 -2.35690
H   2.22212 -0.06814 -1.51394
```

```
H   2.28915  0.30645 -3.22222
H   0.82946 -0.45230 -2.61706
C  -2.58683 -1.69223 -0.34973
C  -2.70657 -2.78137  0.58266
C  -3.71750 -2.77368  1.56012
H  -3.78148 -3.62179  2.25646
C  -4.64141 -1.71924  1.67937
C  -4.53151 -0.65751  0.76400
H  -5.25046  0.17195  0.81925
C  -3.54233 -0.62215 -0.23727
C  -1.73984 -3.94175  0.50655
H  -1.87182 -4.52794 -0.42140
H  -1.87334 -4.62593  1.36187
H  -0.69268 -3.59002  0.49267
C  -5.69384 -1.71443  2.76930
H  -6.58247 -1.13100  2.47058
H  -5.31526 -1.26645  3.70860
H  -6.02916 -2.73780  3.01491
C  -3.52027  0.51914 -1.23189
H  -3.64559  0.14613 -2.26456
H  -2.56319  1.07428 -1.22656
H  -4.33094  1.23719 -1.02054
C  -0.12936 -3.60177 -3.12242
H   0.81420 -3.03411 -3.03876
H  -0.15423 -4.05892 -4.12868
H  -0.09072 -4.41428 -2.37708
C  -1.67250 -1.26003 -4.32516
H  -2.58285 -0.63579 -4.31252
H  -1.65303 -1.81225 -5.28325
H  -0.80617 -0.57691 -4.31673
C  -3.23558 -3.52568 -2.99058
H  -3.27756 -4.33340 -2.24056
H  -3.29441 -3.98872 -3.99203
H  -4.13601 -2.90246 -2.85304
C   2.95232 -0.87253  0.79007
C   3.62316 -1.85077 -0.02174
C   4.91685 -1.59967 -0.51810
H   5.39677 -2.36923 -1.13973
C   5.60859 -0.40590 -0.25581
C   4.93922  0.56732  0.50874
H   5.44048  1.52401  0.71347
C   3.64404  0.36834  1.01947
C   2.93618 -3.14685 -0.39222
H   1.86300 -2.98712 -0.59403
H   2.98119 -3.89271  0.42100
H   3.40356 -3.59905 -1.28407
C   7.01850 -0.17949 -0.76180
H   7.78075 -0.44144 -0.00146
H   7.18898  0.87755 -1.03327
H   7.23049 -0.79380 -1.65446
C   2.97014  1.49133  1.77697
H   2.10515  1.89350  1.21585
H   3.67235  2.32571  1.94760
H   2.58243  1.15630  2.75237
Si  1.43131 -1.87793  2.81641
C  -0.37607 -1.67559  3.39811
H  -0.58298 -0.63901  3.71198
H  -0.56182 -2.32874  4.26990
H  -1.11547 -1.95192  2.62611
C   1.79333 -3.76974  2.84752
H   1.20138 -4.31428  2.09260
H   1.54041 -4.18611  3.84069
H   2.86097 -3.98267  2.66349
C   2.55996 -1.17471  4.20652
H   3.60216 -1.07579  3.85626
H   2.56259 -1.84372  5.08641
H   2.22337 -0.17907  4.54366
Ca -0.03671 -0.25281 -0.17386
```

1e

SCF (BP86) Energy = -1621.10943405
Enthalpy 0K = -1620.264550
Enthalpy 298K = -1620.202785
Free Energy 298K = -1620.364415
Lowest Frequency = 6.0830 cm^{-1}
Second Frequency = 14.9791 cm^{-1}
SCF (BP86-D3BJ) Energy = -1621.37554214
SCF (C6H6) Energy = -1621.13987939
SCF (BS2) Energy = -3119.21217226

```
Ca  0.42591 -0.08966 -0.60519
N  -0.30605 -2.30211 -0.68239
Si  0.40388 -3.58320 -1.64087
Si  4.06059  1.11174 -1.02417
N   2.65796  0.55648 -0.13561
Si -0.82129  2.01160 -2.73560
N  -1.25248  1.59608 -1.08940
C  -2.68152 -3.31077  2.06603
H  -2.62523 -3.67473  3.10186
C  -3.96613 -2.60068  0.17039
H  -4.93498 -2.39608 -0.30653
C  -0.16800 -3.21451  2.11775
H   0.50582 -3.92991  1.62028
H  -0.34208 -3.56552  3.14983
H   0.39114 -2.26220  2.17786
C  -5.22135 -3.44855  2.21880
H  -5.57616 -4.47455  1.99626
H  -6.04016 -2.75992  1.94501
H  -5.08269 -3.38665  3.31251
C  -2.90580 -1.67990 -1.91838
H  -2.31294 -2.21143 -2.67911
H  -2.53083 -0.63780 -1.88596
H  -3.95826 -1.64974 -2.25039
C   1.05518 -5.10436 -0.65695
H   0.25259 -5.56639 -0.05597
H   1.87768 -4.83122  0.02620
H   1.43659 -5.87567 -1.35177
C  -0.77406 -4.36391 -2.94759
H  -1.73172 -4.66543 -2.48871
H  -0.31705 -5.26595 -3.39455
H  -1.00128 -3.66145 -3.76830
C   1.90073 -2.86510 -2.58738
H   1.58820 -2.19226 -3.40489
H   2.49244 -3.68000 -3.04165
H   2.57428 -2.30076 -1.92007
C   2.96168 -0.75814  3.37250
H   3.16988 -1.71673  3.86831
C   2.47834  1.59107  3.48603
H   2.28809  2.50103  4.07262
C   2.67311  0.29791  5.67292
H   1.66650  0.02713  6.04687
H   2.93975  1.26327  6.13804
H   3.37275 -0.46582  6.05673
C   2.21787  3.02365  1.42650
H   3.12950  3.42673  0.94770
H   1.87127  3.76605  2.16532
H   1.46278  2.94458  0.62405
```

C 4.57358 -0.12954 -2.39466
H 4.88605 -1.09565 -1.96242
H 5.41818 0.26776 -2.98763
H 3.74380 -0.33461 -3.09277
C 5.57975 1.33618 0.12341
H 5.37900 2.07139 0.92111
H 6.45779 1.68431 -0.44958
H 5.84833 0.38566 0.61545
C 3.82572 2.77666 -1.96053
H 3.61772 3.61059 -1.26928
H 2.98926 2.72782 -2.67860
H 4.73742 3.03041 -2.53304
C -2.82522 2.46267 2.19251
H -2.68391 2.16138 3.23990
C -3.95861 3.71733 0.51770
H -4.74155 4.42893 0.21714
C -1.04894 0.79675 1.67831
H -1.26604 -0.19212 1.21907
H -1.12616 0.63126 2.76502
H 0.01020 1.08808 1.51539
C -4.61694 4.11505 2.95095
H -4.10480 5.00497 3.36915
H -4.82513 3.44017 3.80077
H -5.58823 4.46216 2.55506
C -3.56766 3.46096 -1.92185
H -2.75679 3.93585 -2.50134
H -4.42070 4.16134 -1.93360
H -3.87407 2.55752 -2.47820
C 0.60030 0.78346 -3.21198
H 1.53332 0.87770 -2.62376
H 0.89457 1.01845 -4.24915
H 0.27218 -0.27509 -3.23189
C -0.04345 3.74966 -2.97133
H 0.80949 3.87036 -2.28215
H -0.75334 4.56783 -2.76432
H 0.33047 3.87906 -4.00350
C -2.03504 1.68450 -4.20005
H -2.63151 0.77519 -4.01329
H -1.44715 1.51277 -5.12051
H -2.73155 2.51324 -4.39867
C -3.79685 3.42727 1.88083
C -2.02241 1.85292 1.21694
C -2.12692 2.19729 -0.18189
C -3.18417 3.12891 -0.49857
C 2.47486 1.68504 2.08236
C 2.71345 0.38005 4.16060
C 2.96813 -0.71422 1.96637
C 2.71203 0.51950 1.27420
C -2.79292 -2.31955 -0.55286
C -1.50386 -2.57564 0.02684
C -1.47958 -3.04793 1.38297
C -3.94264 -3.11447 1.47843
C 3.28447 -1.96592 1.17823
H 2.41821 -2.30500 0.58013
H 4.10625 -1.79223 0.46140
H 3.57147 -2.79191 1.85190

I

SCF (BP86) Energy = -1881.67556835
Enthalpy 0K = -1880.729952
Enthalpy 298K = -1880.659957
Free Energy 298K = -1880.838634
Lowest Frequency = 18.1487 cm^{-1}
Second Frequency = 19.8250 cm^{-1}
SCF (BP86-D3BJ) Energy = -1881.99662751
SCF (C6H6) Energy = -1881.68317833
SCF (BS2) Energy = -3951.47002641

Ca 1.62776 0.91487 -0.08863
H -2.04181 -3.56145 -0.69589
Si 3.57024 -0.74569 -2.27677
Si 0.62001 3.97020 -1.08365
Si 2.68173 0.95294 3.13848
N 2.25484 -1.05623 -1.15353
N -0.03176 2.33615 -1.04368
N 1.21153 0.72298 2.20858
C 1.85527 -2.40280 -0.94832
C 2.46432 -3.22633 0.05880
C 2.05638 -4.56157 0.23491
H 2.55136 -5.15878 1.01271
C 1.04713 -5.15462 -0.54249
C 0.42289 -4.33770 -1.50067
H -0.36979 -4.76573 -2.13125
C 0.79211 -2.99341 -1.70926
C 3.52184 -2.66236 0.97858
H 3.79130 -3.39307 1.75997
H 3.15653 -1.74596 1.47339
H 4.44435 -2.38557 0.44352
C 0.67168 -6.61217 -0.37402
H 1.35290 -7.27751 -0.93795
H -0.35014 -6.81354 -0.73890
H 0.72168 -6.92640 0.68316
C 0.04340 -2.19069 -2.74802
H -0.40978 -1.27502 -2.32519
H -0.76153 -2.79443 -3.20266
H 0.70154 -1.84458 -3.56120
C 5.26573 -1.54109 -1.86658
H 5.69251 -1.15682 -0.92446
H 5.98747 -1.32321 -2.67461
H 5.18629 -2.63858 -1.78299
C 3.86246 1.15581 -2.22985
H 4.61699 1.42252 -2.99052
H 4.28087 1.49588 -1.26292
H 2.96078 1.74809 -2.47470
C 3.25379 -1.23634 -4.10191
H 4.15862 -1.05291 -4.70894
H 2.42340 -0.67007 -4.55473
H 3.01697 -2.31169 -4.18080
C -1.20216 2.03247 -1.77170
C -2.52479 2.28428 -1.26066
C -3.66847 1.90520 -1.99896
H -4.65965 2.13105 -1.57886
C -3.58653 1.29516 -3.26011
C -2.29385 1.06135 -3.76966
H -2.18699 0.60367 -4.76239
C -1.12691 1.41457 -3.07213
C -2.73245 2.98897 0.06656
H -1.88733 2.82166 0.75377
H -3.67865 2.67961 0.55116
H -2.80231 4.08307 -0.05966
C -4.82420 0.94234 -4.05957
H -4.90081 1.55189 -4.97887
H -5.74471 1.11811 -3.47694
H -4.82457 -0.11454 -4.38339
C 0.21711 1.17639 -3.71662
H 0.10076 0.67616 -4.69262
H 0.86581 0.54413 -3.08480

```
H   0.75635  2.12451 -3.88046
C  -0.42369  5.38632 -0.32042
H  -0.71671  5.18774  0.72307
H  -1.33843  5.57770 -0.90732
H   0.17137  6.31761 -0.33376
C   1.06545  4.60470 -2.83496
H   0.19549  4.54015 -3.51145
H   1.89025  4.02942 -3.28886
H   1.37665  5.66402 -2.79609
C   2.24834  3.91922 -0.06400
H   3.06139  3.35771 -0.56105
H   2.09997  3.53104  0.96119
H   2.62827  4.94952  0.04941
C  -0.04019  0.49462  2.79616
C  -0.53859 -0.84512  2.99880
C  -1.82149 -1.05789  3.54705
H  -2.15061 -2.09273  3.71400
C  -2.68095  0.00023  3.90755
C  -2.20424  1.30792  3.68870
H  -2.84145  2.15988  3.96338
C  -0.92216  1.57387  3.16578
C   0.30741 -2.03980  2.62630
H  -0.23520 -2.98199  2.81072
H   1.24229 -2.06735  3.20899
H   0.62098 -2.03017  1.56730
C  -4.02473 -0.25473  4.55985
H  -4.74477  0.55363  4.34436
H  -3.93216 -0.31638  5.66048
H  -4.46971 -1.20704  4.22305
C  -0.47556  3.00862  3.01225
H   0.02911  3.16374  2.04417
H   0.25228  3.28850  3.79265
H  -1.32954  3.70251  3.08940
C   3.11712 -0.47076  4.34150
H   2.27824 -0.67045  5.03109
H   3.99290 -0.19866  4.95759
H   3.35436 -1.40831  3.81257
C   2.78454  2.53827  4.21230
H   3.78540  2.60853  4.67612
H   2.04583  2.52443  5.03252
H   2.62345  3.45608  3.62199
C   4.09339  1.11147  1.84410
H   4.12087  0.27377  1.12216
H   5.06479  1.09391  2.36882
H   4.06204  2.07217  1.29735
C  -3.00731 -3.10714 -0.44396
C  -3.64174 -2.24079 -1.35580
C  -3.61650 -3.38615  0.79449
C  -4.87913 -1.65342 -1.02799
H  -3.17159 -2.01958 -2.31958
C  -4.85382 -2.80205  1.12106
H  -3.12895 -4.06786  1.49880
C  -5.48477 -1.93413  0.21079
H  -5.36890 -0.98412 -1.74198
H  -5.33225 -3.03178  2.07893
H  -6.45451 -1.48986  0.46003
K  -2.40692 -0.05381  0.65216
```

I′

SCF (BP86) Energy = -1853.36027721
Enthalpy 0K = -1852.418262
Enthalpy 298K = -1852.348757
Free Energy 298K = -1852.533957
Lowest Frequency = 7.5040 cm^{-1}
Second Frequency = 12.8154 cm^{-1}
SCF (BP86-D3BJ) Energy = -1853.65204267
SCF (C6H6) Energy = -1853.39208963
SCF (BS2) Energy = -3351.51972237

```
Ca  1.57211  0.10208  0.37086
Si  2.42365 -2.74330  2.17661
Si  1.83302  2.90408  2.40282
Si  3.69870  0.43936 -2.36952
N   1.08271 -1.71073  1.75707
N   0.95582  2.25429  1.04163
N   2.20939 -0.29575 -1.83916
C  -0.24576 -2.01994  2.12649
C  -1.12066 -2.71594  1.22309
C  -2.44800 -2.99792  1.59461
H  -3.07943 -3.55407  0.88669
C  -2.98682 -2.60714  2.83471
C  -2.13953 -1.90070  3.70319
H  -2.53204 -1.56121  4.67218
C  -0.80322 -1.59834  3.37896
C  -0.62979 -3.15287 -0.13844
H  -0.31559 -2.30383 -0.77279
H  -1.42095 -3.69064 -0.68776
H   0.25061 -3.81192 -0.06766
C  -4.42705 -2.90577  3.19833
H  -4.68919 -3.96249  3.00437
H  -5.13673 -2.28786  2.61542
H  -4.62034 -2.70517  4.26629
C   0.02261 -0.78731  4.35056
H  -0.60649 -0.39617  5.16891
H   0.50035  0.06504  3.83723
H   0.84027 -1.37314  4.80362
C   2.85021 -2.91233  4.04598
H   3.73711 -3.56026  4.17659
H   2.02027 -3.37154  4.61115
H   3.07589 -1.93506  4.50601
C   3.97928 -1.97095  1.36019
H   3.83807 -1.79006  0.27795
H   4.83952 -2.65679  1.45869
H   4.26917 -1.02289  1.84880
C   2.28043 -4.56237  1.57525
H   2.31649 -4.63526  0.47531
H   1.32811 -5.00880  1.91143
H   3.10197 -5.17901  1.98404
C  -0.13191  2.95942  0.47731
C  -1.48102  2.69228  0.88811
C  -2.55331  3.39947  0.31026
H  -3.57126  3.18499  0.66560
C  -2.36897  4.36795 -0.69148
C  -1.05161  4.59362 -1.12696
H  -0.87073  5.31961 -1.93244
C   0.05190  3.91731 -0.57680
C  -1.77388  1.64286  1.93620
H  -1.29038  1.87251  2.89995
H  -1.40438  0.64129  1.64987
H  -2.85949  1.55282  2.10984
C  -3.53428  5.14642 -1.26703
H  -3.43223  5.28838 -2.35807
H  -3.62093  6.15663 -0.82051
H  -4.49289  4.63051 -1.08412
C   1.42964  4.17781 -1.14043
H   1.92974  3.22880 -1.40031
H   2.09131  4.68844 -0.42064
```

```
H   1.37043  4.80307 -2.04820
C   2.84183  4.51569  2.10356
H   3.59935  4.38525  1.31186
H   3.36869  4.81298  3.02953
H   2.18602  5.35502  1.81297
C   0.76674  3.31277  3.94746
H   1.36561  3.84128  4.71178
H   0.35091  2.40280  4.41152
H  -0.07871  3.96760  3.67267
C   3.12020  1.57372  2.90857
H   3.61002  1.86184  3.85573
H   3.92159  1.47111  2.15422
H   2.65491  0.58459  3.07824
C   1.35509 -0.97689 -2.73649
C   0.22901 -0.31083 -3.32938
C  -0.63288 -1.00516 -4.19767
H  -1.48169 -0.46137 -4.63550
C  -0.45060 -2.36162 -4.51806
C   0.62942 -3.02211 -3.90883
H   0.78384 -4.09183 -4.11011
C   1.51872 -2.37131 -3.03386
C  -0.05450  1.14222 -3.02139
H  -0.25906  1.32326 -1.94969
H  -0.93693  1.49679 -3.58041
H   0.79884  1.79233 -3.27471
C  -1.37074 -3.07545 -5.48703
H  -2.40364 -2.68966 -5.42509
H  -1.40525 -4.16077 -5.28649
H  -1.04477 -2.95236 -6.53895
C   2.61478 -3.17420 -2.37169
H   2.46841 -4.25450 -2.54528
H   2.62806 -2.99165 -1.28342
H   3.61948 -2.90773 -2.74161
C   3.51125  1.71012 -3.79782
H   4.49897  2.03361 -4.17450
H   2.95904  2.60941 -3.47729
H   2.96079  1.26119 -4.64324
C   5.09833 -0.73347 -2.97851
H   5.37869 -1.47211 -2.20824
H   6.00179 -0.14666 -3.22947
H   4.80045 -1.28481 -3.88750
C   4.42500  1.35173 -0.84575
H   4.77778  0.64054 -0.07650
H   3.69607  2.04583 -0.38736
H   5.29897  1.95739 -1.14473
C  -7.57270  0.27607 -1.31854
C  -6.41376  1.06908 -1.26763
C  -7.52468 -1.06293 -0.89630
H  -8.51296  0.70129 -1.68804
C  -5.20700  0.52647 -0.79459
H  -6.44963  2.11295 -1.59865
C  -6.31762 -1.60635 -0.42463
H  -8.42741 -1.68332 -0.93617
C  -5.15850 -0.81325 -0.37197
H  -4.30508  1.14666 -0.75314
H  -6.27759 -2.65131 -0.09794
H  -4.21851 -1.23897 -0.00404
```

II

SCF (BP86) Energy = -1650.05123818
Enthalpy 0K = -1649.201425
Enthalpy 298K = -1649.137043
Free Energy 298K = -1649.302095
Lowest Frequency = 14.8529 cm^{-1}
Second Frequency = 20.3983 cm^{-1}
SCF (BP86-D3BJ) Energy = -1650.32972328
SCF (C6H6) Energy = -1650.08736532
SCF (BS2) Energy = -3719.80354974

```
Ca  0.27008 -0.21655 -0.46606
H  -0.08439 -0.02769  1.62471
Si  3.16550 -2.25452 -1.37256
Si -2.42718 -1.93799 -2.44304
Si  0.58571  2.85141 -2.49331
N   2.51237 -1.02200 -0.32642
N  -1.84606 -1.49769 -0.85906
N  -0.12283  2.11400 -1.08240
C   3.31705 -0.55318  0.74171
C   4.19576  0.57183  0.57987
C   5.00174  1.01513  1.64489
H   5.66473  1.87608  1.47588
C   4.98094  0.40509  2.90990
C   4.08189 -0.66081  3.08680
H   4.01031 -1.14078  4.07369
C   3.25531 -1.13808  2.05342
C   4.22613  1.33589 -0.72323
H   4.93413  2.18167 -0.66638
H   3.22614  1.74017 -0.95953
H   4.51280  0.70425 -1.57949
C   5.89429  0.86544  4.02754
H   6.85350  0.30978  4.04184
H   5.42801  0.72103  5.01873
H   6.14713  1.93632  3.92918
C   2.25737 -2.22859  2.36249
H   1.22890 -1.83607  2.24063
H   2.37330 -2.58098  3.40322
H   2.35868 -3.09601  1.69104
C   4.82742 -1.82750 -2.24906
H   4.70313 -1.02089 -2.99229
H   5.22832 -2.71259 -2.77715
H   5.58883 -1.50086 -1.51930
C   1.88693 -2.57990 -2.75745
H   2.18925 -3.44259 -3.37769
H   1.79442 -1.70627 -3.42617
H   0.88949 -2.80870 -2.34156
C   3.52615 -3.95545 -0.54292
H   3.99602 -4.65039 -1.26385
H   2.60303 -4.43061 -0.16878
H   4.21694 -3.84236  0.31090
C  -2.40273 -2.02404  0.31342
C  -3.59322 -1.48968  0.92780
C  -4.04490 -1.97227  2.17650
H  -4.96016 -1.53597  2.60769
C  -3.39384 -3.00642  2.86823
C  -2.25028 -3.55718  2.25490
H  -1.71910 -4.37735  2.75804
C  -1.75568 -3.10175  1.02404
C  -4.36962 -0.37067  0.26429
H  -3.70117  0.27044 -0.33358
H  -4.91586  0.24436  1.00685
H  -5.13143 -0.75082 -0.43843
C  -3.91021 -3.53075  4.19257
H  -4.29627 -4.56499  4.10652
H  -4.73639 -2.90678  4.57666
H  -3.12055 -3.55296  4.96638
C  -0.52910 -3.75483  0.43303
H  -0.19948 -4.60533  1.05435
```

```
H   0.32236 -3.05333  0.36000
H  -0.72199 -4.11983 -0.58953
C  -4.29729 -1.65574 -2.82026
H  -4.58220 -0.59331 -2.73976
H  -4.94615 -2.23946 -2.14386
H  -4.51657 -1.98650 -3.85261
C  -2.17224 -3.79274 -2.89330
H  -2.65922 -4.45135 -2.15226
H  -1.10163 -4.05797 -2.92886
H  -2.60926 -4.02421 -3.88256
C  -1.47943 -0.90336 -3.73788
H  -0.41625 -1.19180 -3.79036
H  -1.53515  0.17381 -3.50432
H  -1.91184 -1.05515 -4.74302
C  -0.88027  2.79490 -0.13188
C  -0.29026  3.25606  1.10639
C  -1.08356  3.87117  2.09407
H  -0.59031  4.22236  3.01230
C  -2.47394  4.06826  1.94626
C  -3.05559  3.61136  0.74633
H  -4.13525  3.75229  0.58858
C  -2.30275  3.00258 -0.27927
C   1.18243  3.04804  1.36099
H   1.49917  3.55379  2.28968
H   1.79482  3.42501  0.52701
H   1.41258  1.97097  1.46826
C  -3.28816  4.79999  2.99564
H  -4.34767  4.48648  2.98434
H  -3.27966  5.89569  2.83284
H  -2.89521  4.63040  4.01454
C  -3.00350  2.55515 -1.54109
H  -2.61934  1.57497 -1.87211
H  -2.82359  3.25549 -2.37504
H  -4.09526  2.48935 -1.39151
C   1.78889  4.29858 -2.09372
H   1.29422  5.05383 -1.45729
H   2.11692  4.80578 -3.01998
H   2.69015  3.94921 -1.56270
C  -0.61228  3.62636 -3.79053
H  -0.02995  4.00320 -4.65240
H  -1.17541  4.48168 -3.37677
H  -1.33873  2.88970 -4.17368
C   1.54712  1.49613 -3.43720
H   2.14930  0.86704 -2.75820
H   2.24051  1.95444 -4.16505
H   0.85777  0.84375 -3.99991
K  -2.18713  0.86487  2.25544
```

III

SCF (BP86) Energy = -1881.09226235
Enthalpy 0K = -1880.160618
Enthalpy 298K = -1880.091023
Free Energy 298K = -1880.267129
Lowest Frequency = 15.6796 cm^{-1}
Second Frequency = 19.3673 cm^{-1}
SCF (BP86-D3BJ) Energy = -1881.40980284
SCF (C6H6) Energy = -1881.12743619
SCF (BS2) Energy = -3950.90001061

```
Ca -0.26457  0.21304 -0.52695
Si -3.36955  1.72411 -1.93116
Si  2.23089  2.15978 -2.59629
Si -0.19834 -2.79999 -2.79656
N  -2.58308  0.76089 -0.70175
N   1.51732  1.81210 -1.03373
N   0.41235 -2.10947 -1.30426
C  -3.37639  0.29026  0.37411
C  -4.00261 -1.00220  0.33702
C  -4.78678 -1.45322  1.41579
H  -5.25411 -2.44621  1.34387
C  -4.99046 -0.68338  2.57263
C  -4.34174  0.56248  2.63052
H  -4.44628  1.17721  3.53594
C  -3.54349  1.05280  1.58205
C  -3.80266 -1.91727 -0.84953
H  -4.29466 -2.89145 -0.67983
H  -2.72969 -2.10191 -1.03632
H  -4.20384 -1.48972 -1.78327
C  -5.87969 -1.16581  3.70039
H  -6.91462 -0.77844  3.61307
H  -5.50018 -0.84019  4.68560
H  -5.95056 -2.26798  3.71597
C  -2.81314  2.36117  1.77463
H  -1.72067  2.19160  1.78422
H  -3.09031  2.82499  2.73758
H  -3.01757  3.08325  0.96968
C  -4.79898  0.84441 -2.87873
H  -4.42902 -0.00558 -3.47782
H  -5.30050  1.54900 -3.56817
H  -5.56358  0.46177 -2.17995
C  -2.07206  2.22364 -3.24263
H  -2.46498  3.02938 -3.88833
H  -1.81198  1.37079 -3.89159
H  -1.14673  2.59408 -2.76894
C  -4.19612  3.35605 -1.32457
H  -4.76065  3.82708 -2.15094
H  -3.45144  4.08804 -0.96743
H  -4.90653  3.16559 -0.50134
C   1.90894  2.67002  0.01727
C   3.07133  2.41476  0.82472
C   3.44675  3.29927  1.85488
H   4.34845  3.07018  2.44264
C   2.70621  4.44994  2.16562
C   1.53533  4.67454  1.41676
H   0.91047  5.54737  1.65331
C   1.12480  3.82400  0.37666
C   3.88117  1.15271  0.62375
H   3.23589  0.35250  0.22196
H   4.35657  0.83539  1.57321
H   4.69430  1.27027 -0.11503
C   3.14363  5.40907  3.25342
H   3.60619  6.32643  2.83879
H   3.88906  4.94526  3.92304
H   2.29199  5.73684  3.87664
C  -0.16323  4.12723 -0.35391
H  -0.86501  3.27432 -0.31981
H   0.00891  4.34311 -1.42145
H  -0.66988  4.99885  0.09547
C   4.15373  2.03099 -2.67292
H   4.51050  1.01028 -2.45449
H   4.62842  2.72214 -1.95439
H   4.51211  2.30510 -3.68260
C   1.89265  3.93155 -3.26913
H   2.17494  4.70192 -2.53038
H   0.82758  4.07375 -3.52047
H   2.48165  4.11646 -4.18666
C   1.54534  0.93970 -3.89324
```

```
H   0.51125  1.20191 -4.17175
H   1.54929 -0.09556 -3.51382
H   2.15848  0.96873 -4.81172
C   1.29895 -2.85399 -0.53093
C   0.85754 -3.79578  0.47940
C   1.79001 -4.50822  1.26250
H   1.40592 -5.22324  2.00523
C   3.18510 -4.35982  1.11706
C   3.62483 -3.42896  0.15111
H   4.70513 -3.28869 -0.00229
C   2.73419 -2.68947 -0.65397
C  -0.61669 -4.03935  0.70651
H  -0.82925 -4.26778  1.76504
H  -0.98101 -4.89163  0.10534
H  -1.20487 -3.16241  0.39650
C   4.16243 -5.20301  1.91268
H   5.12436 -4.68196  2.06752
H   4.39702 -6.15397  1.39604
H   3.75909 -5.47354  2.90510
C   3.28924 -1.72389 -1.67315
H   2.79057 -0.74094 -1.60918
H   3.11861 -2.08626 -2.70094
H   4.37582 -1.58316 -1.53744
C  -1.20690 -4.42978 -2.60977
H  -0.59503 -5.23663 -2.16908
H  -1.53948 -4.77538 -3.60629
H  -2.10293 -4.29546 -1.98205
C   1.16701 -3.28925 -4.06424
H   0.71542 -3.80787 -4.93033
H   1.90872 -3.97590 -3.61874
H   1.70405 -2.40494 -4.44794
C  -1.32701 -1.52642 -3.65373
H  -1.96311 -0.98940 -2.92842
H  -1.99415 -2.03334 -4.37357
H  -0.73242 -0.78467 -4.21161
C  -0.05949  0.01363  2.04063
C   0.71516  0.84602  2.90981
C  -0.72302 -1.05159  2.72564
C   0.87452  0.60929  4.29476
H   1.23473  1.71887  2.49211
C  -0.57693 -1.31952  4.10491
H  -1.39877 -1.71109  2.16395
C   0.24473 -0.49611  4.89823
H   1.47901  1.29531  4.90517
H  -1.11695 -2.15840  4.56580
H   0.35998 -0.68942  5.97239
K   2.27906 -1.60020  2.27255
```

IV

SCF (BP86) Energy = -1648.83267281
Enthalpy 0K = -1648.000604
Enthalpy 298K = -1647.937455
Free Energy 298K = -1648.098837
Lowest Frequency = 14.8219 cm^{-1}
Second Frequency = 22.0863 cm^{-1}
SCF (BP86-D3BJ) Energy = -1649.11158974
SCF (C6H6) Energy = -1648.86656874
SCF (BS2) Energy = -3718.58879882

```
Ca -0.40188  0.53084  0.34175
K   3.56643 -1.23995  0.59664
Si  0.64570 -1.40343  2.73804
Si -3.48043  2.21433  1.14182
Si  0.83149  3.62081 -1.26924
N   0.69945 -1.47045  0.98978
N  -2.75766  0.84679  0.31670
N   1.21492  2.05705 -0.54765
C   2.26583 -0.95309  3.70469
H   3.05799 -1.71499  3.61669
H   2.02755 -0.82370  4.77767
H   2.66817  0.01517  3.35058
C  -0.38830  0.18708  3.16293
H   0.13632  1.13470  2.92064
H  -0.51204  0.21031  4.26006
H  -1.41066  0.21571  2.73961
C   0.95872 -2.58880  0.13404
C   0.31822 -2.62630 -1.14245
C   0.60142 -3.61842 -2.10746
H   0.06781 -3.59813 -3.06668
C   1.52246 -4.64619 -1.82647
C   2.13801 -4.64529 -0.57383
H   2.85491 -5.44587 -0.33744
C  -0.73040 -1.60045 -1.50905
H  -1.48950 -1.47272 -0.71262
H  -1.29298 -1.90623 -2.40640
H  -0.30184 -0.60562 -1.78373
C   1.78789 -5.75137 -2.83060
H   1.87786 -5.35635 -3.85975
H   0.97227 -6.50145 -2.85037
H   2.72130 -6.29332 -2.59618
C  -3.66097 -0.08865 -0.25619
C  -4.12379 -1.23267  0.47786
C  -5.02864 -2.13899 -0.10465
H  -5.36168 -2.99967  0.49217
C  -5.50398 -1.99271 -1.41854
C  -5.00239 -0.90935 -2.15911
H  -5.31601 -0.78651 -3.20561
C  -4.09785  0.02323 -1.61898
C  -3.58814 -1.52585  1.86031
H  -2.50033 -1.72113  1.82203
H  -4.07304 -2.42037  2.28784
H  -3.73247 -0.68637  2.55867
C  -6.51295 -2.95658 -2.00775
H  -7.55624 -2.64572 -1.80084
H  -6.39292 -3.97277 -1.59261
H  -6.41108 -3.02824 -3.10497
C  -3.54119  1.10152 -2.51971
H  -3.95337  1.01049 -3.53981
H  -2.44069  1.02960 -2.58942
H  -3.75390  2.11771 -2.15172
C  -4.51271  3.40374  0.03635
H  -3.89269  3.90652 -0.72507
H  -4.98822  4.18691  0.65549
H  -5.31768  2.85827 -0.48631
C  -2.07628  3.25887  1.92340
H  -1.65574  2.77309  2.82015
H  -2.47003  4.24334  2.23330
H  -1.25152  3.45478  1.21552
C  -4.69072  1.77566  2.57193
H  -5.46286  1.06362  2.23250
H  -5.20806  2.68363  2.93283
H  -4.16871  1.32321  3.43276
C   2.57686  1.70951 -0.55220
C   3.45239  2.04584  0.54252
C   4.82535  1.73111  0.49154
H   5.46096  2.02117  1.34033
C   5.41280  1.08152 -0.61279
```

```
C   4.55055  0.70570 -1.66267
H   4.96907  0.18111 -2.53348
C   3.16804  0.98228 -1.64980
C   2.89996  2.73863  1.76782
H   2.03226  2.19316  2.18035
H   2.53327  3.75278  1.53919
H   3.66685  2.81853  2.55735
C   6.90623  0.83321 -0.68441
H   7.34490  0.69299  0.31917
H   7.43896  1.68369 -1.15247
H   7.14295 -0.06271 -1.28473
C   2.31167  0.47344 -2.78968
H   1.84243 -0.49751 -2.53605
H   2.91607  0.31229 -3.69884
H   1.49405  1.17250 -3.01942
C  -0.94683  3.56943 -1.95065
H  -0.97906  3.01446 -2.90401
H  -1.32991  4.58826 -2.13882
H  -1.64437  3.06686 -1.25885
C   2.00183  4.09088 -2.71611
H   3.05905  4.08115 -2.40071
H   1.76592  5.11029 -3.07170
H   1.90244  3.40423 -3.57332
C   0.93300  5.13962 -0.08759
H   0.37315  4.98444  0.85030
H   0.51015  6.03190 -0.58590
H   1.97879  5.37779  0.17548
C  -0.21213 -2.85109  3.64843
H   0.34704 -3.78585  3.47970
H  -0.27440 -2.66463  4.73599
H  -1.23572 -2.99621  3.26457
C   1.91600 -3.65923  0.44672
C   2.71998 -3.71380  1.64012
H   3.23087 -4.67280  1.80961
H   2.30879 -3.30576  2.56793
```

TS(IV-V)

```
SCF (BP86) Energy = -1648.82542721
Enthalpy 0K = -1647.994147
Enthalpy 298K = -1647.931740
Free Energy 298K = -1648.091700
```

Lowest Frequency = -199.4374 cm^{-1}
Second Frequency = 15.1270 cm^{-1}

```
SCF (BP86-D3BJ) Energy =
                   -1649.105961
SCF (C6H6) Energy = -1648.85908093
SCF (BS2) Energy = -3718.58081208
```

```
Ca  0.52905 -0.56947  0.35107
K  -3.47320  1.24211  0.62415
Si -0.81527  1.32357  2.71427
Si  3.70254 -2.05401  1.11332
Si -0.69040 -3.60085 -1.29987
N  -0.48389  1.43687  0.96878
N   2.87626 -0.76984  0.25867
N  -1.14430 -2.06999 -0.56024
C  -2.44915  0.68485  3.57667
H  -3.15167  1.51869  3.73157
H  -2.19921  0.22791  4.55503
H  -2.95047 -0.11245  2.99218
C   0.22298 -0.33308  3.08538
H  -0.21249 -1.28105  2.69948
H   0.22587 -0.47066  4.18126
H   1.29213 -0.27885  2.79427
C  -0.88941  2.46721  0.10161
C  -0.49186  2.44827 -1.26952
C  -1.07490  3.32894 -2.21132
H  -0.73447  3.28721 -3.25536
C  -2.06447  4.25409 -1.83677
C  -2.40629  4.32869 -0.47127
H  -3.13045  5.09125 -0.14489
C   0.54519  1.45978 -1.75540
H   1.44160  1.43907 -1.10502
H   0.90552  1.72102 -2.76494
H   0.14362  0.42475 -1.86597
C  -2.66670  5.21070 -2.84751
H  -2.55682  4.83093 -3.87898
H  -2.18544  6.20896 -2.81820
H  -3.74494  5.37669 -2.66487
C   3.67239  0.26809 -0.29189
C   3.95013  1.47088  0.44300
C   4.74991  2.48167 -0.12153
H   4.94444  3.38498  0.47355
C   5.29202  2.38464 -1.41428
C   4.96926  1.23588 -2.15597
H   5.33871  1.14402 -3.18745
C   4.17445  0.19832 -1.63532
C   3.33300  1.70211  1.80368
H   2.23161  1.78537  1.72652
H   3.70705  2.64036  2.24854
H   3.54195  0.87884  2.50523
C   6.18821  3.46710 -1.97978
H   7.25278  3.30699 -1.71675
H   5.91030  4.46478 -1.59641
H   6.13218  3.50376 -3.08211
C   3.79762 -0.95413 -2.53830
H   4.24920 -0.83134 -3.53833
H   2.70162 -1.01562 -2.66393
H   4.10965 -1.93123 -2.13671
C   4.89988 -3.13129  0.05947
H   4.36468 -3.69229 -0.72594
H   5.42431 -3.86463  0.69987
H   5.66795 -2.50840 -0.43118
C   2.38387 -3.24936  1.82018
H   1.73869 -2.75899  2.57003
H   2.87990 -4.09961  2.32179
H   1.74239 -3.67117  1.02748
C   4.78598 -1.48532  2.59746
H   5.49036 -0.69207  2.29242
H   5.38093 -2.32911  2.99295
H   4.17172 -1.09004  3.42485
C  -2.50686 -1.73917 -0.53834
C  -3.35634 -2.08670  0.57717
C  -4.72338 -1.74481  0.58295
H  -5.33194 -2.04095  1.44934
C  -5.34180 -1.07090 -0.49043
C  -4.51577 -0.71472 -1.57461
H  -4.96052 -0.19043 -2.43303
C  -3.13801 -1.01625 -1.61896
C  -2.78655 -2.84315  1.75509
H  -1.91014 -2.32775  2.18326
H  -2.43295 -3.84507  1.46059
H  -3.54080 -2.96230  2.55160
C  -6.83249 -0.79483 -0.50053
H  -7.22408 -0.62135  0.51766
H  -7.40277 -1.64606 -0.92042
H  -7.08064  0.08883 -1.11465
C  -2.34046 -0.54676 -2.81732
```

```
H  -2.03803  0.51319 -2.70882
H  -2.93518 -0.62253 -3.74454
H  -1.42120 -1.13917 -2.93003
C   1.07536 -3.43729 -2.00352
H   1.05905 -2.86021 -2.94456
H   1.51031 -4.42852 -2.22412
H   1.76434 -2.91443 -1.31599
C  -1.85360 -4.11792 -2.73636
H  -2.90567 -4.15786 -2.40544
H  -1.57845 -5.12296 -3.10426
H  -1.79737 -3.41927 -3.58772
C  -0.68562 -5.13113 -0.12870
H  -0.14175 -4.93713  0.81148
H  -0.19913 -5.99046 -0.62688
H  -1.71229 -5.44351  0.13271
C   0.08400  2.52583  3.92413
H   0.62999  3.30197  3.36295
H  -0.63400  3.03449  4.59020
H   0.81594  1.98477  4.54979
C  -1.81329  3.51550  0.53275
C  -2.22806  3.52756  1.92880
H  -3.07505  4.21581  2.12468
H  -1.41921  3.79113  2.62316
```

V

SCF (BP86) Energy = -1648.83760232
Enthalpy 0K = -1648.006922
Enthalpy 298K = -1647.943516
Free Energy 298K = -1648.107163
Lowest Frequency = 11.2106 cm^{-1}
Second Frequency = 15.5760 cm^{-1}
SCF (BP86-D3BJ) Energy = -1649.11651963
SCF (C6H6) Energy = -1648.87212122
SCF (BS2) Energy = -3718.59136147

```
Ca  0.62600 -0.53941  0.60643
K  -3.24728  0.48225  0.16947
Si  4.25416 -0.98552  1.04215
Si  0.15601 -4.07782 -0.36568
N  -0.69424  1.27737  1.11690
N   2.91620 -0.21877  0.22252
N  -0.56987 -2.47954 -0.39491
C  -1.88150  3.01385  2.82469
H  -2.81081  3.20245  3.39946
H  -1.11857  3.70015  3.24967
C  -2.81841  0.24777  3.35251
H  -3.69077  0.85502  3.03380
H  -2.90352  0.16572  4.45284
H  -2.91643 -0.78145  2.95812
C   0.00325  1.61032  4.46856
H   0.45183  0.77114  5.02701
H  -0.58618  2.22986  5.16943
H   0.83049  2.24962  4.10676
C  -1.26484  2.40269  0.53546
C  -1.25186  2.64539 -0.87938
C  -2.04562  3.67915 -1.42201
H  -2.01917  3.83860 -2.51039
C  -2.85491  4.51293 -0.62847
C  -2.81694  4.30916  0.77402
H  -3.40463  4.97122  1.42752
C  -0.41009  1.77007 -1.77374
H   0.66330  1.82760 -1.50983
H  -0.50686  2.05271 -2.83623
H  -0.70337  0.70158 -1.70073
C  -3.67850  5.62879 -1.23951
H  -4.71155  5.65054 -0.84288
H  -3.74666  5.52299 -2.33683
H  -3.24566  6.62918 -1.03792
C   3.16146  0.86400 -0.65553
C   3.12893  2.22670 -0.19547
C   3.35607  3.28420 -1.09528
H   3.32514  4.31236 -0.70843
C   3.60119  3.07588 -2.46412
C   3.58286  1.74786 -2.92241
H   3.73633  1.54930 -3.99299
C   3.36477  0.65539 -2.06246
C   2.78620  2.54598  1.24265
H   1.73781  2.26623  1.46649
H   2.89277  3.62714  1.43757
H   3.42116  1.99756  1.95524
C   3.88075  4.23327 -3.40085
H   4.94808  4.53116 -3.38865
H   3.29594  5.12893 -3.12572
H   3.63051  3.97676 -4.44538
C   3.30255 -0.73761 -2.64813
H   3.33233 -0.69990 -3.75129
H   2.38128 -1.26374 -2.34160
H   4.13550 -1.37532 -2.30712
C   5.56975 -1.79643 -0.10662
H   5.14230 -2.63073 -0.68910
H   6.41177 -2.19666  0.48826
H   5.98551 -1.06187 -0.81849
C   3.57571 -2.38553  2.15398
H   2.83539 -2.01411  2.88436
H   4.40174 -2.84320  2.72776
H   3.10257 -3.18728  1.56244
C   5.29461  0.16254  2.18382
H   5.65090  1.05417  1.63915
H   6.18319 -0.37341  2.56620
H   4.71098  0.50963  3.05392
C  -1.87083 -2.29158 -0.84702
C  -3.04327 -2.60997 -0.06113
C  -4.33705 -2.30755 -0.53734
H  -5.20039 -2.57354  0.09086
C  -4.56517 -1.69411 -1.78642
C  -3.42807 -1.42485 -2.57887
H  -3.56759 -0.99725 -3.58204
C  -2.12093 -1.73481 -2.15929
C  -2.89270 -3.28814  1.28093
H  -2.09972 -2.81761  1.88529
H  -2.59962 -4.34761  1.16363
H  -3.83899 -3.26819  1.84881
C  -5.96724 -1.41543 -2.29256
H  -6.69667 -1.37278 -1.46459
H  -6.31668 -2.20389 -2.98713
H  -6.02631 -0.45998 -2.84443
C  -0.96962 -1.57895 -3.12624
H  -0.10120 -1.07348 -2.67547
H  -1.27422 -1.02489 -4.03022
H  -0.60126 -2.57390 -3.43983
C   1.88619 -4.01051 -1.17467
H   1.80181 -3.85963 -2.26482
H   2.45809 -4.93968 -0.99895
H   2.47311 -3.16551 -0.77406
C  -0.89513 -5.34913 -1.34580
H  -1.91097 -5.45579 -0.92803
H  -0.41620 -6.34451 -1.32810
```

```
H  -1.00252 -5.04505 -2.40144
C   0.42836 -4.80493  1.39126
H   0.96330 -4.09043  2.04055
H   1.03446 -5.72834  1.34221
H  -0.52423 -5.05123  1.88967
C  -2.03248  3.30850  1.36319
Si -1.09780  1.12054  2.92657
C  -0.14681 -0.83725  3.03638
H  -0.43382 -1.71376  2.40477
H   0.96715 -0.82392  3.11538
H  -0.47354 -1.13774  4.04796
```

TS(V-VI)

SCF (BP86) Energy = -1648.83663425
Enthalpy 0K = -1648.006454
Enthalpy 298K = -1647.943525
Free Energy 298K = -1648.105610
Lowest Frequency = -108.0536 cm^{-1}
Second Frequency = 11.0544 cm^{-1}
SCF (BP86-D3BJ) Energy = -1649.11478904
SCF (C6H6) Energy = -1648.87110448
SCF (BS2) Energy = -3718.58958988

```
Ca  0.55344 -0.62019  0.71158
K  -3.19577  0.68679  0.10566
Si  4.15233 -1.28025  1.02729
Si -0.13950 -4.08655 -0.47655
N  -0.64884  1.33013  1.11071
N   2.86366 -0.41219  0.22982
N  -0.76165 -2.44706 -0.40973
C  -1.74327  3.18240  2.78853
H  -2.71220  3.36193  3.29645
H  -1.01946  3.86809  3.27624
C  -2.69437  0.39630  3.38117
H  -3.58393  1.00319  3.11288
H  -2.73444  0.26847  4.47893
H  -2.78168 -0.61284  2.94496
C   0.19520  1.63799  4.34372
H   0.56020  0.74507  4.87184
H  -0.27843  2.32215  5.07212
H   1.07096  2.16921  3.92879
C  -1.10319  2.50374  0.51277
C  -1.02547  2.73560 -0.90244
C  -1.67821  3.85310 -1.46346
H  -1.60451  4.00413 -2.55060
C  -2.40213  4.78267 -0.69312
C  -2.43614  4.57514  0.70691
H  -2.96673  5.29834  1.34395
C  -0.25296  1.77745 -1.77464
H   0.81708  1.74578 -1.49526
H  -0.31067  2.05720 -2.84044
H  -0.63285  0.73831 -1.69311
C  -3.06229  5.99054 -1.32699
H  -4.06906  6.17980 -0.90941
H  -3.17346  5.86237 -2.41832
H  -2.47770  6.91915 -1.17006
C   3.19203  0.65630 -0.63543
C   3.27315  2.01289 -0.16341
C   3.59186  3.05731 -1.05054
H   3.64748  4.08029 -0.65233
C   3.82310  2.84382 -2.42096
C   3.69005  1.52714 -2.89339
H   3.82664  1.32755 -3.96617
C   3.37644  0.44848 -2.04576
C   2.95144  2.34398  1.27672
H   1.88650  2.13322  1.49488
H   3.13132  3.41388  1.48081
H   3.54415  1.74684  1.98650
C   4.20611  3.98216 -3.34431
H   5.29835  4.17044 -3.34487
H   3.71922  4.92791 -3.04673
H   3.91680  3.77013 -4.38889
C   3.18427 -0.92542 -2.64645
H   3.23322 -0.88185 -3.74885
H   2.20974 -1.35635 -2.35635
H   3.94311 -1.64700 -2.29988
C   5.48631 -2.01634 -0.15093
H   5.07820 -2.83920 -0.76300
H   6.34030 -2.41740  0.42573
H   5.87904 -1.24539 -0.83685
C   3.40898 -2.74848  1.99973
H   2.62565 -2.42495  2.70799
H   4.19954 -3.24639  2.58992
H   2.97319 -3.50491  1.32580
C   5.18509 -0.26774  2.30014
H   5.64098  0.62347  1.83452
H   6.00551 -0.88641  2.70975
H   4.56580  0.07285  3.14808
C  -2.06364 -2.17504 -0.80464
C  -3.21200 -2.40422  0.04716
C  -4.50938 -2.04146 -0.37175
H  -5.35361 -2.24572  0.30379
C  -4.76565 -1.44449 -1.62450
C  -3.65500 -1.24629 -2.47388
H  -3.82052 -0.82644 -3.47670
C  -2.34571 -1.61655 -2.11041
C  -3.01919 -3.05647  1.39589
H  -2.15897 -2.62054  1.93115
H  -2.79677 -4.13409  1.28896
H  -3.92451 -2.96526  2.02093
C  -6.17529 -1.10928 -2.07234
H  -6.85001 -0.95209 -1.21215
H  -6.61709 -1.92324 -2.67954
H  -6.20495 -0.19801 -2.69638
C  -1.23027 -1.51648 -3.12511
H  -0.30293 -1.11859 -2.68421
H  -1.52025 -0.89297 -3.98794
H  -0.96900 -2.52273 -3.50423
C   1.55455 -4.10931 -1.35968
H   1.44055 -3.88810 -2.43491
H   2.05467 -5.08987 -1.26060
H   2.22266 -3.33906 -0.93702
C  -1.32376 -5.24471 -1.44676
H  -2.32170 -5.29524 -0.97789
H  -0.91703 -6.27113 -1.48652
H  -1.46381 -4.89657 -2.48481
C   0.15450 -4.91041  1.23348
H   0.77157 -4.27099  1.88768
H   0.68430 -5.87367  1.11376
H  -0.79167 -5.10861  1.76484
C  -1.79918  3.48460  1.31314
Si -1.03903  1.30476  2.89593
C  -0.01483 -1.00954  3.08899
H  -0.18390 -2.01372  2.61971
H   1.06890 -0.97615  3.35111
H  -0.53518 -1.09306  4.05927
```

VI
SCF (BP86) Energy = -1648.84517768
Enthalpy 0K = -1648.015235
Enthalpy 298K = -1647.951332
Free Energy 298K = -1648.115112
Lowest Frequency = 11.3977 cm^{-1}
Second Frequency = 16.7515 cm^{-1}
SCF (BP86-D3BJ) Energy = -1649.12069488
SCF (C6H6) Energy = -1648.88219122
SCF (BS2) Energy = -3718.59874663

```
Ca  0.32615 -0.89605  0.88029
K  -2.91663  1.46592 -0.06462
Si  3.67028 -2.27379  0.99638
Si -1.21252 -3.86945 -0.79775
N  -0.40070  1.48230  1.21906
N   2.63744 -1.07795  0.24124
N  -1.43958 -2.15817 -0.48163
C  -0.83323  3.86052  2.59389
H  -1.76319  4.33152  2.96561
H   0.00293  4.39636  3.08371
C  -2.40868  1.30503  3.55348
H  -3.26078  1.73345  2.98958
H  -2.56046  1.57990  4.61304
H  -2.45080  0.20633  3.47815
C   0.61630  1.54392  4.19463
H   0.76980  0.45448  4.24568
H   0.32149  1.90834  5.19567
H   1.57162  2.02121  3.92046
C  -0.40902  2.65558  0.45466
C  -0.24003  2.65714 -0.97371
C  -0.41814  3.85356 -1.69732
H  -0.28063  3.81988 -2.78769
C  -0.73796  5.08270 -1.08980
C  -0.87063  5.08832  0.31732
H  -1.10251  6.03194  0.83226
C   0.14805  1.38568 -1.68793
H   1.16962  1.06661 -1.40614
H   0.13948  1.51728 -2.78292
H  -0.52344  0.53227 -1.45963
C  -0.87411  6.35507 -1.90087
H  -1.68485  7.00248 -1.51880
H  -1.09293  6.13661 -2.96094
H   0.05197  6.96341 -1.88347
C   3.25784 -0.08928 -0.55063
C   3.66578  1.17889 -0.00720
C   4.28403  2.14243 -0.82427
H   4.58421  3.09720 -0.36911
C   4.51940  1.93512 -2.19502
C   4.07363  0.71947 -2.74127
H   4.20613  0.53635 -3.81766
C   3.45054 -0.27453 -1.96423
C   3.37849  1.51347  1.43861
H   2.28739  1.53820  1.61986
H   3.78883  2.50489  1.70007
H   3.79376  0.76709  2.13364
C   5.22951  2.97189 -3.04114
H   6.33225  2.87076 -2.99051
H   4.98820  3.99892 -2.71322
H   4.94886  2.88576 -4.10600
C   2.92404 -1.51768 -2.64184
H   3.09462 -1.47514 -3.73229
H   1.84030 -1.63550 -2.46239
H   3.39236 -2.43821 -2.25565
C   5.03832 -2.97939 -0.16216
H   4.62629 -3.70025 -0.88962
H   5.81535 -3.50319  0.42428
H   5.53179 -2.17197 -0.73055
C   2.61097 -3.74338  1.59607
H   1.80058 -3.40450  2.26439
H   3.23622 -4.45578  2.16450
H   2.16060 -4.29197  0.75187
C   4.64351 -1.67619  2.54959
H   5.36408 -0.87706  2.30156
H   5.21558 -2.51297  2.99320
H   3.95708 -1.29003  3.32319
C  -2.67960 -1.58550 -0.70242
C  -3.67489 -1.48053  0.34809
C  -4.91315 -0.84953  0.11193
H  -5.64764 -0.81681  0.93051
C  -5.25737 -0.28751 -1.13849
C  -4.29759 -0.38959 -2.16836
H  -4.54085  0.01269 -3.16319
C  -3.05087 -1.02412 -1.98692
C  -3.38745 -2.09736  1.69633
H  -2.37128 -1.85306  2.05884
H  -3.41373 -3.20081  1.63388
H  -4.13416 -1.78566  2.44755
C  -6.62318  0.32431 -1.38421
H  -7.03375  0.79635 -0.47342
H  -7.36119 -0.43617 -1.70613
H  -6.59188  1.09164 -2.17833
C  -2.10632 -1.16294 -3.15916
H  -1.05757 -1.09636 -2.82905
H  -2.30441 -0.40326 -3.93562
H  -2.21182 -2.15646 -3.63430
C   0.37454 -4.17264 -1.81596
H   0.27767 -3.76242 -2.83590
H   0.60130 -5.25120 -1.89859
H   1.23996 -3.67831 -1.34265
C  -2.69335 -4.62441 -1.76273
H  -3.64500 -4.45482 -1.22960
H  -2.55897 -5.71594 -1.87042
H  -2.79820 -4.19772 -2.77478
C  -1.04345 -4.93362  0.78809
H  -0.29905 -4.50369  1.47801
H  -0.72681 -5.96407  0.54114
H  -2.00093 -4.99579  1.33403
C  -0.70954  3.92555  1.07935
Si -0.72164  1.95996  2.91418
C  -0.05959 -1.76687  3.17125
H  -0.33867 -2.84448  3.11153
H   0.89979 -1.75211  3.74114
H  -0.80831 -1.32629  3.86707
```

VII
SCF (BP86) Energy = -1648.83904784
Enthalpy 0K = -1648.008750
Enthalpy 298K = -1647.944978
Free Energy 298K = -1648.109218
Lowest Frequency = 13.3713 cm^{-1}
Second Frequency = 17.2411 cm^{-1}
SCF (BP86-D3BJ) Energy = -1649.11902153
SCF (C6H6) Energy = -1648.87478177
SCF (BS2) Energy = -3718.59135137

```
Ca -0.20654 -0.68966  0.79710
K  -2.04288  2.04380  1.45028
Si  1.88393 -3.55353  1.23824
Si -3.22668 -2.69321 -0.29949
N   0.66912  1.63245  1.24170
N   1.59015 -2.13463  0.26896
N  -2.44304 -1.12410 -0.38298
C   2.21334  3.57718  2.51016
H   1.91762  4.49610  3.05128
H   3.27683  3.39649  2.76071
C  -0.34637  2.69252  4.02611
H  -0.84558  3.57862  3.58487
H   0.06987  3.03033  4.99281
H  -1.09136  1.90925  4.24772
C   2.02655  0.73677  3.94136
H   1.43202 -0.18711  4.02200
H   2.22662  1.10719  4.96330
H   2.99043  0.48725  3.46961
C   1.25532  2.61795  0.42101
C   1.14282  2.60239 -1.00479
C   1.68880  3.65414 -1.76259
H   1.58073  3.61304 -2.85586
C   2.38158  4.73524 -1.18579
C   2.54742  4.71704  0.21535
H   3.11909  5.52106  0.70124
C   0.48641  1.43145 -1.68589
H   1.06154  0.49697 -1.51920
H   0.42905  1.57528 -2.77753
H  -0.55390  1.26505 -1.34032
C   2.91838  5.87185 -2.03114
H   2.21849  6.73064 -2.08346
H   3.10448  5.54830 -3.07062
H   3.87062  6.26388 -1.62950
C   2.47179 -1.79261 -0.78277
C   3.59659 -0.92172 -0.57372
C   4.45305 -0.59085 -1.63897
H   5.30219  0.07699 -1.43625
C   4.25288 -1.06570 -2.94684
C   3.12618 -1.87575 -3.16434
H   2.91717 -2.24015 -4.18058
C   2.24262 -2.23670 -2.12995
C   3.84095 -0.30389  0.78303
H   2.97261  0.30895  1.08641
H   4.73247  0.34664  0.76189
H   3.98138 -1.06312  1.56957
C   5.21410 -0.72593 -4.06739
H   6.08388 -1.41241 -4.09506
H   5.61809  0.29665 -3.96046
H   4.72269 -0.79075 -5.05428
C   1.02648 -3.06812 -2.46321
H   0.93949 -3.21671 -3.55403
H   0.10068 -2.58812 -2.10203
H   1.05367 -4.06203 -1.98549
C   1.99758 -5.20943  0.26313
H   1.03015 -5.47674 -0.19587
H   2.29651 -6.03996  0.92925
H   2.74802 -5.14191 -0.54403
C   0.43066 -3.73204  2.47025
H   0.28262 -2.82815  3.08756
H   0.62455 -4.57101  3.16275
H  -0.52036 -3.94950  1.95330
C   3.49030 -3.54054  2.30140
H   4.38823 -3.40439  1.67349
H   3.60421 -4.49849  2.84261
H   3.47514 -2.73019  3.05073
C  -3.15370 -0.02464 -0.86131
C  -4.15214  0.68236 -0.09100
C  -4.72440  1.88102 -0.57257
H  -5.47737  2.39073  0.04915
C  -4.40215  2.42127 -1.83007
C  -3.50444  1.68197 -2.62803
H  -3.27694  2.03920 -3.64245
C  -2.90774  0.48892 -2.19082
C  -4.60175  0.13257  1.24809
H  -3.75778 -0.29529  1.81763
H  -5.32513 -0.69391  1.11977
H  -5.11267  0.90522  1.85427
C  -5.00527  3.72253 -2.31797
H  -5.84205  4.04343 -1.67311
H  -5.39578  3.63109 -3.34824
H  -4.26723  4.54791 -2.33365
C  -2.05343 -0.31839 -3.13965
H  -1.06346 -0.55300 -2.71763
H  -1.91447  0.20818 -4.09945
H  -2.52568 -1.29680 -3.34590
C  -2.08594 -4.06416 -0.98733
H  -1.93060 -3.95226 -2.07347
H  -2.51704 -5.06468 -0.80064
H  -1.09225 -4.02761 -0.50900
C  -4.84062 -2.71763 -1.34149
H  -5.56921 -1.96645 -0.99101
H  -5.32386 -3.70965 -1.28608
H  -4.63247 -2.50138 -2.40366
C  -3.70431 -3.33369  1.45108
H  -2.82493 -3.36882  2.11692
H  -4.10751 -4.36100  1.37639
H  -4.46435 -2.70716  1.94602
C   2.01562  3.69192  1.00622
Si  1.10866  2.05766  2.92197
C  -1.40649 -0.51370  3.03383
H  -1.89014 -1.51743  3.01873
H  -0.69688 -0.56697  3.89101
H  -2.22707  0.13947  3.42622
```

S7. SQUID Magnetometry and EPR measurements

S7.1 SQUID Magnetometry measurements

Variable temperature magnetic susceptibility data were collected on powdered samples, under an applied field of 0.5 T. Field dependent magnetisation data were collected at 2 K by varying the magnetic field from 0 to 7 T. Data were corrected for the diamagnetism of the samples and sample holders by measuring equivalent quantities of diamagnetic precursor and the sample holder under similar experimental conditions to compound **2**. Care was taken to ensure complete thermalisation of the sample before each data point was measured by employing delays at each temperature point and the sample was held at 2 K for 60 minutes before isothermal magnetisation data were recorded to account for slow thermal equilibration of the sample.

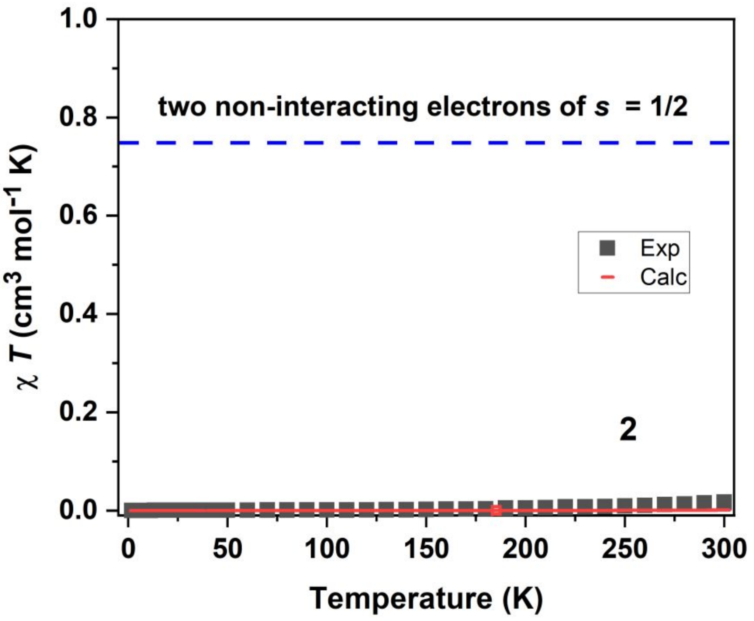

Figure S28: Plot of χT *vs*. T for **2** (χ = molar magnetic susceptibility), measured under an applied field of 0.5 T, supporting a singlet ground state, and strong antiferromagnetic coupling between electrons in **2**, in agreement with AIRSS data. The blue dashed line marks the expected χT value for the case that the two anionic electrons of **2** did not interact with each other (assuming $g = 2$), while the red line is a simulation using the Bleaney-Bowers equation,[27] and assuming antiferromagnetic exchange coupling of –4409 K (ca. –3064 cm^{-1}), equivalent to a single-triplet separation of 0.38 eV.

S7.2 Electron paramagnetic resonance (EPR) spectroscopy

The electron paramagnetic resonance spectra were recorded under magnetic field modulation of 100 kHz, modulation amplitude of 1 G, and microwave power of either 0.02 (40 dB), 0.2 (30 dB) or 0.6325 mW (25 dB). Field corrections were applied using Bruker strong pitch (g = 2.0028) as a reference. Spectra of an empty quartz tube used as sample holder were measured before recording the sample spectra in order to ensure the purity of the obtained signals. To determine the effective percentage of electron spins of **2** that are detectable in the experimental temperature range (5 – 293 K), a sample of known mass (36.2 mg) of **2** was measured under similar conditions as TEMPO (2,2,6,6-tetramethylpiperidine-1-oxyl) reference samples of known spin concentrations (0.074 – 0.37 mM; 4.5×10^{15} to 2.3×10^{16} spins per sample). Both first harmonic (FH) and second harmonic (SH) signals were baselined with a first or second order polynomial function before integration to extract signal intensities or prior to simulations. Simulations of the FH spectra used the EasySpin 6.0.0 software package implemented within MATLAB.[26] Thermal dependence of the EPR signal intensity was modelled using the Bleaney-Bowers equation[27] in order to establish the nature and the strength of magnetic exchange that may occur between the unpaired electrons of **2**, and to probe the triplet-singlet energy gap. To allow comparison between species, SH signal intensities were extracted as the absolute polygon areas of the spectral feature being monitored.

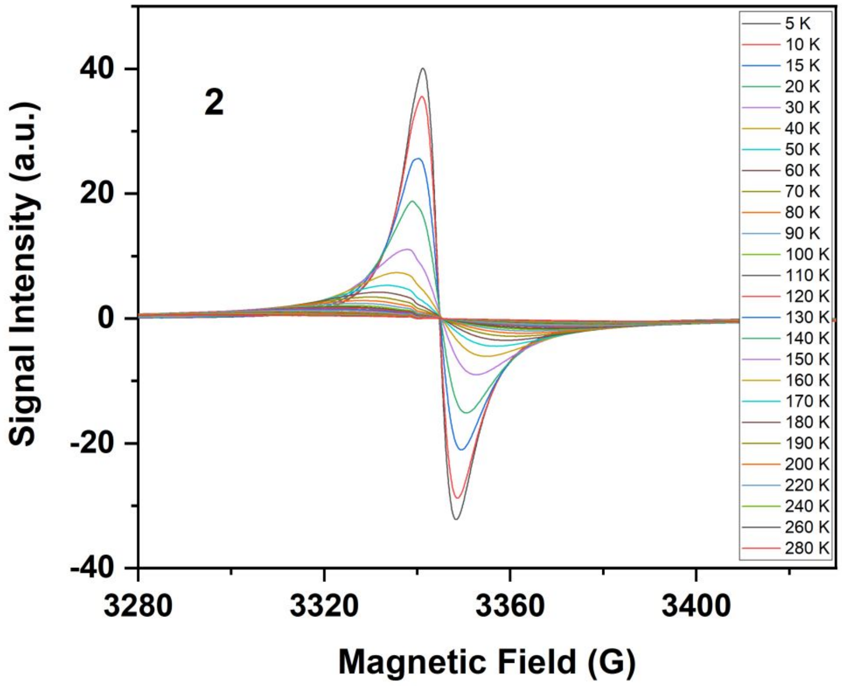

Figure S29: X-band EPR spectra for **2** at temperatures between 5 and 280 K, measured at the frequency of 9.36 GHz, with field modulation amplitude of 1 G, and attenuation of 30 dB.

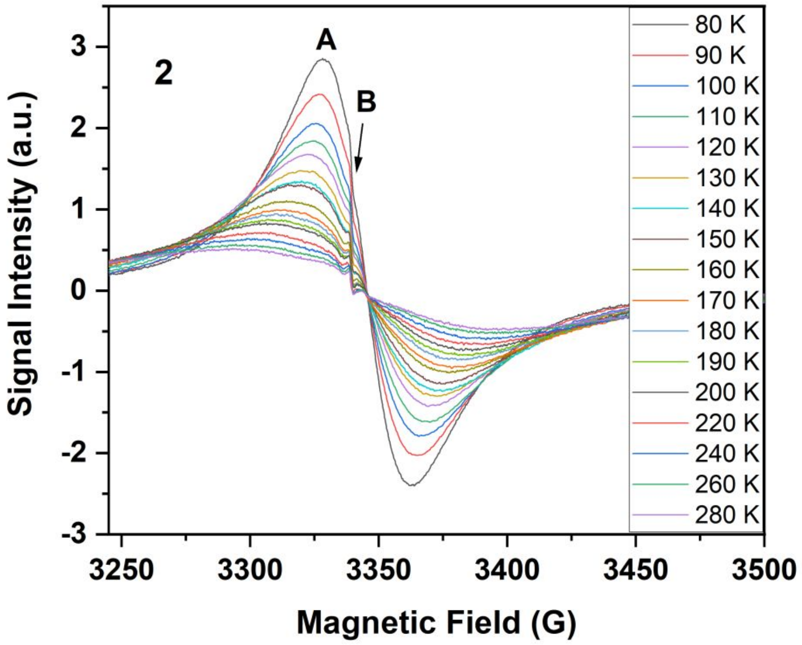

Figure S30: X-band EPR spectra for **2** at temperatures between 80 and 280 K, emphasizing the presence of unpaired electrons in two different environments (**A** and **B**), measured at the frequency of 9.36 GHz, with field modulation amplitude of 1 G, and microwave attenuation of 30 dB.

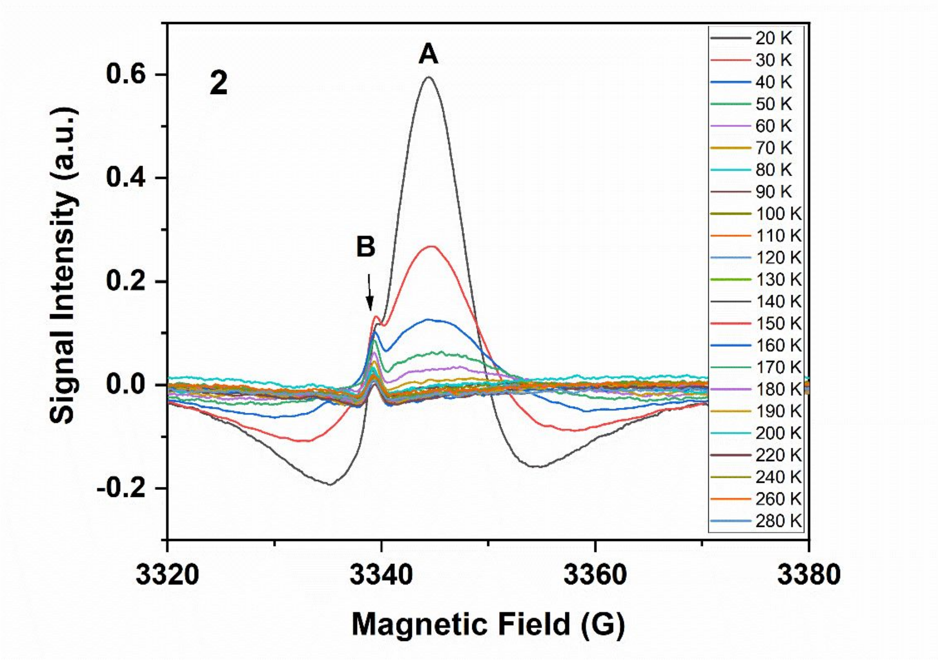

Figure S31: Second Harmonic (SH) X-band EPR spectra for **2** at temperatures between 20 and 280 K, emphasizing the presence of unpaired electron spins in two different environments (**A** and **B**), measured at the frequency of 9.36 GHz, with field modulation amplitude of 1 G, and microwave attenuation of 25 dB.

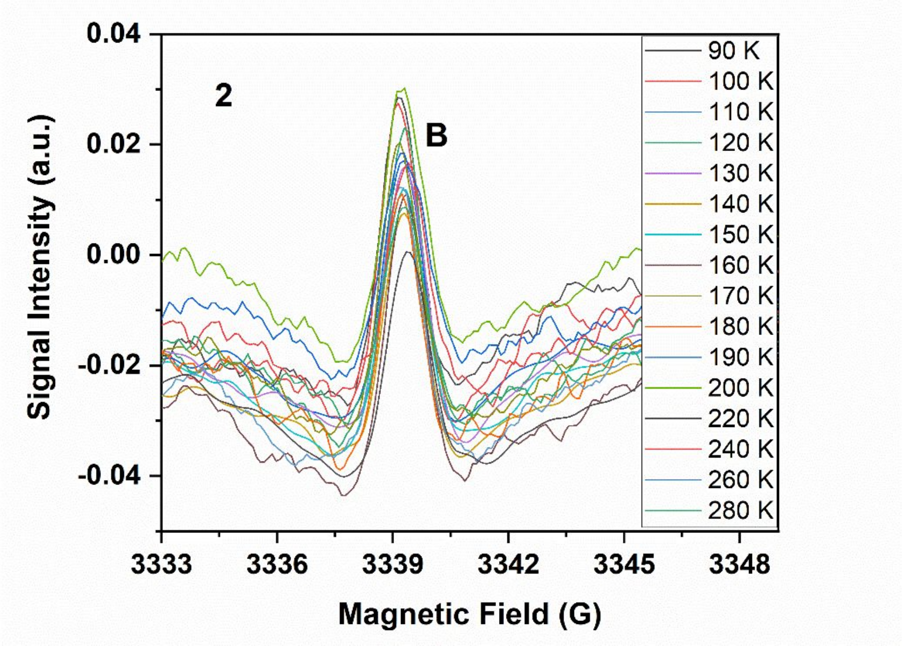

Figure S32: A zoomed image of the Second Harmonic (SH) X-band EPR spectra for **2** presented in Figure S30, for the field region 3333-3349 G, and temperatures from 90 to 280 K, emphasizing the limited change in signal intensity for signal **B** as a function of temperature.

Spectra were measured at a microwave frequency of ca. 9.36 GHz, a field modulation amplitude of 1 G, and microwave attenuation of 25 dB.

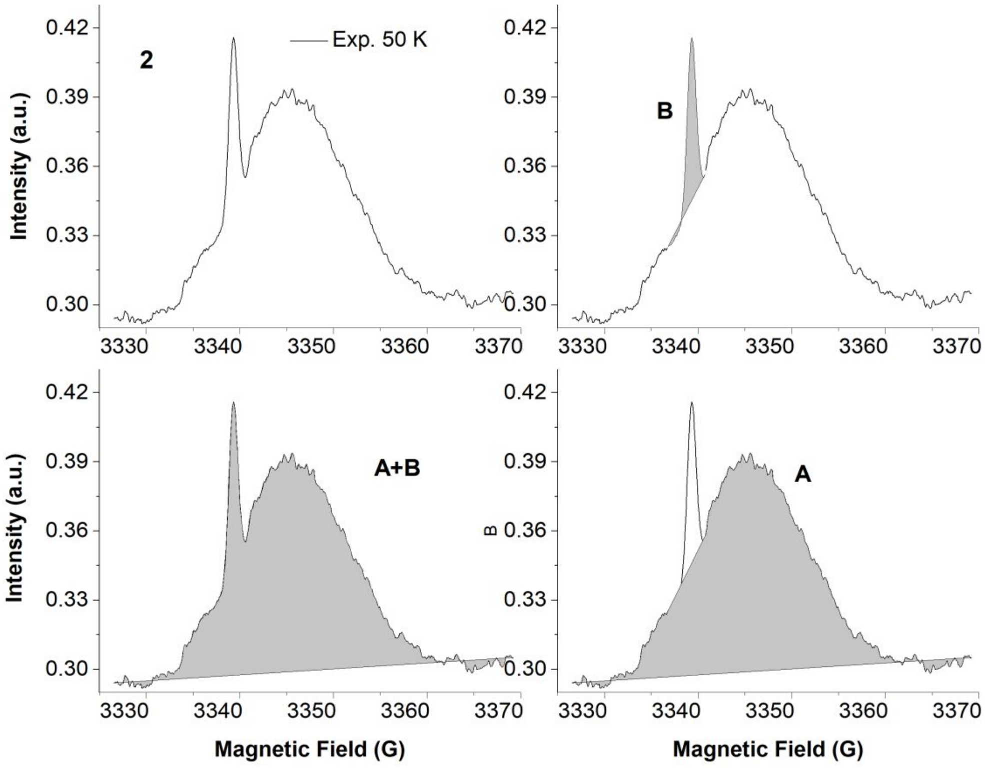

Figure S33: Deconvolution of a measured SH signal for **2** (9.36 GHz, 50 K) into its components (**A** and **B**) to establish the ratio between them, using the method of calculated absolute polygon areas (highlighted in grey): 1.3635 (**A+B**), 1.2907 (**A**) and 0.0756 (**B**), n_B/n_A = 0.0586.

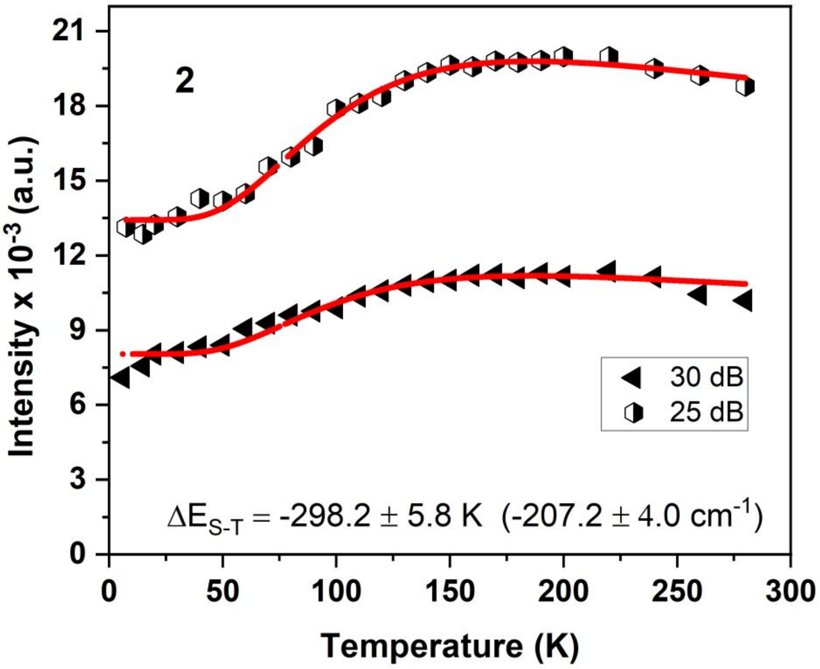

Figure S34: Temperature dependence of the EPR intensity for **2**, at microwave powers of 25 and 30 dB, and their fits to the Bleaney-Bowers equation[27] with $\Delta E_{S\text{-}T} = 298 \pm 5.8$ K. Intensities are deduced by double integration of the recorded EPR signal.

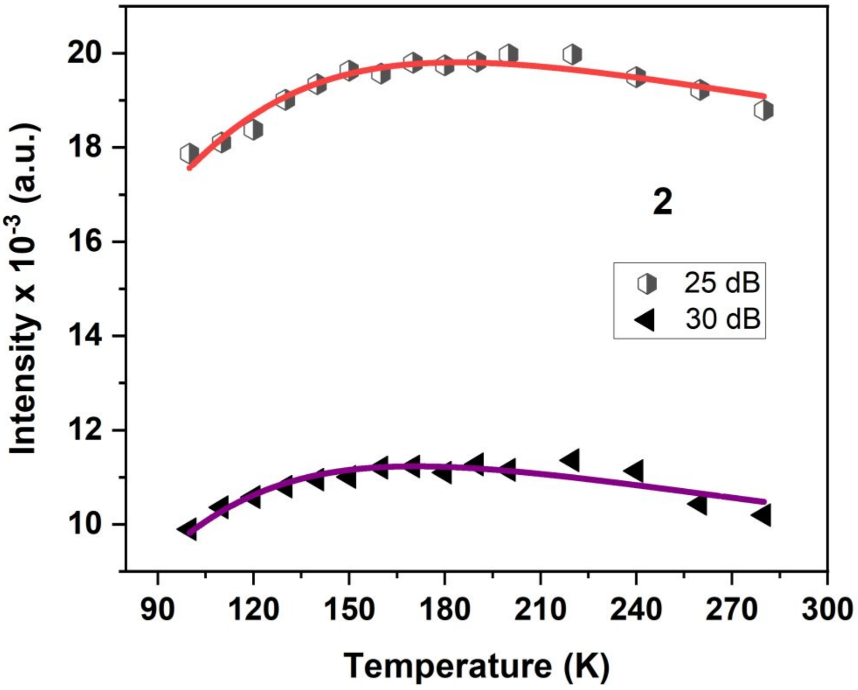

Figure S35: Temperature dependence of the EPR intensity for data recorded at 100–280 K, at microwave powers of 25 and 30 dB, and their fits to the Bleaney-Bowers equation[27] providing $\Delta E_{S\text{-}T} = 295 \pm 6.7$ K (25 dB) and 274.0 ± 6.3 K (30 dB).

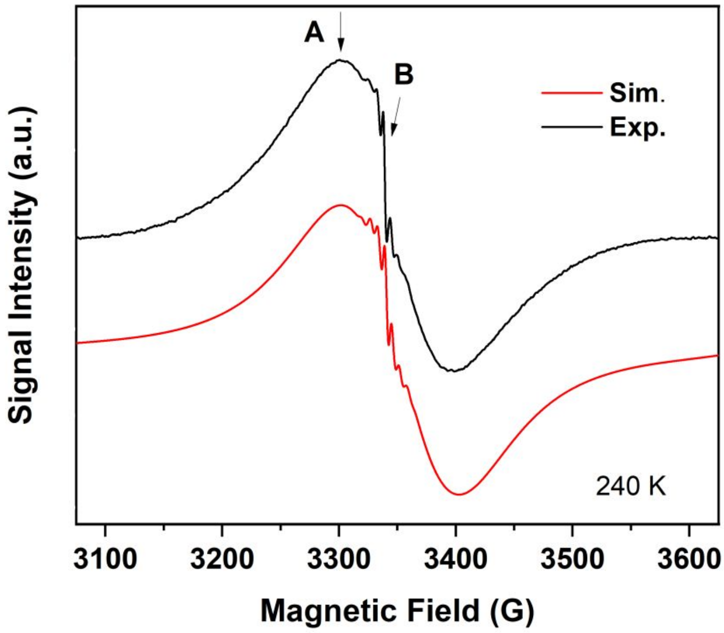

Figure S36: X-band EPR spectrum for **2′** (black) at 240 K and 9.36 GHz, and its simulation (red) considering two species: (**A**) s = ½, $g_{x,y}$ = 1.9964; g_z =1.9939, and (**B**) *s* =1/2, interacting with two K atoms, each containing ^{39}K (93.3%, I = 3/2) and ^{41}K (6.73%, I = 3/2)), with *g* = 2.0024, $A^{K}_{x,y}$ = 9.7 MHz, A^{K}_{z} = 19.5 MHz. Linewidth [Gaussian Lorentzian] /weight: [6 13]/ 99.8 (**A**), [0.49 0] / 0.2 (**B**).

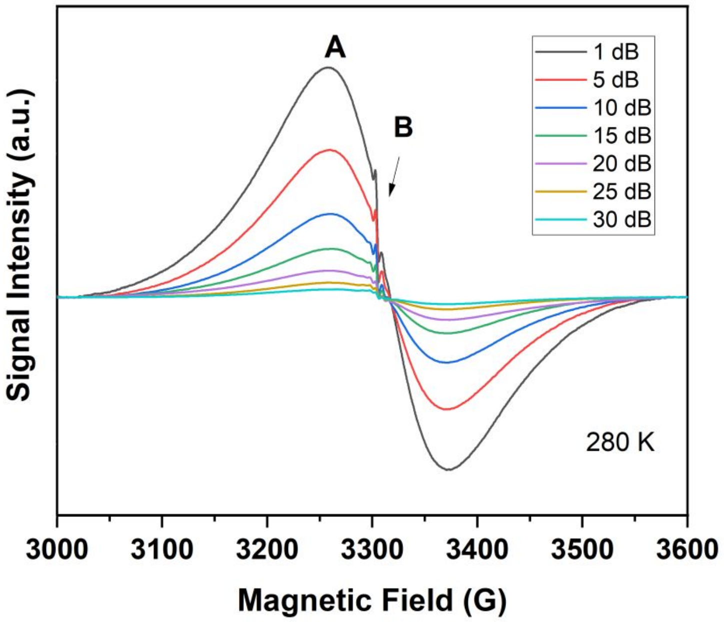

Figure S37: X-band EPR spectra Ffirst Harmonic) for **2′** at 280 K, measured at the frequency of 9.36 GHz, with field modulation amplitude of 1 G and attenuation of 1 to 30 dB, highlighting the presence of two species, **A** and **B**.

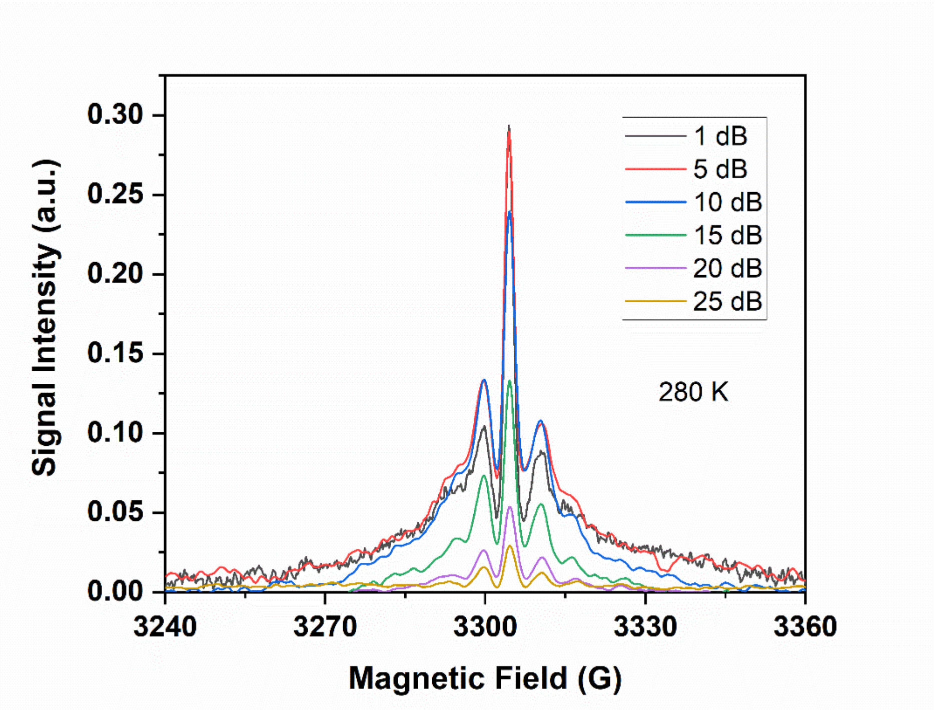

Figure S38: Second Harmonic (SH) X-band EPR spectra for **2′** at 280 K, measured at 9.36 GHz, 1 G modulation amplitude and microwave field attenuations from 1 to 25 dB, showing the hyperfine splitting from two K ions (as modelled in Figure S37).

S8. References

(1) Sheldrick, G. M. *Program for Area Detector Absorption Correction*, Institute for Inorganic Chemistry, University of Göttingen: Göttingen, Germany, 1996.

(2) Sheldrick, G. M. A Short History of SHELX. *Acta Cryst. A* **2008**, *64* (1), 112–122. https://doi.org/10.1107/S0108767307043930.

(3) Sheldrick, G. M. SHELXT – Integrated Space-Group and Crystal-Structure Determination. *Acta Cryst. A* **2015**, *71* (1), 3–8. https://doi.org/10.1107/S2053273314026370.

(4) Dolomanov, O. V.; Bourhis, L. J.; Gildea, R. J.; Howard, J. A. K.; Puschmann, H. OLEX2: A Complete Structure Solution, Refinement and Analysis Program. *J. Appl. Cryst.* **2009**, *42* (2), 339–341. https://doi.org/10.1107/S0021889808042726.

(5) *CrysAlisPro Software System*, Version 1.171.42a, Rigaku Corporation, Wroclaw, Poland, 2024.

(6) Le Bail, A.; Duroy, H.; Fourquet, J. L. Ab-Initio Structure Determination of $LiSbWO_6$ by X-Ray Powder Diffraction. *Mat. Res. Bull.* **1988**, *23* (3), 447–452. https://doi.org/10.1016/0025-5408(88)90019-0.

(7) Petříček, V.; Palatinus, L.; Plášil, J.; Dušek, M. Jana2020 – a New Version of the Crystallographic Computing System Jana. *Z. Kristallogr.* **2023**, *238* (7–8), 271–282. https://doi.org/10.1515/zkri-2023-0005.

(8) Davison, N.; Quirk, J. A.; Tuna, F.; Collison, D.; McMullin, C. L.; Michaels, H.; Morritt, G. H.; Waddell, P. G.; Gould, J. A.; Freitag, M.; Dawson, J. A.; Lu, E. A Room-Temperature-Stable Electride and Its Reactivity: Reductive Benzene/Pyridine Couplings and Solvent-Free Birch Reductions. *Chem* **2023**, *9*, 576–591. https://doi.org/10.1016/j.chempr.2022.11.006.

(9) Pickard, C. J.; Needs, R. J. Ab Initio Random Structure Searching. *J. Phys. Condens. Matter* **2011**, *23* (5), 053201. https://doi.org/10.1088/0953-8984/23/5/053201.

(10) Batatia, I.; Benner, P.; Chiang, Y.; Elena, A. M.; Kovács, D. P.; Riebesell, J.; Advincula, X. R.; Asta, M.; Avaylon, M.; Baldwin, W. J.; Berger, F.; Bernstein, N.; Bhowmik, A.; Blau, S. M.; Cărare, V.; Darby, J. P.; De, S.; Della Pia, F.; Deringer, V. L.; Elijošius, R.; El-Machachi, Z.; Falcioni, F.; Fako, E.; Ferrari, A. C.; Genreith-Schriever, A.; George, J.; Goodall, R. E. A.; Grey, C. P.; Grigorev, P.; Han, S.; Handley, W.; Heenen, H. H.; Hermansson, K.; Holm, C.; Jaafar, J.; Hofmann, S.; Jakob, K. S.; Jung, H.; Kapil, V.; Kaplan, A. D.; Karimitari, N.; Kermode, J. R.; Kroupa, N.; Kullgren, J.; Kuner, M. C.; Kuryla, D.; Liepuoniute, G.; Margraf, J. T.; Magdău, I.-B.; Michaelides, A.; Moore, J. H.;

Naik, A. A.; Niblett, S. P.; Norwood, S. W.; O'Neill, N.; Ortner, C.; Persson, K. A.; Reuter, K.; Rosen, A. S.; Schaaf, L. L.; Schran, C.; Shi, B. X.; Sivonxay, E.; Stenczel, T. K.; Svahn, V.; Sutton, C.; Swinburne, T. D.; Tilly, J.; van der Oord, C.; Varga-Umbrich, E.; Vegge, T.; Vondrák, M.; Wang, Y.; Witt, W. C.; Zills, F.; Csányi, G. A Foundation Model for Atomistic Materials Chemistry. arXiv March 1, 2024. https://doi.org/10.48550/arXiv.2401.00096.

(11) Kresse, G.; Joubert, D. From Ultrasoft Pseudopotentials to the Projector Augmented-Wave Method. *Phys. Rev. B* **1999**, *59* (3), 1758–1775. https://doi.org/10.1103/PhysRevB.59.1758.

(12) Krukau, A. V.; Vydrov, O. A.; Izmaylov, A. F.; Scuseria, G. E. Influence of the Exchange Screening Parameter on the Performance of Screened Hybrid Functionals. *J. Chem. Phys.* **2006**, *125* (22), 224106. https://doi.org/10.1063/1.2404663.

(13) Kühne, T. D.; Iannuzzi, M.; Del Ben, M.; Rybkin, V. V.; Seewald, P.; Stein, F.; Laino, T.; Khaliullin, R. Z.; Schütt, O.; Schiffmann, F.; Golze, D.; Wilhelm, J.; Chulkov, S.; Bani-Hashemian, M. H.; Weber, V.; Borštnik, U.; Taillefumier, M.; Jakobovits, A. S.; Lazzaro, A.; Pabst, H.; Müller, T.; Schade, R.; Guidon, M.; Andermatt, S.; Holmberg, N.; Schenter, G. K.; Hehn, A.; Bussy, A.; Belleflamme, F.; Tabacchi, G.; Glöß, A.; Lass, M.; Bethune, I.; Mundy, C. J.; Plessl, C.; Watkins, M.; VandeVondele, J.; Krack, M.; Hutter, J. CP2K: An Electronic Structure and Molecular Dynamics Software Package - Quickstep: Efficient and Accurate Electronic Structure Calculations. *J. Chem. Phys.* **2020**, *152* (19), 194103. https://doi.org/10.1063/5.0007045.

(14) Bursch, M.; Neugebauer, H.; Ehlert, S.; Grimme, S. Dispersion Corrected r2SCAN Based Global Hybrid Functionals: r2SCANh, r2SCAN0, and r2SCAN50. *J. Chem. Phys.* **2022**, *156* (13), 134105. https://doi.org/10.1063/5.0086040.

(15) Frisch, M. J.; Trucks, G. W.; Schlegel, H. B.; Scuseria, G. E.; Robb, M. A.; Cheeseman, J. R.; Scalmani, G.; Barone, V.; Petersson, G. A.; Nakatsuji, H.; Li, X.; Caricato, M.; Marenich, A. V.; Bloino, J.; Janesko, B. G.; Gomperts, R.; Mennucci, B.; Hratchian, H. P.; Ortiz, J. V.; Izmaylov, A. F.; Sonnenberg, J. L.; Williams; Ding, F.; Lipparini, F.; Egidi, F.; Goings, J.; Peng, B.; Petrone, A.; Henderson, T.; Ranasinghe, D.; Zakrzewski, V. G.; Gao, J.; Rega, N.; Zheng, G.; Liang, W.; Hada, M.; Ehara, M.; Toyota, K.; Fukuda, R.; Hasegawa, J.; Ishida, M.; Nakajima, T.; Honda, Y.; Kitao, O.; Nakai, H.; Vreven, T.; Throssell, K.; Montgomery Jr., J. A.; Peralta, J. E.; Ogliaro, F.; Bearpark, M. J.; Heyd, J. J.; Brothers, E. N.; Kudin, K. N.; Staroverov, V. N.; Keith, T. A.; Kobayashi, R.; Normand, J.; Raghavachari, K.; Rendell, A. P.; Burant, J. C.; Iyengar, S. S.; Tomasi, J.;

Cossi, M.; Millam, J. M.; Klene, M.; Adamo, C.; Cammi, R.; Ochterski, J. W.; Martin, R. L.; Morokuma, K.; Farkas, O.; Foresman, J. B.; Fox, D. J. *Gaussian 16 Rev. C.01*, 2016.

(16) Andrae, D.; Häußermann, U.; Dolg, M.; Stoll, H.; Preuß, H. Energy-Adjustedab Initio Pseudopotentials for the Second and Third Row Transition Elements. *Theoret. Chim. Acta* **1990**, *77* (2), 123–141. https://doi.org/10.1007/BF01114537.

(17) Hariharan, P. C.; Pople, J. A. The Influence of Polarization Functions on Molecular Orbital Hydrogenation Energies. *Theoret. Chim. Acta* **1973**, *28* (3), 213–222. https://doi.org/10.1007/BF00533485.

(18) Hehre, W. J.; Ditchfield, R.; Pople, J. A. Self—Consistent Molecular Orbital Methods. XII. Further Extensions of Gaussian—Type Basis Sets for Use in Molecular Orbital Studies of Organic Molecules. *J. Chem. Phys.* **1972**, *56*, 2257–2261. https://doi.org/10.1063/1.1677527.

(19) Becke, A. D. Density-Functional Exchange-Energy Approximation with Correct Asymptotic Behavior. *Phys. Rev. A* **1988**, *38* (6), 3098–3100. https://doi.org/10.1103/PhysRevA.38.3098.

(20) Perdew, J. P. Density-Functional Approximation for the Correlation Energy of the Inhomogeneous Electron Gas. *Phys. Rev. B* **1986**, *33* (12), 8822–8824. https://doi.org/10.1103/PhysRevB.33.8822.

(21) Tomasi, J.; Mennucci, B.; Cammi, R. Quantum Mechanical Continuum Solvation Models. *Chem. Rev.* **2005**, *105* (8), 2999–3094. https://doi.org/10.1021/cr9904009.

(22) Grimme, S.; Ehrlich, S.; Goerigk, L. Effect of the Damping Function in Dispersion Corrected Density Functional Theory. *J. Comp. Chem.* **2011**, *32* (7), 1456–1465. https://doi.org/10.1002/jcc.21759.

(23) Keith, T. A. *AIMA11*, Version 19.10.12, TK Gristmill Software, Overland Park KS, USA, 2019.

(24) Gledening, E. D.; Reed, A. E.; Carpenter, J. E.; Weinhold, F. *NBO 3.1*, Theoretical Chemistry Institute, University of Wisconsin, Madison, USA, 2003.

(25) Gledening, E. D.; Badenhoop, J. K.; Reed, A. E.; Carpenter, J. E.; Bohmann, J. A.; Morales, C. M.; Karafiloglou, P.; Weinhold, F. *NBO 7.0*, Theoretical Chemistry Institute, University of Wisconsin, Madison, USA, 2018.

(26) Stoll, S.; Schweiger, A. EasySpin, a Comprehensive Software Package for Spectral Simulation and Analysis in EPR. *J. Magn. Res.* **2006**, *178* (1), 42–55. https://doi.org/10.1016/j.jmr.2005.08.013.

(27) Bleaney, B.; Bowers, K. D. Anomalous Paramagnetism of Copper Acetate. *Proc. R. Soc. London, Ser. A* **1997**, *214* (1119), 451–465. https://doi.org/10.1098/rspa.1952.0181.
